# The Development and Application of a HPTLC-Derived Database for the Identification of Phenolics in Honey

**DOI:** 10.3390/molecules27196651

**Published:** 2022-10-06

**Authors:** Ivan Lozada Lawag, Tomislav Sostaric, Lee Yong Lim, Katherine Hammer, Cornelia Locher

**Affiliations:** 1 Cooperative Research Centre for Honey Bee Products Limited (CRC HBP), University of Western Australia, Agriculture North M085, Perth, WA 6009, Australia; 2 Division of Pharmacy, School of Allied Health, University of Western Australia, Curnow Building M315, Perth, WA 6009, Australia; 3 School of Biomedical Sciences, University of Western Australia, M Block QEII Medical Centre, Monash Ave., Perth, WA 6009, Australia

**Keywords:** HPTLC, HPLC-DAD, Manuka honey, *Leptospermum scoparium*, database, biomarkers, phenolic compounds, phenolic determination

## Abstract

This study reports on the development and validation of a HPTLC-derived database to identify phenolic compounds in honey. Two database sets are developed to contain the profiles of 107 standard compounds. Rich data in the form of Rf values, colour hues (H°) at 254 nm and 366 nm, at 366 nm after derivatising with natural product PEG reagent, and at 366 nm and white light after derivatising with vanillin–sulfuric acid reagent, λ max and λ min values in their fluorescence and λ max values in their UV-Vis spectra as well as λ max values in their fluorescence and UV-Vis spectra after derivatisation are used as filtering parameters to identify potential matches in a honey sample. A spectral overlay system is also developed to confirm these matches. The adopted filtering approach is used to validate the database application using positive and negative controls and also by comparing matches with those identified via HPLC-DAD. Manuka honey is used as the test honey and leptosperine, mandelic acid, kojic acid, lepteridine, gallic acid, epigallocatechin gallate, 2,3,4-trihydroxybenzoic acid, o-anisic acid and methyl syringate are identified in the honey using the HPTLC-derived database.

## 1. Introduction

Honey is an amber-coloured and viscous, natural substance produced by bees from the nectar of flowers (blossom honey) or the exudation of living parts of plants or insect excretions (honeydew honey) [1,2,3]. It is primarily composed of sugars (mostly glucose and fructose, which constitute approximately 60–85% of the honey’s total weight) and water (18–22%), as well as minor constituents (approximately 3%), such as amino acids, certain enzymes and other proteins, carotenoid-like substances, Maillard reaction products, minerals, vitamins, organic acids, phenolic acids and polyphenolic compounds, including flavonoids [1,3,4,5,6]. Honey is commonly considered a natural food supplement as some of the above-mentioned minor components can contribute not only to honey’s organoleptic characteristics [7], but also to its nutritional and health benefits [1,3,4,5,6]. Typically, honeys are offered as being either multifloral (produced by bees using nectar from many floral sources) or monofloral (derived from the nectar of predominantly one flower spices), and their botanical origin affects the quality and price [8].

Despite their relatively minor presence, phenolic compounds are one of the most studied honey constituents due to their well-known biological activities [7,9]. They are, furthermore, reported to influence the organoleptic characteristics of a honey, such as its colour, taste and aroma [3,7,8,10,11,12]. Phenolic compounds, such as flavonoids and phenolic acids, have also been identified as potential chemical markers for authenticating the geographical and botanical origin, and the quality of honey [1,4,13,14]. Moreover, they can also be used to monitor honey quality in order to choose the best processing practices [15]. A plethora of phenolic compounds have already been identified in a variety of honeys around the world [16]. The most frequently identified phenolics in honey belong to the class of hydroxycinnamic acid derivatives, hydroxybenzoic acid derivatives, flavonols, flavones and flavanones [16].

The regulation of honey quality is essential to ensure consumers receive a high-quality product at a price that is reflective of the botanical origin (e.g., monofloral/multifloral) and bioactivity levels of the honey [5,8,17]. Manuka honey, for example, is one the most popular honeys worldwide due to its medicinal qualities, in particular, its exemplary antibacterial properties that can be correlated to its methylglyoxal levels (MGO) [18]. Because of this, the price of Manuka honey has been increasing, also leading to incidences of fraud and adulteration. In order to address this, the Ministry of Primary Industries (MPI), New Zealand, published a guideline for Manuka honey authentication by utilising 2′-methoxyacetophenone, (2′-MAPh), 2-methoxybenzoic acid or o-anisisc acid, (2-MB), 3-phenyllactic acid (3-PLA) and 4-hydroxyphenyllactic acid (4-HPLA) as biomarker compounds [19]. An extensive analysis of phenolic compounds present in Manuka honey has been conducted by employing various chromatographic and spectroscopic techniques. HPLC using a photodiode array (DAD/PDA) or UV detectors is the most commonly used [20,21,22,23,24,25,26,27,28,29,30], followed by LC-MS [19,31,32,33,34], HPTLC [20] and fluorescence spectroscopy [35].

Using high-performance thin-layer chromatography (HPTLC), Stanek and Jasicka-Misiak in 2018 reported that Manuka honey samples contained rosmarinic acid, ellagic acid, *p*-coumaric acid and myricetin. The method was also employed in the phenolic-compound determination in other honeys, for which chlorogenic acid, caffeic acid, ferulic acid, 3,4-dihydroxybenzoic acid, abscisic acid and myricetin were identified in willow honey, while chlorogenic acid, caffeic acid, *p*-coumaric acid, abscisic acid, myricetin and chrysin were found in heather honey [20]. Furthermore, in 2020, Guzelmeric et al. identified caffeic acid as a biomarker for pine honey using HPTLC [5]. However, in these studies, only Rf values and colour were employed as the bases for compound matching, and no spectral scans were performed to confirm the identity of the compound. Furthermore, in these studies, only one derivatisation was used to derive the colour of the respective HPTLC bands.

HPTLC uses a high-grade stationary phase (commonly HPTLC-grade silica gel with a fluorescence indicator) alongside a suitable mobile-phase system, and the sample application is conducted semi-automatically. The instrumentation also includes a derivatiser that allows for the possible application of a suitable derivatisation reagent and generated images (typically at 254 and 366 nm prior to derivatisation, as well as at 366 nm and white light after derivatisation) are automatically captured by an imaging device. A TLC scanner, which can record the UV-Vis and fluorescence spectra of individual compound bands, complements the set up. An advantage of this analytical approach over others is the richness of the data generated, which includes not only Rf and peak intensity values for individual bands, but also their respective colours (recorded as RGB values) at various light conditions prior to and also after derivatisation, as well as the possibility of capturing the UV-Vis and fluorescence spectra of individual compound bands, again prior to and after derivatisation with a suitable reagent. This study set out to develop and validate a HPTLC-derived database for the identification of phenolic compounds in honey. The database taps into the richness of HPTLC-generated data, beyond just Rf values and colour. Using this approach, it is anticipated that the identification of phenolic compounds that might act as marker compounds in a wide variety of honeys will add to the current suite of honey authentication methods.

## 2. Results

### 2.1. Database Development

To construct the four sub-databases (DB-1A, DB-1B, DB-2A, DB-2B), rich information was extracted from the HPTLC images of all 107 standards (Table 1).

#### 2.1.1. Retention Factor (Rf1 and Rf2 in MPA and MPB, Respectively)

MPB was selected as the mobile phase because prior studies of honey using HPTLC analysis employed this mobile phase, allowing for cross-references to previous work. MPA, with slightly higher polarity, was chosen to ensure that more polar phenolics were also adequately separated and detected [36,37,38].

Rf values obtained in toluene:ethyl acetate:formic acid (2:8:1, *v/v/v*) as the mobile phase (MPA) ranged from 0.017 to 0.756 with most of the standards presenting Rf values at around 0.600. Rf values obtained using the slightly lower polar mobile phase of toluene:ethyl acetate:formic acid (6:5:1, *v/v/v*) (MPB) ranged from 0.012 to 0.687 with most of the standards presenting Rf values at around 0.500.

6-Hydroxyflavone-β-D-glucoside (2), baicalin (5), vitexin (9), rutin (17), hesperidin (19), naringin (21), genistin (34), ellagic acid (42), leptosperine (46), chlorogenic acid (64) and neochlorogenic acid (69) were found to have relatively low Rf values in both solvent systems with Rf values ranging from 0.017 to 0.211 and 0.012 to 0.072 for MPA and MPB, respectively. This most likely reflects the presence of esters of sugar moieties or quinic acid (except for ellagic acid). The Rf values obtained using MPA were utilised in the development of databases 1A and 1B, while the Rf values obtained using MPB were used to establish databases 2A and 2B. To account for potential, slight, inter-run variations in the Rf values, the filtering threshold for the Rf values was set at ±0.05.

#### 2.1.2. Colour

The colour of the standards after development and derivatisation were determined as these were found to be very important tools in discriminating between different compounds. Colours of the samples analysed were determined as fluorescence after being irradiated with UV light at 254 nm after development, UV light at 366 nm after development and also after derivatisation, and with white light in transmittance mode after derivatisation. The VisionCat software of the HPTLC originally generated colours using the RGB colour space (see Table 1). These colours were in a next step converted into hue values for easier comparisons based on a single numerical value.

The hue values obtained at 254 nm after development ranged from 120° to 163.8°, thus from green (120.0° to 149.9°) to turquoise (150° to 179.9°) upon conversion into colour families with most of the standards presenting hue values at around 134° (green). Only four standards had hue values greater than 150°, which indicates little variation and thus discriminatory power of the colour at this imaging condition. The colours at 254 nm after development were obtained individually for each condition; they were, however, found to be very similar and therefore only one dataset is presented in Table 1.

The hue values obtained at 366 nm after development ranged from orange to violet (33.0° to 255.5°), with most standards having a hue value of around 180° (cyan blue). A greater variance in colour was observed at this imaging condition, and thus the obtained hue values at 366 nm after development were found to be helpful in discriminating between the different standards. Similar to the colours obtained at 254 nm after development, very similar values were recorded at 366 nm after development in all four conditions, and thus only one dataset is represented in Table 1.

The use of natural product reagent (also known as Naturstoff reagent or Neu’s reagent, diphenylborinic acid 2-aminoethyl ester (DPBA) and 2-aminoethyl diphenylborinate, CAS No. 524-95-8) is one of the most popular methods of investigating the fluorescence of flavonoids. It has been extensively used as a spray reagent for flavonoid detection in chromatography, exerting its activity through chelation or coordination/complexation, which can be captured as a green-to-yellow-to-orange fluorescence on excitation with UV or blue light [39]. Additional advantages for its use are minimal interference from other compounds [39], easy reagent preparation and convenient drying in warm air [40].

The hue values obtained at 366 nm after derivatising with NP-PEG reagent ranged from red to scarlet (24.3° to 346.6°), with most standards having a hue value of around 160° (turquoise). A great variation in colours was observed at this imaging condition and thus this derivatisation method was found to be very helpful in discriminating compounds from each other. Very similar hue values were observed when the standards were derivatised with NP-PEG reagent regardless of the solvent system used; therefore, only one dataset was used to develop databases 1A and 2A (Table 1).

Vanillin reagent is a very popular spraying reagent in HPTLC analysis. It is used to detect terpenoids, sterols, salicin, ergot, alkaloids and most lipophilic compounds [41].

Hue values obtained at 366 nm after derivatising with VS reagent ranged from 178.5° to 339.5° (turquoise to scarlet), with most compounds having hues of around 215° (blue). Flavonoids were observed to have hue values ranging from 178.5° to 230.6° (turquoise to blue), hydroxybenzoic acid and its derivatives to have hues ranging from 199.4° to 253.3° (cyan blue to violet), whereas hydroxycinnamic acid and its derivatives presented hue values between 201.9° and 245.2° (cyan blue to violet)

When analysed with white light in transmittance mode, the hue values obtained after derivatisation with VSA reagent ranged from 0.00° to 356.0° (red to scarlet), with most of the compounds having hue values at around 41° (orange). Flavonoids were found to have hue values ranging from 327° to 54.2° (red to orange), hydroxybenzoic acid and its derivatives from 340.0° to 111.1° (magenta to yellow green) and hydroxycinnamic acid and its derivatives from 9.5° to 136.1° (red to green).

Very similar hue values were observed when the standards were derivatised with VSA reagent regardless of the solvent system used; therefore, only one dataset was used to develop databases 1B and 2B (Table 1).

To account for the potential slight inter-run variations in colour prior to and also after derivatisation, the filtering threshold for hue values was set at ±60°.

#### 2.1.3. Fluorescence Spectra

The fluorescence spectra of the various standard compounds were obtained by scanning the standards from 190 nm to 390 nm. The spectra were carefully studied and the number of peaks as well as the respective λ max and λ min values were tabulated. Most standards had λ max values ranging between 220 and 270 nm.

λ max values obtained for repeat scans of each standard showed variations that were within ±2%; therefore, the λ max values for the different standards were considered as a reliable parameter in the establishment of all four databases (Table 1).

Similar to UV-Vis absorption behaviour, fluorescence is unique for each compound and can thus be used for the confirmation of chemical identity. For this study, the threshold for the database filtering was set at λ max ± 15 nm. Furthermore, the fluorescence spectra obtained for each standard were extracted as CSV files and used for spectral overlays in cases where the standard was considered as a potential candidate for matching with a band in the unknown.

#### 2.1.4. UV-Vis Spectra

The UV-Vis spectra of compounds were obtained by scanning the standards from 190 nm to 900 nm. However, given the potential interferences from the mobile-phase solvents used, only absorbances between 250 nm and 500 nm were taken into account prior to and also after derivatisation with NP-PEG reagent, whereas absorbances ranging from 250 nm to 600 nm were considered after derivatisation with VSA reagent. The number of peaks of the spectra were identified and the λ max of each peak tabulated.

Most standards (73) presented 1 peak, although 2 peaks could be identified for 30 standards, while only 4 standards had 3 peaks within the examined region. Almost all standards presented maxima between 251 and 393 nm. The λ max values obtained on repeat scans of each standard showed variations that were within ±2%; therefore, the use of λ max values of the standards was considered as a reliable parameter in the establishment of all four databases (Table 1).

UV-Vis absorption behaviour is directly related to the structure of each compound, and its λ max values can therefore be used as a filtering criterion in compound matching. The thresholds for database filtering for λ max values of the UV-Vis spectra were set at ±15 nm before derivatisation and ±60 nm after derivatisation. Furthermore, the spectra obtained for each standard were extracted as CSV files and used for spectral overlays in cases where the standard was considered a potential candidate for matching with a band in the unknown sample.

#### 2.1.5. Data Filtering

In order to try and match a band in the unknown sample with a standard in the database, the following filtering approach was adopted: the Rf value ±0.05 was set as the primary filtering parameter, followed by screening based on colour hue (±60°). The reduced list of potential candidates was filtered further using the fluorescence λ max (±15) and λ min (±15) values prior to derivatisation, λ max values (±15) of the UV-Vis spectrum prior to derivatisation, number of peaks of the UV-Vis spectrum prior to derivatisation, λ max values (±15) of the fluorescence spectrum after derivatisation and, finally, λ max values (±60) of the UV-Vis spectrum after derivatisation. The next step was the spectral overlays of the UV-Vis prior to and after derivatisation between potential matches and the unknown compound. A compound was considered a candidate match if it met all the criteria enumerated above, and if its spectral overlays showed a substantial similarity both qualitatively and quantitatively. The same approach was adopted for all four databases.

### 2.2. Validation of Databases Using Spiked Artificial Honey

Three test compounds were individually spiked into artificial honey to validate the filtering approach used in the application of the database. Test compounds A and C were hydroxybenzoic acid derivatives and test compound B a flavonoid. Both test compounds A and B were the standards in the database (positive control), whereas test compound C was not included (negative control).

The results of the database validation using these test compounds are detailed in Table 2, Table 3, Table 4, Table 5, Table 6, Table 7, Table 8, Table 9, Table 10, Table 11, Table 12, Table 13, Table 14 and Table 15. In all of these tables, the first row shows the Rf and hue of the respective test compound as well as the λmax and min of the fluorescence spectra prior to derivatisation, UV-Vis λmax prior to derivatisation, and fluorescence λmax and UV-Vis λmax after derivatisation with either of the two derivatising agents. The second row summarises the number of potential hits employing each filtering criterion, and the rows below list the potential hits and their respective data. As subsequent filtering criterions were applied, the number of potential hits reduced.

#### 2.2.1. Test Compound A

As demonstrated in Table 2, the Rf value of test compound A in MBA (databases 1A and 1B) is observed to be 0.608, and by applying the ±0.05 filtering criterion, 42 standards remain as potential candidates. The hue (H°) value of test compound A at 254 nm after development was 139.3°. By applying ± 60° as the filtering criterion, the number of potential matches in the database remained at 42. At 366 nm after development, the H° of test compound A was found to be 180°, which reduced the number of potential matches in the database to 40 based on the established H°± 60° filtering approach. After derivatisation with NP-PEG, the hue of test compound A was found to be 209.2°. By applying the ±60° filter criterion, the remaining number of potential matches in the database was 31. The fluorescence λ max of unknown A was found to be 225 nm. Using the ±15 nm filtering range, the number of matches remained at 31. However, when the λ min (at 258 nm) of the spectra was used and the filter applied, the number of potential matches was reduced to 25. The first UV-Vis λ max of test compound A was detected at 276 nm. Upon using the ±15 nm filtering range, the number of potential matches was narrowed to 11. As test compound A only had 1 λ max value, potential matches with a total of 2 or 3 maxima could be eliminated reducing the number of potential matches to 6. After derivatisation with NP-PEG, the fluorescence λ max of unknown A was found to be 239 nm, and by using the ± 15 nm filtering range the number of potential matches was reduced further to 5. When considering the UV-Vis λ max after derivatisation with NP-PEG (288 nm) and applying the screening criterion of ± 60 nm, the number of potential matches remained at 5. Thus, by applying the filtering approach outlined in Section 2.1.5, 5 compounds were considered as potential matches against database 1A, namely, 2,3,4-trihydroxybenzoic acid, eudesmic acid, methyl syringate, syringic acid and m-coumaric acid.

The same filtering methodology was then applied for the data generated for test compound A against database 1B. This reduced the initial set of potential matches from 33 to only 2 potential candidates, namely, methyl syringate and syringic acid (Table 3).

**Figure 1:**
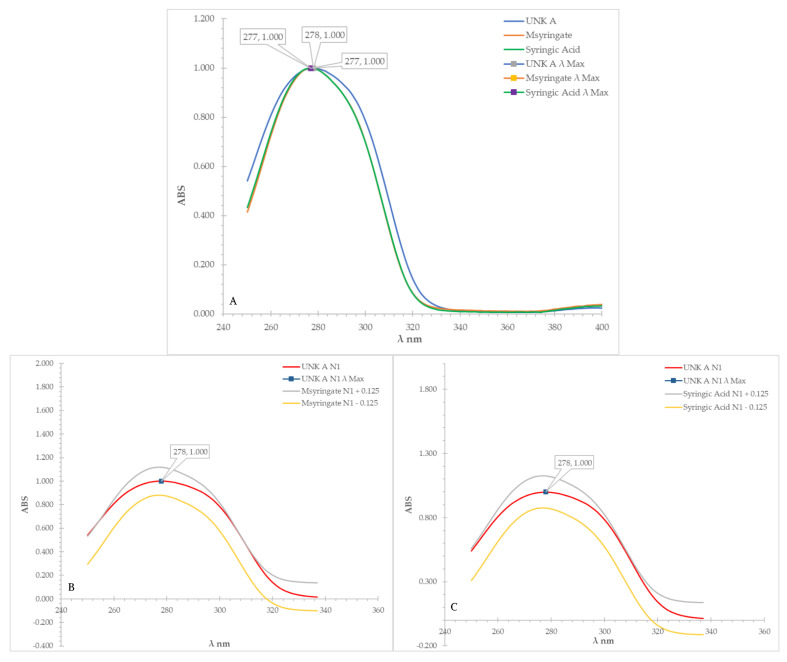
(**A**–**C**): UV-Vis (prior to derivatisation) spectra overlay of test compound A (UNK A) vs. methyl syringate and syringic acid (**A**), and UV-Vis (prior to derivatisation) spectra overlay of test compound A (UNK A) vs. the ±0.125 AU of methyl syringate (**B**) and vs. ±0.125 AU of syringic acid (**C**).

In the following step, spectral overlays were performed with the UV-Vis spectrum of test compound A and that of methyl syringate and sringic acid, respectively (Figure 1A). Test-compound A featured an absorbance peak between 250 and 337 nm, and when overlaying and comparing this region with methyl syringate, a correlation of 0.993 was observed. For the syringic acid, the correlation was 0.994; thus, by applying the difference threshold of ±0.100, no discrimination between the quality of the match could be achieved. Similarly, the spectral overlay based on a ±0.125 AU range of the spectra of each match resulted in 95.5% of the absorbance values of the test compound in the investigated region to fall within that of the methyl syringate (Figure 1B), while the match was 100.0% for the syringic acid (Figure 1C). These findings were not outside the ±10% difference threshold set for matching, indicating that the UV-Vis spectral matching prior to derivatisation was insufficient for identifying the identity of the test compound.

**Figure 2:**
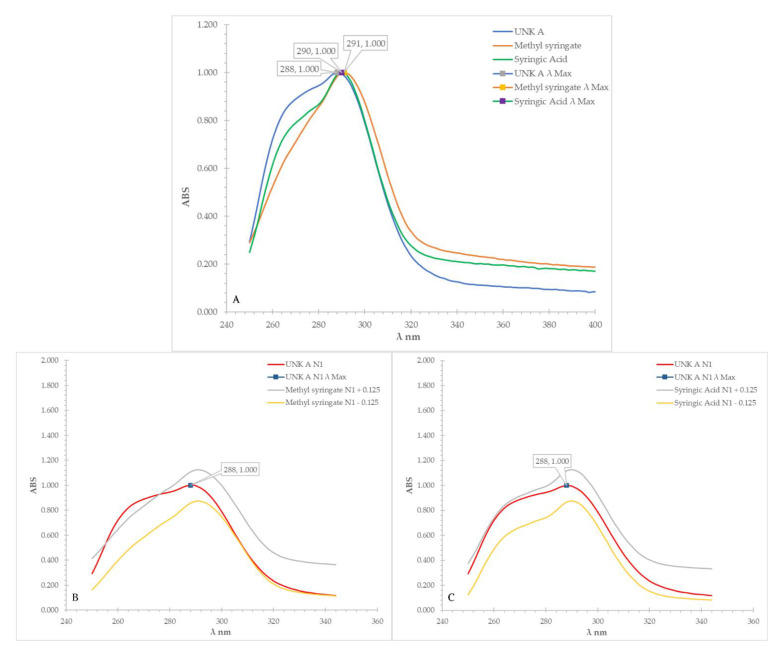
(**A**–**C**): UV-Vis (after derivatised in NP-PEG reagent) spectra overlay of test compound A (UNK A) vs. methyl syringate and syringic acid and UV-Vis (after derivatised in NP-PEG reagent) spectra overlay of test compound A (UNK A) vs. ±0.125 AU of methyl syringate (**B**) and vs. ±0.125 AU of syringic acid (**C**).

The same spectral overlay approach was applied to the UV-Vis spectra obtained after derivatisation with NP-PEG reagent. Figure 2A shows the UV-Vis spectral overlay versus the consolidated matches. Test compound A was found to have a UV-Vis peak between 250 and 344 nm; the correlation in this region with methyl syringate was found to be 0.939, while for syringic acid it was 0.986, again an insufficient difference to discriminate between the two standards based on a ±0.100 threshold difference. On visual inspection, a distinct shoulder in the spectrum of test compound A could be observed, a feature that is also present in the spectrum of syringic acid (Figure 2C), but not in that of methyl syringate (Figure 2B). When quantitatively analysed, only 78.9% of the absorbance values of test compound A fell within the ±0.125 AU of the signals of methyl syringate (Figure 2B), whereas 100.0% of its absorbance values were found to be within the ±0.125 AU of the signals of syringic acid (Figure 2C). Applying the ±10% difference threshold, it could thus be concluded that test compound A was most likely syringic acid, indicating that UV-Vis analysis after derivatisation with NP-PEG reagent was able to discriminate between the two remaining consolidated candidates.

**Figure 3:**
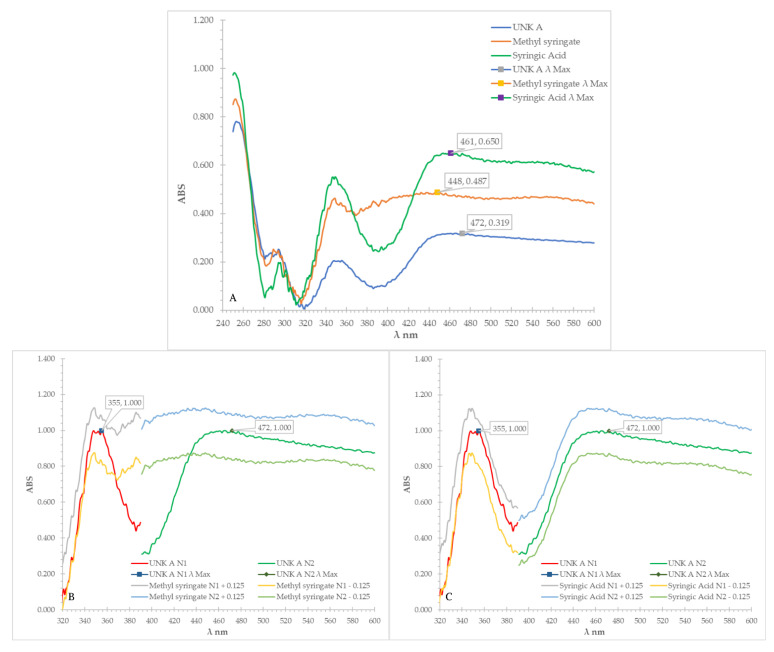
(**A**–**C**): UV-Vis (after derivatised in VSA reagent) spectra overlay of test compound A (UNK A) vs. methyl syringate and syringic acid and UV-Vis (after derivatised in VSA reagent) spectra overlay of test compound A (UNK A) vs. the ±0.125 AU of methyl syringate (**B**) and vs. ±0.125 AU of syringic acid (**C**).

Figure 3A shows the UV-Vis spectral overlay of test compound A after derivatisation with VSA reagent against the two consolidated candidate matches with methyl syringate yielding a matching correlation of 0.669, whereas that of syringic acid was 0.870. By applying the difference threshold of ±0.100, it was found that test compound A had a spectral signature that was more similar to that of syringic acid. Additionally, by analysing the spectral overlays visually (Figure 3B,C), the wave inflections of test compound A were more like syringic acid when compared to methyl syringate. Quantitatively, only 60.5% of the absorbance values of test compound A fell within the ±0.125 AU of the signals of methyl syringate (Figure 3B), whereas the match was found to be 84.3% for syringic acid (Figure 3C). By applying the ±10% difference threshold, it could thus be concluded that test compound A was more likely to be syringic acid, indicating that the UV-Vis analysis after derivatisation with VSA reagent was also able to discriminate between the two consolidated potential matches.

The fluorescence spectra of test compound A prior to and after derivatisation were also compared with the two potential matches, but they did not allow us to discriminate between them and therefore did not add any further information to the analysis.

The same filtering procedure as that described above in detail was followed for test compound A using data generated in MPB. Table 4 and Table 5 illustrate how a list of potential matches was derived using derivatisation with NP-PEG (database 2A) or VS (database 2B), respectively. As can be seen, the database matching yields syringic acid as the sole match for test compound A, confirming the previous results. Post-analysis, the correct identification of test compound A was confirmed, validating the filtering process established for the database application.

#### 2.2.2. Test Compound B

The same filtering approach as that described in Section 2.2.1 was conducted in order to ascertain the identity of test compound B. Using the data generated with MPA (Table 6 and Table 7), isorhamnetin and kaempferol were identified as potential hits and were therefore considered as the consolidated matches for test compound B.

Corresponding data, generated using MPB, are shown in Table 8 and Table 9. Isorhamnetin and kaempferol were once more identified as potential matches in this screening process.

In order to identify the correct match, various spectral overlays were performed (Figures S16–S18). The Pearson’s correlations as well as percent matches for each pair of databases are shown in Table 10.

Based on the derived correlations and percentage spectral matches, kaempferol was considered as a match for test compound B. A post-analysis of the correct identification of test compound B was confirmed, again validating the filtering process established for the database application.

#### 2.2.3. Test Compound C

Test compound C served as a negative control to ensure that the database filtering approach and spectral overlay system did not yield false-positive hits. The data obtained following the various filtering steps are presented in Table 11 and Table 12 using MPA and Table 13 and Table 14 using MPB.

Table 15 illustrates that hesperetin, naringenin, benzoic acid, m-toluic acid, m-coumaric acid and p-methoxyphenyllactic acid are all identified as potential hits for test compound C and are therefore considered as consolidated matches, and based on the spectral overlays (Figures S19–S20), none of the standards in the database could be identified as matches for test compound C as only low correlations and percent spectral matches could be found (Table 10).

Post-analysis, test compound C was confirmed to be acetyl salicylic acid or aspirin (CAS No. 50-78-2, Chem Supply Australia Pty Ltd. (Port Adelaide, SA, Australia), a standard that was not included in the database, confirming the ability of the screening process to successfully avoid false-positive identifications.

#### 2.2.4. HPTLC Analysis of Manuka Honey Extract

**Figure 4:**
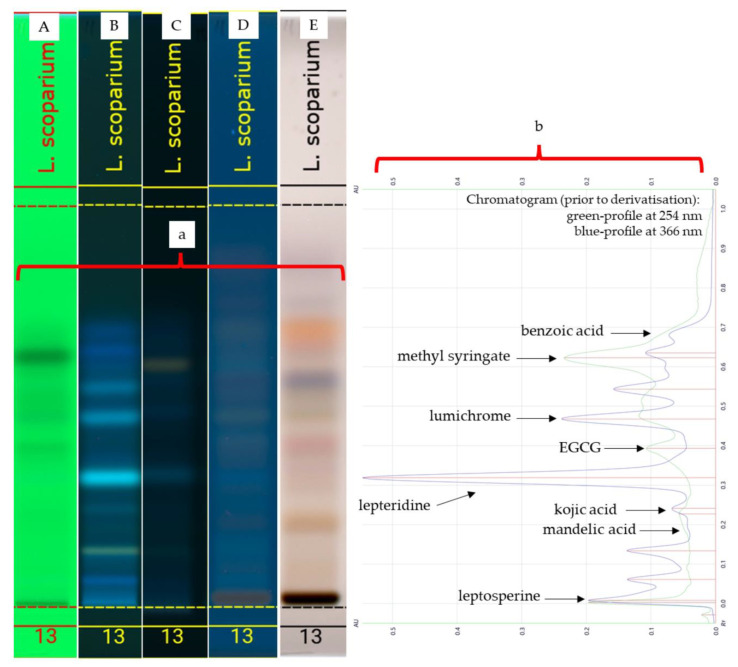
HPTLC plate images (**a**) obtained under the following light conditions: 254 nm (prior to derivatisation A), 366 nm (prior to derivatisation B), 366 nm (after derivatised with NP-PEG-C), 366 nm (after derivatisation with VSA), transmittance in white light (after derivatisation with VSA-E) and chromatograms (**b**) of Manuka honey (*L. scoparium*) using mobile-phase A.

**Figure 5:**
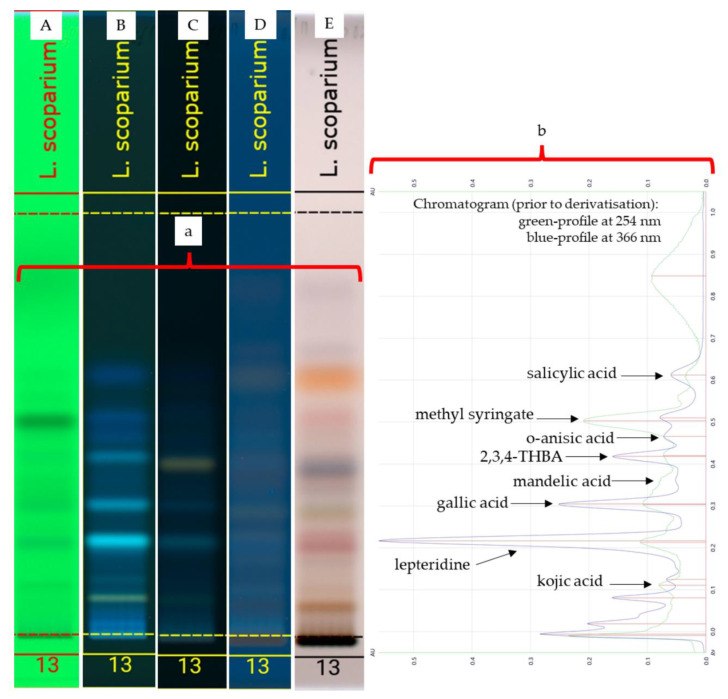
HPTLC plate images (**a**) obtained under the following light conditions: 254 nm (prior to derivatisation A), 366 nm (prior to derivatisation B), 366 nm (after being derivatised with NP-PEG-C), 366 nm (after being derivatised with VSA-D), transmittance in white light (after being derivatised with VSA-E) and chromatograms (**b**) of Manuka honey (*L. scoparium*) using mobile-phase B.

The organic extract of Manuka honey (*Leptospermum scoparium*) was analysed using HPTLC, and images were generated in the same way as for the standards and the artificial honey spiked with the three test compounds (Figure 4a and Figure 5a). Images taken prior to derivatisation (A and B) were converted into corresponding chromatograms (Figure 4b and Figure 5b) to determine major peaks and served as Rf references for the spectral scans that were performed.

The Rf values, colour hues and λ max and λ min for the UV-Vis and fluorescence spectra obtained prior to and after derivatisation with the NP-PEG and VS reagents were tabulated for all the identified major bands in Manuka honey (Tables S15 and S16 for MPA and Tables S17 and S18 for MPB). The validated filtering process as outlined before was employed to determine the potential matches for these bands.

After identifying the consolidated matches, spectral overlays were performed in a similar way as described in previous sections. A summary of the correlations and percent matches is presented in Table 16.

In summary, by using databases 1A and B, the band at Rf 0.02 was identified as leptosperine (46, see Table S8 and Figure S8 for structure), the band at Rf 0.199 as mandelic acid (80, see Table S10 and Figure S11 for structure) and the band at Rf 0.240 as kojic acid (105, see Figure S15 for structure). The band at Rf 0.319 was identified as lepteridine (106, see Figure S15 for structure), Rf 0.392 as epigallocatechin gallate (EGCG), (29, see Table S5 and Figure S5 for structure), Rf 0.460 as lumichrome (107, see Figure S15 for structure) and the band at Rf 0.623 as methyl syringate (48, see Table S8 and Figure S8 for structure).

By using databases 2A and 2B, the band at Rf 0.150 was identified as kojic acid, the band at Rf 0.220 as lepteridine, the band at Rf 0.310 as gallic acid (44, see Table S8 and Figure S8 for structure) and the band at Rf 0.349 as mandelic acid. The band at Rf 0.425 was identified as 2,3,4-trihydroxybenzoic acid (36, see Table S8 and Figure S8 for structure), the band at 0.470 as o-anisic acid (52, see Table S8 and Figure S8 for structure), the band at Rf 0.513 as methyl syringate and, finally, the band at Rf 0.603 as salicylic acid.

Ideally, the matches identified in one database set (DBs 1A and 1B) should also be identified in the second database set (DBs 2A and 2B). This ‘double identification’ was possible for kojic acid, lepteridine, methyl syringate and lumichrome. However, given the complexity of honey as a natural product and the presence of a multitude of compounds in the investigated honey extract, well separated and thus potentially identifiable bands in one solvent system might overlap with bands in another solvent system, with a poor band resolution making compound identification impossible. In this light, it is plausible that some matches were only found in one but not the second database set. In the case of Manuka honey, this was the case for leptosperine, mandelic acid, epigallocatechin gallate, lumichrome, 2,3,4-trihydroxybenzoic acid, o-anisic acid and salicylic acid. The richness of the data generated by HPTLC analysis and explored in the methodological compound-identification approach outlined in this study thus offered various avenues to successfully match compounds against the established databases.

Lepteridine has been previously reported in Manuka honey and is considered one of the honey’s biomarkers [26,27,35,42]. Gallic acid was also found to be present in Manuka honey, which was previously reported [21,24,26,31,33,43], along with methyl syringate [25,26,28,29,31,33,35,44], lumichrome [26,29,45], leptosperine [25,26,27,35], salicylic acid [29,46], o-anisic acid [19,26,27,29,33,44], 2,3,4-trihydroxybenzoic acid [21] and kojic acid [44]. This study was, however, the first to report the presence of mandelic acid and epigallocatechin in Manuka honey.

#### 2.2.5. Validation by HPLC

HPLC with a photodiode array detector (DAD or PDA) [20,21,25,26,27,29,34,42,47,48], and UV or UV/UV detector [19,22,24,28,49] were found to be the most commonly used instrumentations in the identification of phenolic compounds in honey, followed by LC-MS [31,33,46]. A combination of HPLC, LC-MS and GC-MS [44], and also fluorescence spectroscopy, were used in the study [33]. Given the popularity of HPLC with photodiode array detection for determining phenolic constituents in honey, a cross-validation of the findings of this study with the data obtained with this instrumentation was performed.

The validation was conducted at the University of the Sunshine Coast Honey Research Laboratory using HPLC DAD (protocol not published). Based on this analysis, Leptosperin was detected as the most abundant compound (522 ppm), followed by 3-phenyllactic acid or DL-β-phenyllactic acid (226 ppm), methyl syringate (60 ppm), 2-methoxybenzoic acid or o-anisic acid (54 ppm), kojic acid (34 ppm), pyrogallol (13 ppm), lepteridine (8 ppm), p-hydroxyphenyllactic acid (5 ppm), syringic acid (3 ppm) and lumichrome (3 ppm).

Of these identified Manuka honey constituents, 3-phenyllactic acid or DL-β-phenyllactic acid, pyrogallol, p-hydroxyphenyllactic acid and syringic acid were not detected by HPTLC, although these standards were part of the database used for filtering. On the other hand, salicylic acid, 2,3,4-THBA, mandelic acid and EGCG were not detected by HPLC-DAD, but could be identified in the HPTLC analysis. These differences might be attributed to the fact that for the HPLC analysis, the honey sample in its entirety was analysed as an aqueous solution, whereas in the case of the HPTLC analysis, an organic extract of the honey was utilised. The solvent used in the extraction of the non-sugar constituents in honey was dichloromethane: acetonitrile (1:1) that might not have extracted all non-sugar constituents present. In a study conducted by Stanek and Jasicka-Misiak [20], acidified Manuka honey (pH 2 with HCl) was extracted using Amberlite XAD-2 in order to obtain its phenolic constituents. From this extract, rosmarinic acid, ellagic acid, p-coumaric acid and myricetin were qualitatively determined based on colour and Rf values, indicating that the extraction method can play a significant role in the type of compounds that are detected in the analysis.

## 3. Discussion

The proposed HPTLC-based methodology offered advantages over other compound identification strategies. Two detection conditions, namely, excitation at 254 nm and at 366 nm, are commonly used in HPTLC prior to compound derivatisation. Phenolics can be detected by quenching the adsorbent material’s fluorescence (254 nm) and/or after UV irradiation (366 nm), with the latter being considered the most sensitive detection method in HPTLC. Detection modes are in the form of images from which rich data can be derived, particularly the colour and Rf values of the bands.

The colours of bands in HPTLC analysis have been used in the qualitative and quantitative analyses of honey; however, they were only described using basic colour descriptions [20,50,51]. This study was able to capture more nuanced colour differences in the bands by converting their RGB values into a single hue (H°) value. This conversion allowed the RGB values to be easily captured, expressed by a single value that facilitated inter-band colour comparisons. It was also observed that certain groups of compounds or compounds that contained the same functional groups tended to present distinct, bright-colour or fluoresce patterns that might be used to predict their compound class. For example, flavones, such as acacetin (3), apigenin (4), chrysin (6), gekwanin (7) and vitexin (9), have one active site located at the 5-hydroxy-4-keto group, and they all had hue values ranging from 61.5° to 121.6° (yellow to green).

Since these imaging conditions without derivatisation might, nonetheless, not capture all compounds, those that are not detectable at 254 or 366 nm can be visualised after a reaction with a suitable reagent; in this study, either VSA or NP-PEG [52]. In HPTLC-based honey research, to date, 1% methanolic AlCl_3_, ceric phosphomolybdate, 2-aminoethyl diphenylborate, vanillin sulfuric acid and 2,2-diphenyl-1-picrylhydrazyl (DPPH) appear to be the most popular spraying reagents [20,37,50,51,52,53]. In this study, 2-aminoethyl diphenylborate (natural product reagent or Neu’s reagent) and vanillin sulfuric acid were used to derivatise the honey constituents for visualisation. The derivatisation did not only enhance the visualisation of the samples, but also enhanced their UV-Vis and fluorescence spectra. Since compounds might react differently to different spraying reagents, using two derivatising reagents increased the chances of enhanced visualisation and ultimately compound matching. The rich data generated in the RGB and subsequent hue values of the various honey bands at 254 nm, and 366 nm as well as at 366 nm and white light after derivatisation with VS and NP-PEG reagent thus allowed for higher levels of certainty in the compound identification compared to the analytical approaches (e.g., HPLC-DAD) that were unable to tap into this rich resource of information for screening and filtering purposes to discriminate between structurally often very similar honey constituents.

The study also used two different mobile phases to ensure that a large number of extracted honey bands could be adequately accounted for. Using MPA, six compounds could be identified in Manuka honey; with the slightly less polar MPB, the chemical identities of eight compounds could be determined.

Furthermore, an additional advantage of HPTLC-derived Rf values as primary screening criterion compared to other identification techniques relying on chromatographic separation of compounds (e.g., HPLC) is that no Rf drift over time associated, for example, with an aging of column material can be encountered. Rf values in the database are therefore more stable and are able to be used for long-term screening studies, as in every experiment a fresh stationary phase will be used and therefore changes over time in retention factors will not occur.

In this study, the fluorescence λ max and λ min after development, UV-Vis λ max after development and after derivatisation, and the fluorescence λ max after derivatisation were all also been found to be important tools in narrowing the number of possible matches for an unknown sample. A small tolerance range (±15 nm) was employed as a filtering criterion, except for UV-Vis spectra after derivatisation (±60 nm), in order to take into consideration a possible change in pH and other parameters, which can lead to slight bathochromic or hypsochromic shifts. Although the use of HPTLC is not new in the analysis of phenolic compounds in honey [5,8,20,50,52,54,55], to our knowledge, this study was the first to report the use of a HPTLC scanner for honey-compound determination. It was found that UV-Vis spectra after development alone could not always univocally determine the identity of a compound; therefore, UV-Vis spectra after derivatisation were also utilised. These, especially after derivatisation with NP-PEG reagent, were found to be a very important discriminating tool in determining the unknown sample’s identity. Compared with structure determination methods that solely rely on UV-Vis identification (e.g., HPLC-DAD), HPLTC-based screening can thus offer a distinct advantage.

The “gold standard” for phenolic compound determination is ultra-high-performance liquid chromatography coupled with high-resolution mass spectrometry (UHPLC-HRMS) [56]. However, it is a costly technique, both in terms of equipment and running costs, and it requires technical expertise for its operation and data analysis [57]. In light of these shortcomings, high-performance liquid chromatography (HPLC) coupled with diode array detection (DAD) appears to be the most commonly employed technique for the qualitative and quantitative analyses of phenolic compounds [1,8,16,58]. Although the set up of HPLC–DAD is relatively cheap and the analysis robust, the method nonetheless faces several disadvantages, such as i) compound identification is only based on retention times and associated UV spectra, which might not allow us to sufficiently discriminate between compounds of a very similar chemical nature, and ii) low detection and quantification limits when analysing complex matrices [59].

The spectral overlay system that was developed, along with the use of correlation and percent-match calculations of the inflection of the unknown against potential compound matches, was also found to be a very useful tool not only in differentiating between multiple potential matches, but also in confirming the identity of the unknown ones. While studies employing DA detection might also be able to tap into spectral characteristics beyond simple λ max information, the availability of multiple spectra (UV-Vis and fluorescence spectra prior and after derivatisation) offers much richer data and therefore a better chance of univocally discriminating between potential matches of similar chemical characteristics that might not be able to be differentiated using a single UV-Vis spectrum. A case in point is unknown A where, based on the spectral overlay of the UV-Vis spectrum prior to derivatisation, a distinction between the two candidate matches of methyl syringate and syringic acid was not possible; however, on closer inspection of their UV-Vis spectra after derivatisation with NP-PEG reagent, it could be determined that syringic acid was the correct match.

While compound matching using the database and the developed filtering approach is a multi-step process, many of the individual steps can be automated, for example, the generation of hue values, spectral overlays (using pre-modelled Excel^®^ worksheets) as well as the calculation of correlations and % agreement in absorbance values.

Despite the significant advantages over other compound identification methods, some limitations of the HPTLC-derived database system for the qualitative analysis of honey constituents need to be acknowledged. The approach relies on an extensive set of data, which requires a considerable amount of time to acquire, particularly the four scanning steps and the manual entry of RGB values.

It is also important to note that the derivatising process, particularly when using VSA reagent, is highly time dependent and must therefore be conducted in a controlled manner to generate reproducible results. The HPTLC development itself is also dependent on moisture levels, which can affect the Rf value of the bands. As the Rf value is used as primary screening criterion, the development of the plate should thus be performed in a humidity-control development chamber. To address this potential limitation, Rf ±0.05 was set as the primary filtering criterion in the database search to allow for slight run to run variations.

Another limitation of the use of HPTLC in the analysis of phenolic compounds in honey is that, by default, the photodocumentation is restricted to the use of only two wavelengths, namely, 254 nm and 366 nm. This means that prior to derivatisation, only those compounds that absorbs at these wavelengths (254 nm for most simple phenolic compounds and 366 nm for most flavonoids) will be detected. Most caffeic acid derivatives, for example, present λmax values of around 320 nm; therefore, this compound class is difficult to identify using default detection. However, with the use of two derivatisation reagents, it was possible to overcome this limitation.

The development of the database used in this study can easily be adopted in another laboratories, as long as the HPTLC instrumentation and conditions indicated in the Methodology Section are properly replicated.

The database has at this point only been developed for qualitative analysis, and thus the limit of detection has not yet been determined; the system has, however, been validated for a reliable identification of phenolic compounds in honey and has been demonstrated to be a powerful tool preceding potential quantification experiments.

## 4. Materials and Methods

### 4.1. Chemicals and Reagents

In general, chemical standards were purchased from Ajax Finechem Pvt. Ltd. (Sydney, Australia), AK Scientific, Inc. (Union City, CA, USA), Alfa Aesar (Lancashire, UK), Angene International Ltd. (Nanjing, China), Chem Supply Australia Pty Ltd. (Port Adelaide, SA, Australia), Combi-Blocks Inc. (San Diego, CA, USA), Wuhan ChemFaces Biochemical Co., Ltd. (Wuhan, Hubei, China), Sigma Aldrich (Castle Hill, NSW, Australia) and Sigma-Aldrich (St. Louis, MO, USA). Figures S1–S15 and Tables S1–S14 summarise information, such as the identity, supplier, prepared concentration and HPTLC sample application of the standard compounds used to construct the database. Compounds were selected based on the findings of a comprehensive review of (mainly) phenolic compounds identified, to date, in honeys around the world [16]. The pool of standards was complemented with isomers of some of these compounds, compounds that were reported for other bee products (e.g., pollen and propolis) and other phenolic compounds available to the research team. The standards were grouped in line with common phenolic compound classifications [16,60,61,62].

Other reagents used in the study were purchased from the following suppliers: Anhydrous magnesium sulphate (7487-88-9), ethanol (64-17-5), 2-aminoethyl diphenylborinate (524-95-8), glucose (D-glucose anhydrous, 50-99-7) and sucrose (57-50-1) from Chem Supply Australia Pty Ltd. (Port Adelaide, SA, Australia); vanillin (121-33-5) from Sigma-Aldrich (St. Louis, MO, USA); naringenin (67604-48-2) from Alfa Aesar (Lancashire, UK); ethyl acetate (141-78-6) and formic acid (64-18-6) from Ajax Finechem Pvt. Ltd. (Sydney, Australia); fructose (57-48-7) and maltose (6363-53-7) were purchased from Sigma Aldrich (Truganina, VIC, Australia); toluene from APS Chemicals (Sydney, Australia); dichloromethane (75-09-2), acetonitrile (75-05-8) and HPTLC Silica gel 60 F254 Plates 10 × 20 cm from Merck KGaA (Darmstadt, Germany); methanol (67-56-1) from Scharlau (Barcelona, Spain); PEG (25322-68-3) from PharmAust Manufacturing (Welshpool, Western Australia) and sulfuric acid (7664-93-9) from Merck KGaA (Darmstadt, Germany).

### 4.2. Honey Sample

Manuka honey (*Leptospermum scoparium*) was purchased from a local honey supplier in Queensland. No further authentication was performed to confirm its floral origin.

### 4.3. Preparation of Standards and Reagents

#### 4.3.1. Standards

All standards used in the study were dissolved in methanol to concentrations as indicated in Tables S1–S14. Naringenin (0.5 mg/mL in methanol) was used as the HPTLC reference standard.

#### 4.3.2. Developing Solvent

Two different mobile phases were used in the study: (a) toluene:ethyl acetate:formic acid (2:8:1, *v/v/v*), referred to as MPA, and (b) toluene:ethyl acetate:formic acid (6:5:1, *v/v/v*) referred to as MPB.

#### 4.3.3. Derivatising Reagents

Natural product (NP)-derivatising reagent was prepared by dissolving 1 g of 2-aminoethyl diphenylborinate in methanol and then the volume of the solution was made up to 100 mL (1% m/v) [63]. Polyethylene glycol (PEG-400) reagent was prepared by mixing 5 g of polyethylene glycol in ethanol and then the volume of the solution was made up to 100 mL (5% m/v) [63]. Vanillin sulfuric acid (VSA)-derivatising reagent was prepared by adding 2 mL of sulfuric acid to 100 mL 1% vanillin solution (1 g/100 mL in ethanol) [36,52,64]. All derivatising reagents were stored at 0 °C when not in use.

#### 4.3.4. Artificial Honey

An artificial honey solution was prepared by diluting 2 g of a sugar stock solution (21.625 g of fructose, 18.125 g of glucose, 1.000 g of maltose, 0.750 g of sucrose and 8.500 g of water) to 5 mL (40%) with deionised water. The solution was stored at 0 °C and used within a week [65].

### 4.4. Extraction of Non-Sugar Components in Honey

Each honey (1 g) was mixed with 2 mL of deionised water in stoppered glass test tubes and vortexed to facilitate dissolution and mixing. The resulting aqueous honey solutions were then extracted 3 times with 5 mL of a mixture of dichloromethane and acetonitrile (1:1, *v/v*). The respective organic extracts were combined and dried with anhydrous MgSO_4_, filtered and then evaporated to dryness at 35 °C. The organic honey extracts were stored at 4 °C until analysis for which they were reconstituted with 100 µL methanol.

Artificial honey solutions containing 1% (m/v) of standards A and B, which were included in the database, were prepared as positive controls, and another artificial honey solution containing 1% (m/v) of standard C, which was not in the database, served as a negative control in the validation of the filtering approach used in the database application.

### 4.5. Chromatography and Derivatisation

Each standard and the respective honey/artificial honey extracts were chromatographed and derivatised as follows: duplicate plates were developed in mobile-phase MPA and were derivatised using either NP-PEG or VSA reagents (for details see Section 4.5.3). Duplicate plates were also run using mobile-phase MPB and again derivatised with NP-PEG and VSA, respectively. MPA was selected as a mobile phase because previous studies of honey using HPTLC analysis employed this mobile phase, allowing for cross-references to previous studies. MPB, with a slightly higher polarity, was chosen to ensure that more polar phenolics were also adequately separated and detected. In a similar way, VSA was employed as a derivatisation reagent as it had been used in numerous, previous HPTLC-based honey analyses [36,37,66], allowing for cross-referencing, whereas NP-PEG was selected as a versatile derivatisation reagent particularly suitable for the identification of phenolic compounds [67], which also allowed us to broadly differentiate between flavonoids and other phenolics based on colour development [39,67].

#### 4.5.1. Sample Application

For the naringenin reference standard, 4 µL was applied, and for honey and artificial honey extracts, 7 µL. The application volumes for the various standards varied (Tables S1–S14). All samples were applied as 8 mm bands, 8 mm from the bottom of the HPTLC plate at a rate of 150 nL s^−1^ using a semi-automated HPTLC sample applicator (Linomat 5, CAMAG).

#### 4.5.2. Plate Development

The chromatographic separation was performed on HPTLC plates (20 cm × 10 cm glass-backed silica gel 60 F_254_ plates) in an activated (MgCl_2_·6 H_2_O, 33–38% relative humidity) automated twin-trough (20 × 10 cm) development chamber (ADC2, CAMAG). The system was saturated for 15 min using saturation pads and the plates were first preconditioned with the mobile phase for 5 min, automatically developed to a distance of 70 mm at room temperature and then dried for 5 min. The developed plates were then photo-documented using the HPTLC imaging device (TLC Visualizer 2, CAMAG) under 254 nm, 366 nm and white light in transmittance mode (T). The entire chromatographic process as well as digital image processing and analyses were controlled by specialised HPTLC software (VisionCATS 3.1, CAMAG).

#### 4.5.3. Derivatisation

To derivatise samples using NP-PEG reagent, the plates were first sprayed with 3 mL of 1% NP reagent (CAMAG Derivatiser, green nozzle at level 3) and were then allowed to dry for 5 min at 40 °C (CAMAG TLC Plate Heater III). The plates were then derivatised again using 5% PEG reagent (blue nozzle at level 2) followed by drying (CAMAG TLC Plate Heater III) for 5 min at 40 °C. The derivatised plates were analysed at 366 nm.

To derivatise plates using VSA reagent, they were sprayed with 3 mL of 1% vanillin sulphuric acid reagent (CAMAG Derivatiser, yellow nozzle at level 3), then heated (CAMAG TLC Plate Heater III) at 115 °C for 3 min. The derivatised plates were analysed at 366 nm and T white light after cooling down for 2 min.

#### 4.5.4. Scanning of Individual Bands

Chromatograms of all standards and samples were generated in the various conditions and the major peaks were automatically determined by the software. A TLC Scanner 3 (CAMAG, Muttenz, Switzerland) was used to scan the spectra of each standard and the individual, to identify significant bands in both the UV-Vis (190–900 nm) and fluorescence excitation (190–380 nm) modes. The scans were performed using the following settings: dimension 5 × 0.2 mm (micro), optimisation set for maximum resolution, scanning speed set at 20 nm/s and K400 optical filter. Deuterium (190-380 nm) and tungsten (380-900 nm) were used as lamps and the fluorescence excitation mode was set at 380</400 nm scans, and the emission was observed at 190–270 nm. Scanning was performed twice, prior and after derivatisation.

#### 4.5.5. Systems Suitability Test (SST)

As a quality control step, a system suitability test (SST) was built into the analysis of all plates used both in the database development and also the qualitative determination of phenolic compounds. Only the results from plates that passed the set threshold of ± 0.05 for the Rf and the minimum height for MPA (Rf 0.690, minimum height 0.108) and MPB (Rf 0.550, minimum height 0.120) were used.

### 4.6. Data Tabulation

Rf values in the two mobile phases used colours (expressed as RGB values) in the various image conditions (254 nm, 366 nm and T white light prior to derivatisation and 366 nm and T white light after derivatisation with NP-PEG and VSA reagent), UV-Vis and fluorescence spectra prior to and after derivatisation were recorded for all standards as well as the honey/artificial honey extracts.

In addition, colours expressed as RGB values were converted into hue values using the following formula:

HUE (°) = IF(180/PI()*ATAN2(2*A3 − B3 − C3,SQRT(3)*(B3 − C3))<0,180/PI()*ATAN2(2*A3 − B3 − C3,SQRT(3)*(B3 − C3))+360,180/PI()*ATAN2(2*A3 − B3 − C3,SQRT(3)*(B3 − C3)))(1)

where A = Red value, B = Green value and C = Blue value (https://www.mrexcel.com/board/threads/rgb-to-hue-formula.559852/, accessed on 30 September 2022).

Hue values were included in the database in lieu of RGB values as it allowed us to express colour by a single number. These hue values were further aligned with the colour family based on the following conversions: 0.00–29.99 = Red (R), 30.00–59.99 = Orange (O), 60.00–89.99 = Yellow (Y), 90.00 –119.99 = Yellow Green (YG), 120.00–149.99 = Green (G), 150.00–179.99 = Turquoise (T), 180.00–209.99 = Cyan Blue (CB), 210.00–239.99 = Blue (B), 240.00–269.99 = Violet (V), 270.00–299.99 = Purple (P), 300.00–329.99 = Magenta (M), 330.00–359.99 = Scarlet (S) (https://www.blog.jimdoty.com/?p=11507, accessed on 30 September 2022).

### 4.7. Database Establishment

Four sub-databases were established (DB 1A and 1B, and 2A and 2B), one for each solvent system as well as the respective derivatisation agent used in the analysis.

### 4.8. Database Search Strategies

**Figure 6:**
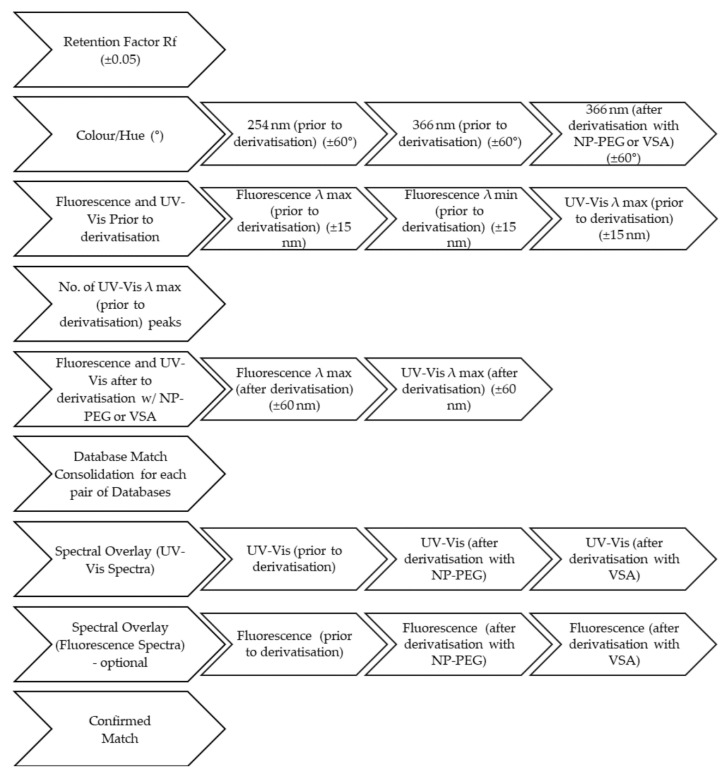
Database filtration and spectra overlay protocol for determining the identity of an unknown sample using the developed HPTLC-based database.

To match the unknown honey bands with potential standards included in the database for compound identification, a comprehensive strategy was set in place (Figure 6). A sort and filter feature was formulated so that the database automatically returned potential match compounds based on the information provided for the unknown.

*Rf value.* Rf values of unknown bands ± 0.05 were used as primary search criterion to generate a first list of potential hits from the database.

*Hue values.* Hue values were then used to narrow down further the list of potential hits using the hue value of the unknown band ± 60 as additional filter criterion.

*Fluorescence λ max and λ min, and UV-Vis λ max.* In a next step, we tried to match the fluorescence λ max and λ min, and the UV-Vis λ max of unknown honey bands prior to derivatisation, and the number of λ max/peaks in the UV-Vis spectra prior to derivatisation with that of any of the remaining potential hits in the database using λ ± 15 nm as the filtering criterion. This was followed by a potential matching with fluorescence λ max after derivatisation with λ ± 15 nm as the filtering criterion, and finally, with the UV-Vis λ max after derivatisation with λ ± 60 nm as filtering criterion.

To perform spectral matching with the identified potential hits, an automated spectral overlay system (see Section 4.9) was developed using Excel^®^, where spectral regions around the respective λ values of the unknown honey band (+/- 15 nm) were examined and the spectra considered to be a close match with a standard if the unknown band’s normalised absorbance fell within +/- 0.125 of the normalised absorbance of the standard identified as a potential hit.

If at this stage a final identification could not be performed because more than one standard in the database met the above search criteria, the respective fluorescence spectra prior to as well as after derivatisation with VS and NP-PEG were also investigated for potential matches adopting the screening process as described above.

### 4.9. Spectral Overlay System using Excel

Although the maxima of fluorescence and UV-Vis absorbance prior to and after derivatisation, and the minima of fluorescence prior to derivatisation were used to determine the identity of the unknown bands, these were found to be insufficient to confirm the identity of compounds in all instances. An Excel^®^-based spectral overlay system was therefore developed. It determined how closely matched the spectral features of the unknown around the respective λ value(s) were to those observed in potential matches identified in the sequence of prior database filtering steps. Comparisons were based on normalised spectra where the absorbance at the λ max of either the unknown or standard was set to 1 to facilitate the comparison of the inflections of the waves. The normalisation was performed using the following formula:

(2) $$\mathrm{Normalised}\mathrm{ABS}\mathrm{of}\mathrm{standard}/\mathrm{unknown}=\mathrm{ABS}\mathrm{of}\mathrm{standard}/\mathrm{unknown}*(\frac{1}{\mathrm{ABS}\mathrm{of}\lambda \max})$$

where ABS—absorbance of either the standard or the unknown, *ABS* of λmax—absorbance of the λ maxima/um of either the standard or the unknown.

Based on the normalised absorbances, a threshold of ± 0.125 absorbance units for a potential match was set, and the percentage of absorbance points of the respective honey band that fell within the threshold was determined; the standard that yielded the highest percentage value was considered as a true potential match. To further refine the selection, Pearson’s correlation was also used to determine the relationship of the spectra of the unknown and the standards. If there were multiple candidates, the one with the highest correlation was deemed a true potential match, but only if the difference of the correlation of the matches was found to be greater than ± 0.100. Furthermore, the difference in thresholds when comparing the percent of points of the unknown that fell within that of a potential match was set at ± 10%.

Three spectral overlays were used in the matching process, namely, UV-Vis spectra after development, and also after derivatisation with NP-PEG and with VS reagents. Absorbances within the following wavelength ranges were considered important: 250 to 500 nm for UV-Vis prior to derivatisation and also after spraying with NP-PEG reagent, and 230 to 600 nm for UV-Vis after spraying with VSA reagent. The spectral overlays of fluorescence prior to and after derivatisation with NP-PEG and VSA reagent were only conducted if the UV-Vis spectra were not able to clearly identify the identity of the compound and region; 210 to 270 nm was considered as an important region for all fluorescence spectra.

Spectral matching was performed in the following sequence: UV-Vis prior to derivatisation, UV-Vis after derivatisation with NP-PEG reagent, UV-Vis after derivatisation with VS reagent and then fluorescence prior to and after any derivatisation (optional). A visual inspection of the spectral overlays was also conducted to confirm the match.

### 4.10. Database Testing and Validation

In order to determine whether the established database and developed filtering algorithm, including spectral overlays, were capable of correctly identifying an unknown compound, positive and negative control samples were analysed, where the positive controls were two artificial honeys spiked with two standards, respectively, which were included in the database to see if they could be correctly identified, and the negative control sample was an artificial honey spiked with a compound not included in the database, to confirm that the screening process would not yield any false-positive identification.

### 4.11. Inter-Method Validation

To further validate the detection of compounds using the HPTLC-derived databases, blinded test samples were also analysed in a separate laboratory using HPLC-DAD (unpublished protocol).

## 5. Conclusions

This study reported on a validated HPTLC-derived database system for phenolic compound determination in honey. Extensive research on the reported phenolic compounds in honey was performed prior to the database development to ensure that a comprehensive number of relevant standards could be included in the database. Two pairs of databases were developed that captured Rf values, colour (H°) at 254 nm and 366 nm, at 366 nm after derivatising with NP-PEG reagent, and at 366 nm, and white light in transmittance mode after derivatising with VSA reagent as well as fluorescence λ max and λ min and UV-Vis λ max after development, and fluorescence and UV-Vis λ max after derivatisation. These were all used as filtering variables to determine compound matches for an unknown sample against the database standards. An automated spectral overlay system was also developed to confirm that the database match correctly portrayed the identity of the unknown compounds. Validation with positive and negative controls in the form of artificial honey spiked with test compounds that were either present or absent from the database as well as the inter-method and inter-lab validations against HPLC-DAD analysis confirmed the reliability of the matching process.

The usefulness of the database was demonstrated by identifying a number of Manuka honey constituents. Leptosperine, mandelic acid, kojic acid, lepteridine, lumichrome, epigallocatechin gallate (EGCG), methyl syringate, gallic acid, mandelic acid, 2,3,4-trihydroxybenzoic acid, o-anisic acid and salicylic acid were identified using the matching strategy against the developed databases.

While the database system was demonstrated in this study to be a powerful tool in identifying unknown compounds in honey, the concept of using rich HPTLC data, not only including Rf values but also colour hues and UV and fluorescence spectra prior to and after derivatisation, can be assumed to also be useful in the identification of other natural product constituents, even in complex matrices, as long as meaningful reference standards can be identified for the construction of the database.

## Figures and Tables

**Table 1:** Datasets used for developing the database.

Name, Class and Code	Rf1	RF2	254 nm DEV H(°) and CE	366 nm DEV H(°) and CE	366 nm NP DER H(°) and CE	366 nm VSA DER H(°) and CE	T VSA DER H(°) and CE	F λ max and min	UV λ max	F NP λ	UVNP λ max	Fl VS λ	UV VS λ
5-Methoxyflavone (5-MF), Flavone, (1)	0.527	0.422	164	221	234	187	35	225, 254	268, 296, 331	245	297, 330	252	365
6-Hydroxyflavone-β-D-Glucoside (6-HF-β-D-Gluc.), Flavone, (2)	0.108	0.035	155	237	242	203	31	224, 257	265	244	311	250	371
Acacetin (Aca), Flavone, (3)	0.680	0.564	135	180	78	204	54	224, 257	270, 329	244	360	249	365
Apigenin (Api), Flavone, (4)	0.647	0.507	134	199	109	206	46	224, 256	271, 336	243	361	249	365
Baicalin (Bai), Flavone, (5)	0.056	0.028	135	185	181	198	44	224, 246	284	243	304, 350	259	374
Chrysin (Chr), Flavone, (6)	0.705	0.602	132	186	62	190	47	224, 255	270, 317	249	296, 341	249	383
Genkwanin (Genk), Flavone, (7)	0.657	0.533	139	46	126	229	47	224, 259	268, 338	243	367	249	399
Luteolin (Lut), Flavone, (8)	0.605	0.418	129	33	54	192	49	224, 253	268, 352	244	292, 460	251	376
Vitexin (Vit), Flavone, (9)	0.181	0.051	137	180	111	197	54	224, 262	273, 337	245	318, 365	249	373
Fisetin (Fis), Flavonol, (10)	0.568	0.368	138	176	31	184	52	224, 254	264, 325, 393	242	305, 338, 494	249	379
Galangin (Gal), Flavonol, (11)	0.737	0.642	131	172	172	194	42	224, 251	268, 311, 363	243	291, 341, 430	253	373
Isorhamnetin (Isor), Flavonol, (12)	0.665	0.536	136	180	167	191	43	224, 255	261, 364	250	293, 328, 434	252	388
Kaempferide (Kaep), Flavonol, (13)	0.713	0.616	129	144	161	188	45	223, 254	269, 330, 367	243	290, 365, 434	253	379
Kaempferol (Kaem), Flavonol, (14)	0.689	0.547	131	150	150	189	8	223, 254	269, 371	245	291, 369, 432	250	380
Myricetin (Myr), Flavonol, (15)	0.599	0.384	130	132	31	189	40	224, 253	261, 373	244	291, 488	252	388
Quercetin (Que), Flavonol, (16)	0.636	0.207	126	127	37	192	41	224, 254	261, 370	239	291, 483	253	384
Rutin (Rut), Flavonol, (17)	0.041	0.022	133	180	42	189	43	224, 255	264, 361	238	291, 470	254	384
Hesperetin (Hespt), Flavanone, (18)	0.681	0.559	137	169	160	202	20	222, 238	290	249	339	250	375
Hesperidin (Hespd), Flavanone, (19)	0.070	0.019	140	186	24	214	33	223, 235	286, 329	247	328, 418	248	378
Naringenin (Nar), Flavanone, (20)	0.703	0.591	137	177	160	211	24	223, 246	292	249	333	250	365
Naringin (Narg), Flavanone, (21)	0.070	0.025	134	130	149	206	21	222, 236	286, 333	247	328, 422	251	372
Pinocembrin (Pinoc), Flavanone, (22)	0.742	0.672	137	179	160	207	24	224, 241	293	246	338	251	370
Sakuranetin (Sak), Flavanone, (23)	0.721	0.623	137	160	102	200	16	223, 238	292	238	330, 409	253	368
Pinobanksin (Pinob), Flavanonol, (24)	0.715	0.598	137	191	174	199	10	224, 239	295	245	340	253	365
Taxifolin (Tax), Flavanonol, (25)	0.594	0.359	135	183	51	206	24	224, 240	292	244	302, 338	251	363
Catechin (Cat), Flavan-3-ol, (26)	0.533	0.256	138	142	85	211	18	224, 237	281	246	303	259	479
Epicatechin (Epi), Flavan-3-ol, (27)	0.517	0.228	137	138	135	209	11	224, 238	281	245	303	253	468
Epigallocatechin (EGC), Flavan-3-ol, (28)	0.460	0.130	137	177	111	201	2	225, 238	273	249	293	251	454
Epigallocatechin Gallate (EGCG), Flavan-3-ol, (29)	0.440	0.098	136	188	242	199	12	225, 240	282	246	332	249	495
Biochanin A (Bio), Isoflavone, (30)	0.711	0.603	133	97	156	195	28	224, 252	262	238	288, 393	248	367
Daidzein (Dai), Isoflavone, (31)	0.616	0.422	137	161	226	226	29	222, 251	251, 305	242	309	248	395
Formononetin (For), Isoflavone, (32)	0.663	0.517	147	195	194	179	38	223, 250	251, 306	246	310	249	397
Genistein (Gen), Isoflavone, (33)	0.684	0.549	128	181	136	211	34	224, 253	260	249	289, 395	248	419
Genistin (Genist), Isoflavone, (34)	0.211	0.072	133	177	148	200	26	222, 255	261	249	291, 399	247	399
t-Chalcone (t-Chal), Chalcone, (35)	0.754	0.699	137	113	79	231	40	221, 257	310	232	308	249	361
2,3,4-Trihydroxybenzoic Acid (2,3,4-THBA), HBAD, (36)	0.623	0.437	138	185	216	226	21	223, 256	267	236	306	248	431
2,3,4-Trimethoxybenzoic Acid (2,3,4-TMBA), HBAD, (37)	0.611	0.479	142	120	147	241	22	225, 254	261	236	261	248	447
2,4,5-Trimethoxybenzoic Acid (2,4,5-TMBA), HBAD, (38)	0.496	0.347	152	256	255	253	340	223, 254	261, 312	241	311	252	370
3,5-Dihydroxybenzoic Acid (3,5-DHBA), HBAD, (39)	0.615	0.428	138	191	217	222	33	225, 251	251, 308	239	308	248	410
Benzoic Acid (BA), HBAD, (40)	0.696	0.570	136	185	159	199	48	211, 241	276, 316	246	277	251	371
Cuminic Acid (CuA), HBAD, (41)	0.710	0.525	136	180	185	205	2	221, 246	243	223	252	253	247
Ellagic Acid (EllA), HBAD, (42)	0.017	0.020	130	212	207	221	40	223, 253	277, 389	248	291	318	383
Eudesmic Acid (EudA), HBAD, (43)	0.629	0.504	139	180	203	219	41	228, 254	264	232	291	248	252
Gallic Acid (GA), HBAD, (44)	0.544	0.321	135	152	256	217	43	225, 257	272	245	322	249	389
Gentisic Acid (GenA), HBAD, (45)	0.634	0.508	143	224	246	213	49	224, 246	326	241	352	247	355
Leptosperine (Leps), HBAD, (46)	0.017	0.012	137	188	207	223	45	229, 257	265	230	292	248	399
Methyl Paraben (Mpar), HBAD, (47)	0.683	0.573	128	179	220	228	111	224, 252	257	238	263	247	251
Methyl Syringate (MS), HBAD, (48)	0.634	0.524	138	175	203	219	43	237, 258	277	241	291	248	351
Methyl-3,4,5-trimethoxybenzoate (M-3,4,5-TMBz), HBAD, (49)	0.688	0.589	141	186	120	202	48	230, 256	265	239	294	245	432
m-Hydroxybenzoic Acid (m-HBA), HBAD, (50)	0.649	0.499	138	181	184	232	38	223, 245	298	253	298	251	422
m-Toluic Acid (m-TA), HBAD, (51)	0.697	0.616	137	160	189	206	25	221, 242	284	243	287	249	349
o-Anisic Acid (o-AA), HBAD, (52)	0.610	0.499	138	206	193	225	43	221, 243	299	243	295	248	452
o-Toluic Acid (o-TA), HBAD, (53)	0.662	0.633	138	175	215	222	33	221, 240	281	255	293	248	437
p-Hydroxybenzoic Acid (p-HBA), HBAD, (54)	0.656	0.513	130	184	180	212	28	225, 252	257	232	261	245	445
Protocatechuic Acid (PrA), HBAD, (55)	0.595	0.398	136	183	246	214	46	225, 255	261, 295	242	326	249	474
Resorcylic Acid (ReA), HBAD, (56)	0.646	0.504	144	214	226	202	57	224, 249	251, 313	241	331	254	348
Salicylic Acid (SA), HBAD, (57)	0.685	0.582	140	211	181	205	48	223, 242	301	254	326	247	369
Syringic Acid (SyA), HBAD, (58)	0.601	0.444	137	192	215	218	28	225, 258	277	240	290	247	351
Vanillic Acid (VA), HBAD, (59)	0.643	0.504	136	192	209	218	32	228, 255	263, 294	240	295	251	492
Vanillic Acid Methyl Ester (VAME), HBAD, (60)	0.671	0.570	135	179	231	207	71	225, 254	263, 294	243	297	248	423
Caffeic Acid (CaA), HCAD, (61)	0.597	0.421	139	211	197	215	38	224, 250	321	254	374	248	358
Caffeic Acid Dimethyl Ether (CADE), HCAD, (62)	0.625	0.501	142	239	223	212	101	223, 247	317	238	308	248	350
Caffeic Acid Phenetyl Ester (CAPE), HCAD, (63)	0.684	0.554	140	235	209	240	27	225, 250	325	254	392	249	411
Chlorogenic Acid (ChlA), HCAD, (64)	0.112	0.033	138	196	201	224	43	222, 250	329	249	379	249	356
Ferulic Acid (FA), HCAD, (65)	0.636	0.508	139	209	206	215	41	224, 246	319	239	317	248	353
Isoferulic Acid (IFA), HCAD, (66)	0.609	0.459	140	227	216	215	136	224, 248	320	239	317	249	350
m-Coumaric Acid (m-CA), HCAD, (67)	0.644	0.493	143	215	220	208	49	224, 252	280	228	288	252	344
Methyl Ferulate (MF), HCAD, (68)	0.682	0.576	138	236	228	214	83	224, 250	324	233	327	251	354
Neochlorogenic Acid (NChlA), HCAD, (69)	0.057	0.014	141	228	217	205	48	224, 256	329	250	382	248	365
o-Coumaric Acid (o-CA), HCAD, (70)	0.655	0.519	146	205	204	223	41	224, 258	279, 321	253	318	248	427
p-Coumaric Acid (p-CA), HCAD, (71)	0.644	0.510	136	199	205	207	41	223, 258	307	255	298	248	348
p-m-Cinnamic Acid (p-MCA), HCAD, (72)	0.670	0.556	137	191	131	245	10	222, 257	307	244	296	249	425
Rosmarinic Acid (RosA), HCAD, (73)	0.692	0.259	131	175	134	226	43	224, 248	307	242	307, 389	249	437
Sinapic Acid (SinA), HCAD, (74)	0.533	0.439	139	224	198	224	46	222, 245	328	243	320	248	359
t-Cinnamic Acid (t-CA), HCAD, (75)	0.586	0.596	140	242	222	202	44	229, 261	279	242	289	248	391
Trans-p-Coumaric Acid Methyl Ester (t-p-CAME), HCAD, (76)	0.697	0.584	136	193	202	205	49	222, 237	309	245	314	250	437
3,4-Dihydroxyphenylacetic Acid (3,4-DHPAA), HPAAD, (77)	0.592	0.365	137	180	235	270	11	224, 238	283	242	305	250	422
Homogentisic Acid (HGA), HPAAD, (78)	0.569	0.348	139	184	132	237	49	224, 235	294	253	298	250	396
Homovanillic Acid (HVA), HPAAD, (79)	0.609	0.441	137	177	213	230	67	222, 240	282	244	288	248	452
Mandelic Acid (ManA), HPAAD, (80)	0.530	0.347	139	165	208	235	0	223, 241	292	250	329	249	496
Phenylacetic Acid (PAA), HPAAD, (81)	0.678	0.589	136	175	343	242	23	239, 258	260	249	300	248	413
p-Hydroxyphenylacetic Acid (p-HPAA), HPAAD, (82)	0.643	0.471	138	179	180	265	11	222, 239	278	249	285	250	426
L-β -Phenyllactic Acid (L-β -PLA), HPLAD, (83)	0.701	0.640	138	186	226	220	26	233, 259	261	250	321	248	498
DL-p-Phenyllactic Acid (DL-p-HPLA), HPLAD, (84)	0.636	0.466	139	120	171	244	25	223, 238	278	243	285	256	378
p-Methoxy Phenyllactic Acid (p-MPLA), HPLAD, (85)	0.665	0.536	138	179	192	228	38	222, 236	276	250	284	250	400
3-Phenylpropanoic Acid (3-PPA), HPPAD, (86)	0.685	0.586	139	178	230	231	166	230, 258	260	255	264	248	437
Phloretic Acid (PhlA), HPPAD, (87)	0.650	0.483	141	188	190	229	33	221, 232	278	254	288	249	422
2-Methoxy-4-vinylphenol (2-M4-VPh), AMPh, (88)	0.707	0.631	122	189	191	308	339	224, 251	266	224	291	264	537
p-Methoxyphenol (p-MPh), AMPh, (89)	0.679	0.583	134	171	235	227	37	223, 231	288	241	296	249	437
4-Methylpyrocatechol (4-MPCat), Aph, (90)	0.675	0.546	137	180	161	242	345	223, 256	283	245	306	249	426
Isopseudocumenol (Isops), Aph, (91)	0.741	0.676	138	179	190	273	348	247, 268	279	241	283	251	382
Thymol (Thy), Aph, (92)	0.756	0.693	140	120	131	287	355	244, 268	278	244	284	251	465
Acetaminophen (Acet), p-AmPh, (93)	0.486	0.295	121	171	183	226	37	221, 250	251	225	259, 291	250	396
Pyrogallol (Pyrog), Phenol, (94)	0.624	0.445	137	186	51	224	21	224, 255	270	243	291	263	520
Pyrocatechol (Pyroc), Phenol, (95)	0.685	0.554	137	181	75	219	36	226, 265	277	245	301	250	520
2-methylbenzaldehyde (2-MBzd), HBzd, (96)	0.722	0.676	134	188	194	287	17	222, 253	255	253	288	249	421
p-Anisaldehyde (p-Anzd), HBzd, (97)	0.687	0.605	120	203	347	328	38	224, 255	286	239	291	246	437
Protocatechualdehyde (PrCatd), HBzd, (98)	0.619	0.453	135	232	233	240	29	221, 240	283, 314	240	303	248	422
Vanillin (Van), HBzd, (99)	0.659	0.548	136	222	224	212	42	221, 238	284, 312	248	314	250	252
2′-Hydroxyacetophenone ( 2′-HAPhn), HAPhn, (100)	0.717	0.672	133	152	180	206	31	228, 254	256, 330	238	311	248	373
2′-Methoxyacetophenone ( 2′-MAPhn), HAPhn, (101)	0.692	0.628	139	241	204	188	0	225, 251	252, 312	231	311	267	367
Dibenzyl Oxalate (DO), OE, (102)	0.639	0.531	139	199	278	245	62	222, 257	262, 393	221	290	245	385
Absiscic Acid (AbsA), Non-phenolic, (103)	0.612	0.435	127	171	205	340	356	221, 251	268	223	265	250	385
Benzophenone (Benzph), Non-phenolic, (104)	0.737	0.652	120	171	196	240	40	224, 253	260	235	261, 288	239	251
Kojic Acid (KojA), Non-Phenolic, (105)	0.308	0.171	136	217	228	231	45	221, 253	277	245	306	249	420
Lepteridine (Leptd), Non-Phenolic, (106)	0.322	0.217	148	195	209	242	21	227, 249	329	247	332	248	365
Lumichrome (Lum), Non-Phenolic, (107)	0.482	0.326	153	209	221	216	52	225, 257	261, 357	249	361	249	391

Legend: **Rf1**—retention factor in MPA, **Rf2**—retention factor in MPB, **254 nm DEV H° and C**—hue and colour equivalent at 254 nm prior to derivatisation, **366 nm DEV H° and C**—hue and colour equivalent at 366 nm prior to derivatisation, **366 nm NP H° and C**—hue and colour equivalent at 366 nm after derivatisation w/ NP-PEG-derivatisation reagent, **366 nm VS H° and C**—hue and colour equivalent at 366 nm after derivatisation w/ VSA-derivatisation reagent, **T VSA H° and C**—hue and colour equivalent at transmittance in white light after derivatisation w/ VSA-derivatisation reagent, **Fl DEV λ max and λ min**—fluorescence λ max and λ min prior to derivatisation, **UV DEV λ max**—UV-Vis λ max prior to derivatisation, **Fl NP λ max**—fluorescence λ max after derivatisation with NP-PEG reagent, **UV NP λ max**—UV-Vis λ max after derivatisation with NP-PEG reagent, **Fl VS λ max**—fluorescence λ max after derivatisation with VSA reagent, **UV-Vis λ max**—UV-Vis λ max after derivatisation with VSA reagent. Note: coloured cells represent colours as seen on HPTLC plate. Subclass (see Tables S1–S14,): **HCAD**—hydroxycinnamic acid and derivatives, **HBAD**—hydroxybenzoic acid and derivatives, **HPAAD**—hydroxyphenyl acetic acid and derivatives, **HPLAD**—hydroxyphenyllactic acid and derivatives, **HPPAD**—hydroxyphenylpropionic acid and derivatives, **AMPh**—alkylmethoxyphenol, **APh**—alkylphenol, **p-AmPh**—p-aminophenol, **HBzd**—hydroxybenzaldehyde and derivatives, **HAPh**—hydroxyacetophenone and derivatives, **OE**—oxalate ester, NP—non-phenolics

**Table 2:** Result of compound matching for test compound A using DB 1A.

Name and Code	Rf 1	H° DEV 254 nm	H° DEV 366 nm	H° NP 366 nm	Fl DEV λ	Fl DEV λ m	UV DEV λ_1_	UV DEV λ_2_	UV DEV λ_3_	Fl NP λ	UV NP λ_1_	UV NP λ_2_	UV NP λ_3_
Test Compound A	0.608	139	180	209	225	258	276	0	0	239	288	0	0
**Number of Potential Matches**	**42**	**42**	**40**	**31**	**31**	**25**	**11**	**6**	**6**	**5**	**5**	**5**	**5**
2,3,4-THBA, (36)	0.589	138	185	216	223	256	267	0	0	236	306	0	0
Eudesmic acid, (43)	0.617	139	180	203	228	254	264	0	0	232	291	0	0
Methyl Syringate, (48)	0.610	138	175	203	237	258	277	0	0	241	291	0	0
Syringic Acid, (58)	0.577	137	192	215	225	258	277	0	0	240	290	0	0
m-Coumaric Acid, (67)	0.633	143	215	220	224	252	280	0	0	228	288	0	0
Abscisic Acid, (103)	0.598	127	171	205	221	251	268	0	0				
Isorhamnetin, (12)	0.651	136	180	167	224	255	261						
Kaempferol, (14)	0.654	131	150	150	223	254	269						
Protocatechuic Acid, (55)	0.577	136	183	246	225	255	261						
Vanillic Acid, (59)	0.623	136	192	209	228	255	263						
o-Coumaric Acid, (70)	0.646	146	205	204	224	258	279						
3,5-DHBA, (39)	0.594	138	191	217	225	251							
Gentisic Acid, (45)	0.626	143	224	246	224	246							
m-HBA, (50)	0.644	138	181	184	223	245							
o-Anisic Acid, (52)	0.589	138	206	193	221	243							
p-HBA, (54)	0.637	130	184	180	225	252							
Resorcylic Acid, (56)	0.630	144	214	226	224	249							
Caffeic Acid, (61)	0.589	139	211	197	224	250							
CADE, (62)	0.609	142	239	223	223	247							
Ferulic Acid, (65)	0.616	139	209	206	224	246							
Isoferulic Acid, (66)	0.600	140	227	216	224	248							
p-Coumaric Acid, (71)	0.630	136	199	205	223	258							
Daidzein, (31)	0.600	137	161	203	222	251							
Formononetin, (32)	0.637	147	195	194	223	250							
Sinapic Acid, (74)	0.630	139	224	198	222	245							
HVA, (79)	0.654	137	177	213	222								
p-HPAA, (82)	0.620	138	179	180	222								
DL-p-PLA, (84)	0.616	139	120	171	223								
Phloretic Acid, (87)	0.630	141	188	190	221								
ProCatd, (98)	0.594	135	232	233	221								
Vanillin, (99)	0.637	136	222	224	221								
Acacetin, (3)	0.649	135	180										
Apigenin, (4)	0.617	134	199										
Myricetin, (15)	0.587	130	132										
Quercetin, (16)	0.617	126	127										
Taxifolin, (25)	0.591	135	183										
2,3,4-TMBA, (37)	0.602	142	120										
p-MCA, (72)	0.650	137	191										
Rosmarinic Acid, (73)	0.630	131	175										
Pyrogallol, (94)	0.602	137	186										
Genkwanin, (7)	0.653	139											
Luteolin, (8)	0.584	129											

Legend: **Rf1**—retention factor in MPA, **H° DEV 254 nm**—hue equivalent at 254 nm prior to derivatisation, **H° DEV 366 nm**—hue equivalent at 366 nm prior to derivatisation, **H° NP 366 nm**—hue equivalent at 366 nm after derivatisation w/ NP-PEG-derivatisation reagent, **Fl DEV λ**—fluorescence λ max prior to derivatisation, **Fl DEV λ m**—fluorescence λ min prior to derivatisation, **UV DEV λ_1-3_**—UV-Vis λ max prior to derivatisation, **Fl NP λ**—fluorescence λ max after derivatisation with NP-PEG reagent, **UV NP λ_1-3_**—UV-Vis λ max after derivatisation with NP-PEG reagent. Note: coloured cells represent colours as seen on HPTLC plate.

**Table 3:** Result of compound matching for test compound A using DB 1B.

Name and Code	Rf 1	H° DEV 254 nm	H° DEV 366 nm	H° VSA 366 nm	H° T VSA	Fl DEV λ	Fl DEV λ m	UV DEV λ_1_	UV DEV λ_2_	UV DEV λ_3_	Fl VS λ	UV VS λ
Test Compound A	0.580	137	180	204	14	224	257	277	0	0	251	354
**Number of Potential Matches**	**32**	**32**	**32**	**29**	**27**	**27**	**22**	**8**	**5**	**5**	**5**	**2**
Methyl Syringate, (48)	0.610	138	175	219	43	237	258	277	0	0	248	351
Syringic Acid, (58)	0.577	137	192	218	28	225	258	277	0	0	247	351
2,3,4-THBA, (36)	0.589	138	185	226	21	223	256	267	0	0	248	
Eudesmic Acid, (43)	0.617	139	180	219	41	228	254	264	0	0	248	
Apigenin, (4)	0.617	134	199	206	46	224	256	271				
Fisetin, (10)	0.540	138	176	184	52	224	254	264				
Vanillic Acid, (59)	0.623	136	192	218	32	228	255	263				
Myricetin, (15)	0.587	130	132	189	40	224	253					
Quercetin, (16)	0.617	126	127	192	41	224	254					
Daidzein, (31)	0.600	137	161	226	29	222	251					
2,3,4-TMBA, (37)	0.602	142	120	241	22	225	254					
3,5-DHBA, (39)	0.594	138	191	222	33	225	251					
Gentisic Acid, (45)	0.626	143	224	213	49	224	246					
o-Anisic Acid, (52)	0.589	138	206	225	43	221	243					
Protocatechuic Acid, (55)	0.577	136	183	214	46	225	255					
Resorcylic Acid, (56)	0.630	144	214	202	57	224	249					
Caffeic Acid, (61)	0.589	139	211	215	38	224	250					
Ferulic Acid, (65)	0.616	139	209	215	41	224	246					
p-Coumaric Acid, (71)	0.630	136	199	207	41	223	258					
Rosmarinic Acid, (73)	0.630	131	175	226	43	224	248					
Sinapic Acid, (74)	0.630	139	224	224	46	222	245					
Taxifolin, (25)	0.591	135	183	206	24	224						
HGA, (78)	0.545	139	184	237	49	224						
DL-p-HPLA, (84)	0.616	139	120	244	25	223						
Phloretic Acid, (87)	0.630	141	188	229	33	221						
Procatd, (98)	0.594	135	232	240	29	221						
CADE, (62)	0.609	142	239	212								
Isoferulic Acid, (66)	0.600	140	227	215								
3,4-DHPAA, (77)	0.556	137	180									
p-HPAA, (82)	0.620	138	179									
Abscisic Acid, (103)	0.598	127	171									
Luteolin, (8)	0.584	129										

Legend: Rf1—retention factor in MPA, H° DEV 254 nm—hue and colour equivalent at 254 nm prior to derivatisation, H° DEV 366 nm—hue and colour equivalent at 366 nm prior to derivatisation, H° VS 366 nm—hue and colour equivalent at 366 nm after derivatisation w/ VSA-derivatisation reagent, H° T VS—hue and colour equivalent at transmittance in white light after derivatisation w/ VSA-derivatisation reagent; Fl DEV λ max—fluorescence λ max prior to derivatisation, Fl DEV λ m—fluorescence λ min prior to derivatisation, UV DEV λ_1-3_—UV-Vis λ max prior to derivatisation, Fl VS λ— fluorescence λ max after derivatisation with VSA reagent, UV-Vis λ—UV-Vis λ max after derivatisation with VSA reagent. Note: coloured cells represent colours as seen on HPTLC plate.

**Table 4:** Results of compound matching for test compound A using DB 2A.

Name and Code	Rf 2	H° DEV 254 nm	H° DEV 366 nm	H° NP 366 nm	Fl DEV λ	Fl DEV λ m	UV DEV λ_1_	UV DEV λ_2_	UV DEV λ_3_	Fl NP λ	UV NP λ_1_	UV NP λ_2_
Test Compound A	0.435	139	180	204	225	258	276	0	0	239	288	0
**Number of Potential Matches**	**18**	**18**	**17**	**15**	**15**	**10**	**5**	**3**	**3**	**2**	**2**	**2**
2,3,4-THBA, (36)	0.437	138	185	216	223	256	267	0	0	236	306	0
Syringic Acid, (58)	0.444	137	192	215	225	258	277	0	0	240	290	0
Absiscic Acid, (103)	0.435	127	171	205	221	251	268	0	0			
5-Methoxyflavone, (1)	0.422	164	221	234	225	254	268					
Protocatechuic Acid, (55)	0.398	136	183	246	225	255	261					
3,5-DHBA, HBAD, (39)	0.428	138	191	217	225	251						
Caffeic Acid, (61)	0.421	139	211	197	224	250						
Isoferulic Acid, (66)	0.459	140	227	216	224	248						
Sinapic Acid, (74)	0.439	139	224	198	222	245						
Daidzein, (31)	0.422	137	161	226	222	251						
HVA, (79)	0.441	137	177	213	222							
p-HPAA, (82)	0.471	138	179	180	222							
DL-p-HPLA, (84)	0.466	139	120	171	223							
Phloretic Acid, (87)	0.483	141	188	190	221							
Procatd, (98)	0.453	135	232	233	221							
2,3,4-TMBA, (37)	0.479	142	120									
Pyrogallol, (94)	0.445	137	186									
Luteolin, (8)	0.418	129										

Legend: Rf2—retention factor in MPB, H° DEV 254 nm—hue equivalent at 254 nm prior to derivatisation, H° DEV 366 nm—hue equivalent at 366 nm prior to derivatisation, H° NP 366 nm—hue equivalent at 366 nm after derivatisation w/ NP-PEG-derivatisation reagent, Fl DEV λ—fluorescence λ max prior to derivatisation, Fl DEV λ m—fluorescence λ min prior to derivatisation, UV DEV λ_1-3_—UV-Vis λ max prior to derivatisation, Fl NP λ—fluorescence λ max after derivatisation with NP-PEG reagent, UV NP λ_1-3_—UV-Vis λ max after derivatisation with NP-PEG reagent. Note: coloured cells represent colours as seen on HPTLC plate.

**Table 5:** Results of compound matching for test compound A using DB 2B.

Name and Code	Rf 2	H° DEV 254 nm	H° DEV 366 nm	H° VSA 366 nm	H° T VSA	Fl DEV λ	Fl DEV λ m	UV DEV λ_1_	UV DEV λ_2_	UV DEV λ_3_	Fl VS λ	UV VS λ
Test Compound A	0.435	137	180	204	14	224	257	277	0	0	251	354
**Number of Potential Matches**	**18**	**18**	**17**	**15**	**14**	**14**	**10**	**4**	**3**	**3**	**3**	**1**
Syringic Acid, (58)	0.444	137	192	218	28	225	258	277	0	0	247	351
2,3,4-THBA, (36)	0.437	138	185	226	21	223	256	267	0	0	248	
Pyrogallol, (94)	0.445	137	186	224	21	224	255	270	0	0	263	
5-Methoxyflavone, (1)	0.422	164	221	187	35	225	254	268				
Daidzein, (31)	0.422	137	161	226	29	222	251					
2,3,4-TMBA, (37)	0.479	142	120	241	22	225	254					
3,5-DHBA, (39)	0.428	138	191	222	33	225	251					
Protocatechuic Acid, (55)	0.398	136	183	214	46	225	255					
Caffeic Acid, (61)	0.421	139	211	215	38	224	250					
Sinapic Acid, (74)	0.439	139	224	224	46	222	245					
Homovanillic Acid, (79)	0.441	137	177	230	67	222						
DL-p-HPLA, (84)	0.466	139	120	244	25	223						
Phloretic Acid, (87)	0.483	141	188	229	33	221						
Protocatechualdehyde, (98)	0.453	135	232	240	29	221						
Isoferulic Acid, (66)	0.459	140	227	215								
p-HPAA, (82)	0.471	138	179									
Absiscic Acid, (103)	0.435	127	171									
Luteolin, (8)	0.418	129										

Legend: Rf2—retention factor in MPB, H° DEV 254 nm—hue and colour equivalent at 254 nm prior to derivatisation, H° DEV 366 nm—hue and colour equivalent at 366 nm prior to derivatisation, H° VSA 366 nm—hue and colour equivalent at 366 nm after derivatisation w/ VSA-derivatisation reagent, H° T VSA—hue and colour equivalent at transmittance in white light after derivatisation w/ VSA derivatisation reagent, Fl DEV λ max—fluorescence λ max prior to derivatisation, Fl DEV λ m—fluorescence λ min prior to derivatisation, UV DEV λ_1-3_—UV-Vis λ max prior to derivatisation, Fl VS λ—fluorescence λ max after derivatisation with VSA reagent, UV-Vis λ—UV-Vis λ max after derivatisation with VSA reagent. Note: coloured cells represent colours as seen on HPTLC plate.

**Table 6:** Results of compound matching for test compound B using DB 1A.

Name and Code	Rf 1	H° DEV 254 nm	H° DEV 366 nm	H° NP 366 nm	Fl DEV λ	Fl DEV λ m	UV DEV λ_1_	UV DEV λ_2_	UV DEV λ_3_	Fl NP λ	UV NP λ_1_	UV NP λ_2_	UV NP λ_3_
Test Compound B	0.685	121	152	147	224	255	269	369	0	245	288	373	433
**Number of Potential Matches**	**48**	**48**	**42**	**30**	**28**	**23**	**16**	**2**	**2**	**2**	**2**	**2**	**1**
Kaempferol, (14)	0.654	131	150	150	223	254	269	371	0	245	291	369	432
Isorhamnetin, (12)	0.651	136	180	167	224	255	261	364	0	250	293	434	
Galangin, (11)	0.702	131	172	172	224	251	268						
Kaempferide, (13)	0.690	129	144	161	223	254	269						
Biochanin A, (30)	0.692	133	97	156	224	252	262						
Genistein, (33)	0.663	128	181	136	224	253	260						
Benzoic Acid, (40)	0.663	136	185	159	211	241	276						
Methyl-3,4,5-TMBz, (49)	0.670	141	186	120	230	256	265						
m-Toluic Acid, (51)	0.681	137	160	189	221	242	284						
p-HBA, (54)	0.637	130	184	180	225	252	257						
o-Coumaric Acid, (70)	0.646	146	205	204	224	258	279						
2-M-4-VPh, (88)	0.688	122	189	191	224	251	266						
4-MPCat, (90)	0.674	137	180	161	223	256	283						
2-MBzd, (96)	0.709	134	188	194	222	253	255						
2′-HAPh, (100)	0.704	133	152	180	228	254	256						
Benzophenone, (104)	0.727	120	171	196	224	253	260						
Naringenin, (20)	0.680	137	177	160	223	246							
Pinocembrin, (22)	0.723	137	179	160	224	241							
Formononetin, (32)	0.637	147	195	194	223	250							
Cuminic Acid, (41)	0.697	136	180	185	221	246							
m-HBA, (50)	0.644	138	181	184	223	245							
Salicylic Acid, (57)	0.679	140	211	181	223	242							
p-MCA, (72)	0.650	137	191	131	222	257							
Hesperetin, (18)	0.667	137	169	160	222								
Sakuranetin, (23)	0.699	137	160	102	223								
Pinobanksin, (24)	0.697	137	191	174	224								
Trans-p-CAME, (76)	0.674	136	193	202	222								
p-MPLA, (85)	0.658	138	179	192	222								
Isops, (91)	0.725	138	179	190									
Thymol, (92)	0.732	140	120	131									
Acacetin, (3)	0.649	135	180										
Chrysin, (6)	0.672	132	186										
t-Chalcone, (35)	0.734	137	113										
Methyl Paraben, (47)	0.676	128	179										
o-Toluic Acid, (53)	0.692	138	175										
VAME, (60)	0.658	135	179										
HVA, (79)	0.654	137	177										
Phenylacetic Acid, (81)	0.670	136	175										
3-PPA, (86)	0.683	139	178										
p-Methoxyphenol, (89)	0.685	134	171										
Pyrocatechol, (95)	0.662	137	181										
p-Anisaldehyde, (97)	0.660	120	203										
Genkwanin, (7)	0.653	139											
CAPE, (63)	0.676	140											
Methyl Ferulate, (68)	0.667	138											
t-Cinnamic Acid, (75)	0.672	140.3											
Vanillin, (99)	0.637	136.2											
2′-MAPh, (101)	0.669	139.1											

Legend: Rf1—retention factor in MPA, H° DEV 254 nm—hue equivalent at 254 nm prior to derivatisation, H° DEV 366 nm—hue equivalent at 366 nm prior to derivatisation, H° NP 366 nm—hue equivalent at 366 nm after derivatisation w/ NP-PEG-derivatisation reagent, Fl DEV λ—fluorescence λ max prior to derivatisation, Fl DEV λ m—fluorescence λ min prior to derivatisation, UV DEV λ_1-3_—–UV-Vis λ max prior to derivatisation, Fl NP λ—fluorescence λ max after derivatisation with NP-PEG reagent, UV NP λ_1-3_—UV-Vis λ max after derivatisation with NP-PEG reagent. Note: coloured cells represent colours as seen on HPTLC plate.

**Table 7:** Results of compound matching for test compound B using DB 1B.

Name and Code	Rf 1	H° DEV 254 nm	H° DEV 366 nm	H° VSA 366 nm	H° T VSA	Fl DEV λ	Fl DEV λ m	UV DEV λ_1_	UV DEV λ_2_	UV DEV λ_3_	Fl VS λ	UV VS λ
Test Compound B	0.650	137	174	190	36	223	256	269	371	0	251	377
**Number of Potential Matches**	**58**	**58**	**51**	**48**	**46**	**45**	**34**	**22**	**3**	**3**	**3**	**3**
Isorhamnetin, (12)	0.651	136	180	191	43	224	255	261	364	0	252	388
Kaempferol, (14)	0.654	131	150	189	8	223	254	269	371	0	250	380
Quercetin, (16)	0.617	126	127	192	41	224	254	261	370	0	253	384
Acacetin, (3)	0.649	135	180	204	54	224	257	270				
Apigenin, (4)	0.617	134	199	206	46	224	256	271				
Chrysin, (6)	0.672	132	186	190	47	224	255	270				
Kaempferide, (13)	0.690	129	144	188	45	223	254	269				
Genistein, (33)	0.663	128	181	211	34	224	253	260				
2,3,4-TMBA, (37)	0.602	142	120	241	22	225	254	261				
Benzoic Acid, (40)	0.663	136	185	199	48	211	241	276				
Eudesmic Acid, (43)	0.617	139	180	219	41	228	254	264				
Methyl Syringate, (48)	0.610	138	175	219	43	237	258	277				
Methyl-3,4,5-TMBz, (49)	0.670	141	186	202	48	230	256	265				
m-Toluic Acid, (51)	0.681	137	160	206	25	221	242	284				
p-HBA, (54)	0.637	130	184	212	28	225	252	257				
Vanillic Acid, (59)	0.623	136	192	218	32	228	255	263				
VAME, (60)	0.658	135	179	207	71	225	254	263				
m-Coumaric Acid, (67)	0.633	143	215	208	49	224	252	280				
o-Coumaric Acid, (70)	0.646	146	205	223	41	224	258	279				
4-MPCat, (90)	0.674	137	180	242	345	223	256	283				
Pyrogallol, (94)	0.602	137	186	224	21	224	255	270				
Pyrocatechol, (95)	0.662	137	181	219	36	226	265	277				
Naringenin, (20)	0.68	137	177	211	24	223	246					
Formononetin, (32)	0.637	147	195	179	38	223	250					
Cuminic Acid, (41)	0.697	136	180	205	2	221	246					
Gentisic Acid, (45)	0.626	143	224	213	49	224	246					
m-HBA, (50)	0.644	138	181	232	38	223	245					
Resorcylic Acid, (56)	0.630	144	214	202	57	224	249					
Salicylic Acid, (57)	0.679	140	211	205	48	223	242					
Ferulic Acid, (65)	0.616	139	209	215	41	224	246					
p-Coumaric Acid, (71)	0.630	136	199	207	41	223	258					
p-MCA, (72)	0.650	137	191	245	10	222	257					
Rosmarinic Acid, (73)	0.630	131	175	226	43	224	248					
Sinapic Acid, (74)	0.630	139	224	224	46	222	245					
Hesperetin, (18)	0.667	137	169	202	20	222						
Sakuranetin, (23)	0.699	137	160	200	16	223						
Pinobanksin, (24)	0.697	137	191	199	10	224						
o-Toluic Acid, (53)	0.692	138	175	222	33	221						
Trans-p-CAME, (76)	0.674	136	193	205	49	222						
HVA, (79)	0.654	137	177	230	67	222						
DL-p-PLA, (84)	0.616	139	120	244	25	223						
p-MPLA, (85)	0.658	138	179	228	38	222						
Phloretic Acid, (87)	0.630	141	188	229	33	221						
p-Methoxyphenol, (89)	0.685	134	171	227	37	223						
Vanillin, (99)	0.637	136	222	212	42	221						
Phenylacetic Acid, (81)	0.670	136	175	242	23							
Methyl Paraben, (47)	0.676	128	179	228								
3-PPA, (86)	0.683	139	178	231								
p-HPAA, (82)	0.620	138	179									
2-M-4-VPh, (88)	0.688	122	189									
p-Anisaldehyde, (97)	0.66	120	203									
Genkwanin, (7)	0.653	139										
Biochanin A, (30)	0.692	133										
CADE, (62)	0.609	142										
CAPE, (63)	0.676	140										
Methyl Ferulate, (68)	0.667	138										
t-Cinnamic Acid, (75)	0.672	140										
2′-MAPh, (101)	0.669	139										

Legend: Rf2—retention factor in MPB, H° DEV 254 nm—hue and colour equivalent at 254 nm prior to derivatisation, H° DEV 366 nm—hue and colour equivalent at 366 nm prior to derivatisation, H° VS 366 nm—hue and colour equivalent at 366 nm after derivatisation w/ VSA-derivatisation reagent, H° T VS—hue and colour equivalent at transmittance in white light after derivatisation w/ VSA-derivatisation reagent, Fl DEV λ max—fluorescence λ max prior to derivatisation, Fl DEV λ m—fluorescence λ min prior to derivatisation, UV DEV λ_1-3_—UV-Vis λ max prior to derivatisation, Fl VS λ—fluorescence λ max after derivatisation with VSA reagent, UV-Vis λ—UV-Vis λ max after derivatisation with VSA reagent. Note: coloured cells represent colours as seen on HPTLC plate.

**Table 8:** Results of compound matching for test compound B using DB 2A.

Name and Code	Rf 2	H° DEV 254 nm	H° DEV 366 nm	H° NP 366 nm	Fl DEV λ	Fl DEV λ m	UV DEV λ_1_	UV DEV λ_2_	UV DEV λ_3_	Fl NP λ	UV NP λ_1_	UV NP λ_2_	UV NP λ_3_
Test Compound B	0.520	135	152	147	224	255	269	369	0	245	433	288	373
**Number of Potential Matches**	**33**	**33**	**26**	**22**	**22**	**18**	**11**	**2**	**2**	**2**	**2**	**2**	**1**
Kaempferol, (14)	0.547	131	150	150	223	254	269	371	0	245	432	291	369
Isorhamnetin, (12)	0.536	136	180	167	224	255	261	364	0	250	434	293	
Apigenin, (4)	0.507	134	199	109	224	256	271						
Genistein, (33)	0.549	128	181	136	224	253	260						
2,3,4-TMBA, (37)	0.479	142	120	147	225	254	261						
Benzoic Acid, (40)	0.570	136	185	159	211	241	276						
Eudesmic Acid, (43)	0.504	139	180	203	228	254	264						
Methyl Syringate, (48)	0.524	138	175	203	237	258	277						
p-Hydroxybenzoic Acid, (54)	0.513	130	184	180	225	252	257						
o-Coumaric Acid, (70)	0.519	146	205	204	224	258	279						
4-Methylpyrocatechol, (90)	0.546	137	180	161	223	256	283						
Formononetin, (32)	0.517	147	195	194	223	250							
Cuminic Acid, (41)	0.525	136	180	185	221	246							
m-Hydroxybenzoic Acid, (50)	0.499	138	181	184	223	245							
o-Anisic Acid, (52)	0.499	138	206	193	221	243							
Ferulic Acid, (65)	0.508	139	209	206	224	246							
p-Coumaric Acid, (71)	0.510	136	199	205	223	258							
p-MCA, (72)	0.556	137	191	131	222	257							
Hesperetin, (18)	0.559	137	169	160	222								
p-HPAA, (82)	0.471	138	179	180	222								
p-MPLA, (85)	0.536	138	179	192	222								
Phloretic Acid, (87)	0.483	141	188	190	221								
Acacetin, (3)	0.564	135	180										
Vanillic Acid, (59)	0.504	136	192										
VAME, (60)	0.570	135	179										
Pyrocatechol, (95)	0.554	137	181										
Genkwanin, (7)	0.533	139											
Gentisic Acid, (45)	0.508	143											
Resorcylic Acid, (56)	0.504	144											
CADE, (62)	0.501	142											
CAPE, (63)	0.554	140											
m-Coumaric Acid, (67)	0.493	143											
Vanillin, (99)	0.548	136											

Legend: Rf2—retention factor in MPB, H° DEV 254 nm—hue equivalent at 254 nm prior to derivatisation, H° DEV 366 nm—hue equivalent at 366 nm prior to derivatisation, H° NP 366 nm—hue equivalent at 366 nm after derivatisation w/ NP-PEG-derivatisation reagent, Fl DEV λ—fluorescence λ max prior to derivatisation, Fl DEV λ m—fluorescence λ min prior to derivatisation, UV DEV λ_1-3_—UV-Vis λ max prior to derivatisation, Fl NP λ—fluorescence λ max after derivatisation with NP-PEG reagent, UV NP λ_1-3_—UV-Vis λ max after derivatisation with NP-PEG reagent. Note: coloured cells represent colours as seen on HPTLC plate.

**Table 9:** Results of compound matching for test compound B using DB 2B.

Name and Code	Rf 2	H° DEV 254 nm	H° DEV 366 nm	H° VSA 366 nm	H° T VSA	Fl DEV λ	Fl DEV λ m	UV DEV λ_1_	UV DEV λ_2_	UV DEV λ_3_	Fl VS λ	UV VS λ
Test Compound B	0.52	137	174	190	36	223	256	269	371	0	251	377
**Number of Potential Matches**	**34**	**34**	**31**	**30**	**30**	**30**	**26**	**16**	**2**	**2**	**2**	**2**
Isorhamnetin, (12)	0.536	136	180	191	43	224	255	261	364	0	252	388
Kaempferol, (14)	0.547	131	150	189	8	223	254	269	371	0	250	380
Acacetin, (3)	0.564	135	180	204	54	224	257	270				
Apigenin, (4)	0.507	134	199	206	46	224	256	271				
Genistein, (33)	0.549	128	181	211	34	224	253	260				
2,3,4-TMBA, (37)	0.479	142	120	241	22	225	254	261				
Benzoic Acid, (40)	0.57	136	185	199	48	211	241	276				
Eudesmic Acid, (43)	0.504	139	180	219	41	228	254	264				
Methyl Syringate, (48)	0.524	138	175	219	43	237	258	277				
Vanillic Acid, (59)	0.504	136	192	218	32	228	255	263				
VAME, (60)	0.57	135	179	207	71	225	254	263				
m-Coumaric Acid, (67)	0.493	143	215	208	49	224	252	280				
o-Coumaric Acid, (70)	0.519	146	205	223	41	224	258	279				
4-Methylpyrocatechol, (90)	0.546	137	180	242	345	223	256	283				
Pyrocatechol, (95)	0.554	137	181	219	36	226	265	277				
Dibenzyl Oxalate, (102)	0.531	139	199	245	62	222	257	262				
Formononetin, (32)	0.517	147	195	179	38	223	250					
Cuminic Acid, (41)	0.525	136	180	205	2	221	246					
Gentisic Acid, (45)	0.508	143	224	213	49	224	246					
m-HBA, (50)	0.499	138	181	232	38	223	245					
o-Anisic Acid, (52)	0.499	138	206	225	43	221	243					
p-HBA, (54)	0.513	130	184	212	28	225	252					
Resorcylic Acid, (56)	0.504	144	214	202	57	224	249					
Ferulic Acid, (65)	0.508	139	209	215	41	224	246					
p-Coumaric Acid, (71)	0.51	136	199	207	41	223	258					
p-MCA, (72)	0.556	137	191	245	10	222	257					
Hesperetin, (18)	0.559	137	169	202	20	222						
p-MPLA, (85)	0.536	138	179	228	38	222						
Phloretic Acid, (87)	0.483	141	188	229	33	221						
Vanillin, (99)	0.548	136	222	212	42	221						
p-HPAA, (82)	0.471	138	179									
Genkwanin, (7)	0.533	139										
CADE, (62)	0.501	142										
CAPE, (63)	0.5535	140										

Legend: Rf2—retention factor in MPB, H° DEV 254 nm—hue and colour equivalent at 254 nm prior to derivatisation, H° DEV 366 nm—hue and colour equivalent at 366 nm prior to derivatisation, H° VS 366 nm—hue and colour equivalent at 366 nm after derivatisation w/ VSA-derivatisation reagent, H° T VS—hue and colour equivalent at transmittance in white light after derivatisation w/ VSA-derivatisation reagent; Fl DEV λ max—fluorescence λ max prior to derivatisation, Fl DEV λ m—fluorescence λ min prior to derivatisation, UV DEV λ_1-3_—UV-Vis λ max prior to derivatisation, Fl VS λ—fluorescence λ max after derivatisation with VSA reagent, UV-Vis λ—UV-Vis λ max after derivatisation with VSA reagent. Note: coloured cells represent colours as seen on HPTLC plate.

**Table 10:** Summary of the correlations and % spectral matches used to determine the identity of test compound B.

UNK	DB	Rf	Name and Code	Rf	UV DEV	%	UV NP	%	UV VS	%	Match
B	1A, 1B	0.685	Isorhamnetin, Flavonol (12)	0.651	0.989	82.9	0.941	72.50	0.986	94.66	Kaemp-ferol
			Kaempferol, Flavonol (14)	0.654	0.996	100.0	0.986	100.0	0.997	100.0	
	2A, 2B	0.520	Isorhamnetin, Flavonol (12)	0.536	0.991	100.0	0.946	79.7	0.986	94.7	Kaemp-ferol
			Kaempferol, Flavonol (14)	0.547	0.991	100.0	0.994	100.0	0.997	100.0	
C	1A, 1B	0.685	Hesperetin, Flavanone (**18**)	0.667	0.251	5.5	0.076	31.0	0.275	45.6	None
			Naringenin, Flavanone (**20**)	0.680	0.229	6.6	0.281	27.8	0.203	35.9	
			Benzoic Acid, HBAD (**40**)	0.663	0.922	41.8	0.294	18.3	−0.08	59.1	
			m-Toluic Acid, HBAD (**51**)	0.681	0.920	42.9	−0.09	27.5	0.743	82.6	
			m-Coumaric Acid, HCAD (**67**)	0.633	0.814	24.2	0.239	27.0	−0.02	20.3	
			p-MPLA, HPLAD (**85**)	0.658	0.877	45.1	−0.17	20.6	0.494	79.7	
	2A, 2B	0.505	m-Coumaric Acid, HCAD (**67**)	0.493	0.224	35.8	0.213	26.2	−0.57	17.1	None
			p-Methoxy Phenyllactic Acid, HPLAD (**85**)	0.536	0.897	40.0	0.021	9.8	0.821	70.8	

**Table 11:** Results of Compound Matching for Test Compound C Using DB 1A.

Name and Code	Rf 1	H° DEV 254 nm	H° DEV 366 nm	H° NP 366 nm	Fl DEV λ	Fl DEV λ m	UV DEV λ_1_	UV DEV λ_2_	UV DEV λ_3_	Fl NP λ	UV NP λ_1_	UV NP λ_2_	UV NP λ_3_
Test Compound C	0.664	143	211	198	220	239	278	0	0	242	325	0	0
**Number of Potential Matches**	**57**	**57**	**51**	**40**	**40**	**33**	**16**	**13**	**13**	**11**	**11**	**11**	**11**
Hesperetin, (18)	0.667	137	169	160	222	238	290	0	0	249	339	0	0
Naringenin, (20)	0.680	137	177	160	223	246	292	0	0	249	333	0	0
Benzoic Acid, (40)	0.663	136	185	159	211	241	276	0	0	246	277	0	0
Eudesmic Acid, (43)	0.617	139	180	203	228	254	264	0	0	232	291	0	0
m-Toluic Acid, (51)	0.681	137	160	189	221	242	284	0	0	243	287	0	0
o-Toluic Acid, (53)	0.692	138	175	215	221	240	281	0	0	255	293	0	0
m-Coumaric Acid, (67)	0.633	143	215	220	224	252	280	0	0	228	288	0	0
HVA, (79)	0.654	137	177	213	222	240	282	0	0	244	288	0	0
p-HPAA, (82)	0.620	138	179	180	222	239	277	0	0	249	285	0	0
p-MPLA, (85)	0.658	138	179	192	222	236	276	0	0	250	284	0	0
Phloretic Acid, (87)	0.630	141	188	190	221	232	278	0	0	254	288	0	0
2-M-4-VPh, (88)	0.688	122	189	191	224	251	266	0	0				
p-Methoxyphenol, (89)	0.685	134	171	235	223	231	288	0	0				
Galangin, (11)	0.702	131	172	172	224	251	268						
VAME, (60)	0.658	135	179	231	225	254	263						
Vanillin, (99)	0.637	136	222	224	221	238	284						
Pinobanksin, (24)	0.697	137	191	174	224	239							
Formononetin, (32)	0.637	147	195	194	223	250							
Cuminic Acid, (41)	0.697	136	180	185	221	246							
Gentisic Acid, (45)	0.626	143	224	246	224	246							
Methyl Paraben, (47)	0.676	128	179	220	224	252							
m-HBA, (50)	0.644	138	181	184	223	245							
p-HBA, (54)	0.637	130	184	180	225	252							
Resorcylic Acid, (56)	0.630	144	214	226	224	249							
Salicylic Acid, (57)	0.679	140	211	181	223	242							
CAPE, (63)	0.676	140	235	209	225	250							
Ferulic Acid, (65)	0.616	139	209	206	224	246							
Methyl Ferulate, (68)	0.667	138	236	228	224	250							
Sinapic Acid, (74)	0.630	139	224	198	222	245							
Trans-p-CAME, (76)	0.674	136	193	202	222	237							
2-MBd, (96)	0.709	134	188	194	222	253							
2-Haph, (100)	0.704	133	152	180	228	254							
2-MHAPh, (101)	0.669	139	241	204	225	251							
Isorhamnetin, (12)	0.651	136	180	167	224								
Vanillic Acid, (59)	0.623	136	192	209	228								
o-Coumaric Acid, (70)	0.646	146	205	204	224								
p-Coumaric Acid, (71)	0.630	136	199	205	223								
t-Cinnamic Acid, (75)	0.672	140	242	222	229								
3-PPA, (86)	0.683	139	178	230	230								
4-MPCat, (90)	0.674	137	180	161	223								
Sakuranetin, (23)	0.699	137	160										
Acacetin, (3)	0.649	135	180										
Apigenin, (4)	0.617	134	199										
Chrysin, (6)	0.672	132	186										
Genistein, (33)	0.663	128	181										
Methyl-3,4,5-TMBz, (49)	0.670	141	186										
p-MCA, (72)	0.650	137	191										
Rosmarinic Acid, (73)	0.630	131	175										
Phenylacetic Acid, (81)	0.670	136	175										
Pyrocatechol, (95)	0.662	137	181										
p-Anisaldehyde, (97)	0.660	120	203										
Genkwanin, (7)	0.653	139											
Kaempferide, (13)	0.690	129											
Kaempferol, (14)	0.654	131											
Quercetin, (16)	0.617	126											
Biochanin A, (30)	0.692	133											
DL-p-HPLA, (84)	0.616	139											

Legend: Rf1—retention factor in MPA, H° DEV 254 nm—hue equivalent at 254 nm prior to derivatisation, H° DEV 366 nm—hue equivalent at 366 nm prior to derivatisation, H° NP 366 nm—hue equivalent at 366 nm after derivatisation w/ NP-PEG-derivatisation reagent, Fl DEV λ—fluorescence λ max prior to derivatisation, Fl DEV λ m—fluorescence λ min prior to derivatisation, UV DEV λ_1-3_—UV-Vis λ max prior to derivatisation, Fl NP λ— fluorescence λ max after derivatisation with NP-PEG reagent, UV NP λ_1-3_—UV-Vis λ max after derivatisation with NP-PEG reagent. Note: coloured cells represent colours as seen on HPTLC plate.

**Table 12:** Results of compound matching for test compound C using DB 1B.

Name and Code	Rf 1	H° DEV 254 nm	H° DEV 366 nm	H° VSA 366 nm	H° T VSA	Fl DEV λ	Fl DEV λ m	UV DEV λ_1_	UV DEV λ_2_	UV DEV λ_3_	Fl VS λ	UV VS λ
Test Compound C	0.635	140	183	204	14	221	239	278	0	0	246	349
**Number of Potential Matches**	**64**	**64**	**61**	**58**	**52**	**50**	**37**	**18**	**14**	**14**	**14**	**10**
Hesperetin, (18)	0.667	137	169	202	20	222	238	290	0	0	250	375
Naringenin, (20)	0.68	137	177	211	24	223	246	292	0	0	250	365
Taxifolin, (25)	0.591	135	183	206	24	224	240	292	0	0	251	363
Hesperetin, (18)	0.667	137	169	202	20	222	238	290	0	0	250	375
Naringenin, (20)	0.68	137	177	211	24	223	246	292	0	0	250	365
Taxifolin, (25)	0.591	135	183	206	24	224	240	292	0	0	251	363
Benzoic Acid, (40)	0.663	136	185	199	48	211	241	276	0	0	251	371
m-Toluic Acid, (51)	0.681	137	160	206	25	221	242	284	0	0	249	349
m-Coumaric Acid, (67)	0.633	143	215	208	49	224	252	280	0	0	252	344
p-MPLA, (85)	0.658	138	179	228	38	222	236	276	0	0	250	400
Eudesmic Acid, (43)	0.617	139	180	219	41	228	254	264	0	0	248	
HVA, (79)	0.654	137	177	230	67	222	240	282	0	0	248	
Phloretic Acid, (87)	0.630	141	188	229	33	221	232	278	0	0	249	
p-Methoxyphenol, (89)	0.685	134	171	227	37	223	231	288	0	0	249	
Kaempferol, (14)	0.654	131	150	189	8	223	254	269				
VAME, (60)	0.658	135	179	207	71	225	254	263				
Procatd, (98)	0.594	135	232	240	29	221	240	283				
Vanillin, (99)	0.637	136	222	212	42	221	238	284				
Myricetin, (15)	0.587	130	132	189	40	224	253					
Quercetin, (16)	0.617	126	127	192	41	224	254					
Daidzein, (31)	0.600	137	161	226	29	222	251					
Formononetin, (32)	0.637	147	195	179	38	223	250					
Genistein, (33)	0.663	128	181	211	34	224	253					
3,5-DHBA, (39)	0.594	138	191	222	33	225	251					
Gentisic Acid, (45)	0.626	143	224	213	49	224	246					
m-HBA, (50)	0.644	138	181	232	38	223	245					
o-Anisic Acid, (52)	0.589	138	206	225	43	221	243					
p-HBA, (54)	0.637	130	184	212	28	225	252					
Resorcylic Acid, (56)	0.630	144	214	202	57	224	249					
Salicylic Acid, (57)	0.679	140	211	205	48	223	242					
Caffeic Acid, (61)	0.589	139	211	215	38	224	250					
CAPE, (63)	0.676	140	235	240	27	225	250					
Ferulic Acid, (65)	0.616	139	209	215	41	224	246					
Rosmarinic Acid, (73)	0.630	131	175	226	43	224	248					
Sinapic Acid, (74)	0.630	139	224	224	46	222	245					
Trans-p-CAME, (76)	0.674	136	193	205	49	222	237					
2′-MAPh, (101)	0.669	139	241	188	0	225	251					
Acacetin, (3)	0.649	135	180	204	54	224						
Apigenin, (4)	0.617	134	199	206	46	224						
Chrysin, (6)	0.672	132	186	190	47	224						
Isorhamnetin, (12)	0.651	136	180	191	43	224						
2,3,4-THBA, (36)	0.589	138	185	226	21	223						
Methyl-3,4,5-TMBz, (49)	0.67	141	186	202	48	230						
Vanillic Acid, (59)	0.623	136	192	218	32	228						
o-Coumaric Acid, (70)	0.646	146	205	223	41	224						
p-Coumaric Acid, (71)	0.630	136	199	207	41	223						
p-MCA, (72)	0.65	137	191	245	10	222						
t-Cinnamic Acid, (75)	0.672	140	242	202	44	229						
Pyrogallol, (94)	0.602	137	186	224	21	224						
Pyrocatechol, (95)	0.662	137	181	219	36	226						
Methyl Syringate, (48)	0.610	138	175	219	43							
Phenylacetic Acid, (81)	0.67	136	175	242	23							
Methyl Paraben, (47)	0.676	128	179	228								
CADE, (62)	0.609	142	239	212								
Isoferulic Acid, (66)	0.600	140	227	215								
Methyl Ferulate, (68)	0.667	138	236	214								
3-PPA, (86)	0.683	139	178	231								
4-MPCat, (90)	0.674	137	180	242								
p-HPAA, (82)	0.620	138	179									
p-Anisaldehyde, (97)	0.66	120	203									
Abscisic Acid, (103)	0.598	127	171									
Genkwanin, (7)	0.653	139										
2,3,4-TMBA, (37)	0.602	142										
DL-p-HPLA, (84)	0.616	139										

Legend: Rf1—retention factor in MPA, H° DEV 254 nm—hue and colour equivalent at 254 nm prior to derivatisation, H° DEV 366 nm—hue and colour equivalent at 366 nm prior to derivatisation, H° VS 366 nm—hue and colour equivalent at 366 nm after derivatisation w/ VSA-derivatisation reagent, H° T VS—hue and colour equivalent at transmittance in white light after derivatisation w/ VSA-derivatisation reagent; Fl DEV λ max—fluorescence λ max prior to derivatisation, Fl DEV λ m—fluorescence λ min prior to derivatisation, UV DEV λ_1-3_—UV-Vis λ max prior to derivatisation, Fl VS λ—fluorescence λ max after derivatisation with VSA reagent, UV-Vis λ—UV-Vis λ max after derivatisation with VSA reagent. Note: coloured cells represent colours as seen on HPTLC plate.

**Table 13:** Results of compound matching for test compound C using DB 2A.

Name and Code	Rf 2	H° DEV 254 nm	H° DEV 366 nm	H° NP 366 nm	Fl DEV λ	Fl DEV λ m	UV DEV λ_1_	UV DEV λ_2_	UV DEV λ_3_	Fl NP λ	UV NP λ_1_	UV NP λ_2_	UV NP λ_3_
Test Compound C	0.505	140	180	193	220	239	278	0	0	242	325	0	0
**Number of Potential Matches**	**30**	**30**	**29**	**27**	**26**	**21**	**8**	**6**	**6**	**6**	**6**	**6**	**6**
Eudesmic Acid, (43)	0.504	139	180	203	228	254	264	0	0	232	291	0	0
m-Coumaric Acid, (67)	0.493	143	215	220	224	252	280	0	0	228	288	0	0
p-HPAA, (82)	0.471	138	179	180	222	239	277	0	0	249	285	0	0
DL-p-HPLA, (84)	0.466	139	120	171	223	238	278	0	0	243	285	0	0
p-MPLA, (85)	0.536	138	179	192	222	236	276	0	0	250	284	0	0
Phloretic Acid, (87)	0.483	141	188	190	221	232	278	0	0	254	288	0	0
Kaempferol, (14)	0.547	131	150	150	223	254	269						
Vanillin, (99)	0.548	136	222	224	221	238	284						
Formononetin, (32)	0.517	147	195	194	223	250							
Genistein, (33)	0.549	128	181	136	224	253							
2,3,4-TMBA, (37)	0.479	142	120	147	225	254							
Cuminic Acid, (41)	0.525	136	180	185	221	246							
Gentisic Acid, (45)	0.508	143	224	246	224	246							
m-HBA, (50)	0.499	138	181	184	223	245							
o-Anisic Acid, (52)	0.499	138	206	193	221	243							
p-HBA, (54)	0.513	130	184	180	225	252							
Resorcylic Acid, (56)	0.504	144	214	226	224	249							
CADE, (62)	0.501	142	239	223	223	247							
CAPE, (63)	0.554	140	235	209	225	250							
Ferulic Acid, (65)	0.508	139	209	206	224	246							
Isoferulic Acid, (66)	0.459	140	227	216	224	248							
Isorhamnetin, (12)	0.536	136	180	167	224								
Vanillic Acid, (59)	0.504	136	192	209	228								
o-Coumaric Acid, (70)	0.519	146	205	204	224								
p-Coumaric Acid, (71)	0.51	136	199	205	223								
4-MPCAt, (90)	0.546	137	180	161	223								
Methyl Syringate, (48)	0.524	138	175	203									
Apigenin, (4)	0.507	134	199										
Pyrocatechol, (95)	0.554	137	181										
Genkwanin, (7)	0.533	139											

Legend: Rf2—retention factor in MPB, H° DEV 254 nm—hue equivalent at 254 nm prior to derivatisation, H° DEV 366 nm—hue equivalent at 366 nm prior to derivatisation, H° NP 366 nm—hue equivalent at 366 nm after derivatisation w/ NP-PEG-derivatisation reagent, Fl DEV λ—fluorescence λ max prior to derivatisation, Fl DEV λ m—fluorescence λ min prior to derivatisation, UV DEV λ_1-3_—UV-Vis λ max prior to derivatisation, Fl NP λ—fluorescence λ max after derivatisation with NP-PEG reagent, UV NP λ_1-3_—UV-Vis λ max after derivatisation with NP-PEG reagent. Note: coloured cells represent colours as seen on HPTLC plate.

**Table 14:** Results of compound matching for test compound C using DB 2B.

Name and Code	Rf 2	H° DEV 254 nm	H° DEV 366 nm	H° VSA 366 nm	H° T VSA	Fl DEV λ	Fl DEV λ m	UV DEV λ1	UV DEV λ2	UV DEV λ3	Fl VS λ	UV VS λ
Test Compound C	0.505	140	183	204	14	221	239	278	0	0	246	349
**Number of Potential Matches**	**15**	**15**	**12**	**11**	**8**	**7**	**0**	**0**	**0**	**0**	**0**	**0**
m-Coumaric Acid, (67)	0.493	143	215	208	49	224	252	280	0	0	252	344
p-MPLA, (85)	0.536	138	179	228	38	222	236	276	0	0	250	400
Eudesmic Acid, (43)	0.504	139	180	219	41	228	254	264	0	0	248	
Phloretic Acid, (87)	0.483	141	188	229	33	221	232	278	0	0	249	
Kaempferol, (14)	0.547	131	150	189	8	223	254	269				
Vanillin, (99)	0.548	136	222	212	42	221	238	284				
Formononetin, (32)	0.517	147	195	179	38	223	250					
Genistein, (33)	0.549	128	181	211	34	224	253					
Cuminic Acid, (41)	0.525	136	180	205	2	221	246					
Gentisic Acid, (45)	0.508	143	224	213	49	224	246					
m-HBA, (50)	0.499	138	181	232	38	223	245					
o-Anisic Acid, (52)	0.499	138	206	225	43	221	243					
p-HBA, (54)	0.513	130	184	212	28	225	252					
Resorcylic Acid, (56)	0.504	144	214	202	57	224	249					
CAPE, (63)	0.554	140	235	240	27	225	250					
Ferulic Acid, (65)	0.508	139	209	215	41	224	246					
Apigenin, (4)	0.507	134	199	206	46	224						
Isorhamnetin, (12)	0.536	136	180	191	43	224						
Vanillic Acid, (59)	0.504	136	192	218	32	228						
o-Coumaric Acid, (70)	0.519	146	205	223	41	224						
p-Coumaric Acid, (71)	0.51	136	199	207	41	223						
Pyrocatechol, (95)	0.554	137	181	219	36	226						
Dibenzyl Oxalate, (102)	0.531	139	199	245	62	222						
Methyl Syringate, (48)	0.524	138	175	219	43							
CADE, (62)	0.501	142	239	212								
Isoferulic Acid, (66)	0.459	140	227	215								
4-MPCat, (90)	0.546	137	180	242								
p-HPAA, (82)	0.471	138	179									
Genkwanin, (7)	0.533	139										
2,3,4-TMBA, (37)	0.479	142										
DL-p-HPLA, (84)	0.466	139										

Legend: Rf2—retention factor in MPB, H° DEV 254 nm—hue and colour equivalent at 254 nm prior to derivatisation, H° DEV 366 nm—hue and colour equivalent at 366 nm prior to derivatisation, H° VS 366 nm—hue and colour equivalent at 366 nm after derivatisation w/ VSA-derivatisation reagent, H° T VS—hue and colour equivalent at transmittance in white light after derivatisation w/ VSA-derivatisation reagent; Fl DEV λ max—fluorescence λ max prior to derivatisation, Fl DEV λ m—fluorescence λ min prior to derivatisation, UV DEV λ_1-3_—UV-Vis λ max prior to derivatisation, Fl VS λ—fluorescence λ max after derivatisation with VSA reagent, UV-Vis λ—UV-Vis λ max after derivatisation with VSA reagent. Note: coloured cells represent colours as seen on HPTLC plate.

**Table 15:** Consolidation of match compounds for test compound C.

Unknown	Rf	DB	Name	Rf	Match	Rf
Test Compound C	0.664	1A	hesperetin, (18)	0.667	hesperetin, (18)	0.667
			naringenin, (20)	0.680	naringenin, (20)	0.680
			benzoic acid, (40)	0.663	benzoic acid, (40)	0.663
			eudesmic acid, (43)	0.617	m-toluic acid, (51)	0.681
			m-toluic acid, (51)	0.681	m-coumaric acid, (67)	0.633
			o-toluic acid, (53)	0.692	p-MPLA, (85)	0.658
			m-coumaric acid, (67)	0.633		
			HVA, (79)	0.654		
			p-HPAA, (82)	0.620		
			p-MPLA, (85)	0.658		
		1B	hesperetin, (18)	0.667		
			naringenin, (20)	0.680		
			taxifolin, (25)	0.591		
			benzoic acid, (40)	0.663		
			m-toluic acid, (51)	0.681		
			m-coumaric acid, (67)	0.633		
			p-MPLA, (85)	0.658		
	0.505	2A	eudesmic acid, (43)	0.504	m-coumaric acid, (67)	0.493
			m-coumaric acid, (67)	0.493	p-MPLA, (85)	0.536
			p-HPAA, (82)	0.471		
			DL-p-HPLA, (84)	0.466		
			p-MPLA, (85)	0.536		
			phloretic acid, (87)	0.483		
		2B	m-coumaric acid, (67)	0.493		
			p-MPLA, (85)	0.536		

**Table 16:** Summary of the correlations and % spectra matches used to determine the identity of the unknown bands for Manuka honey.

Database	UNK	Rf	Name, Class and Code	Rf	UV DEV	%	UV NP	%	UV VS	%	Match
1A and 1B	1	0.020	leptosperine, HCAD (46)	0.014	0.863	22.4	−0.013	49.5	0.923	78.3	leptosperine
	2	0.077	-	-	-	-	-	-	-	-	none
	3	0.134	-	-	-	-	-	-	-	-	none
	4	0.199	mandelic acid, HPAAD (80)	0.162	0.696	65.9	0.882	43.4	0.911	75.1	mandelic acid
	5	0.240	kojic acid, non-phenolic (105)	0.287	0.960	26.4	0.518	31.1	0.847	26.0	kojic acid
	6	0.319	lepteridine, non-phenolic (106)	0.314	0.924	44.3	0.902	50.4	0.526	47.0	lepteridine
	7	0.392	EGCG, flavan-3-ol (29)	0.407	0.834	82.0	0.479	39.4	0.837	55.5	EGCG
	8	0.467	lumichrome, non-phenolic (107)	0.464	0.319	25.1	0.481	62.9	0.231	27.4	lumichrome
	9	0.513	-	-	-	-	-	-	-	-	None
	10	0.543	-	-	-	-	-	-	-	-	None
	11	0.635	methyl syringate, HBAD (48)	0.610	0.983	48.4	0.944	70.5	0.907	77.6	methyl syringate
	12	0.685	benzoic acid, HBAD (40)	0.663	0.894	30.3	0.043	15.9	0.413	17.1	none
2A and 2B	**1**	0.021	-	-	-	-	-	-	-	-	none
	**2**	0.081	-	-	-	-	-	-	-	-	none
	**3**	0.098	-	-	-	-	-	-	-	-	none
	**4**	0.121	-	-	-	-	-	-	-	-	none
	**5**	0.150	kojic acid, non-phenolic (105)	0.171	0.939	30.2	0.914	61.1	0.674	21.4	kojic acid
	**6**	0.220	lepteridine, non-phenolic (106)	0.217	0.514	25.4	0.947	33.6	0.674	43.1	lepteridine
	**7**	0.310	gallic acid, HBAD (44)	0.321	0.969	84.1	0.894	73.3	0.912	87.9	gallic acid
	**8**	0.349	mandelic acid, HPAAD (80)	0.347	0.438	36.4	0.027	22.9	0.892	68.3	mandelic acid
	**9**	0.425	2,3,4-THBA, HBAD (36)	0.437	0.828	31.8	0.963	86.8	0.753	51.2	2,3,4-THBA
	**10**	0.470	m-HBA, HBAD (**50**)	0.499	0.904	37.4	0.961	27.5	−0.518	20.3	o-anisic acid
			o-anisic acid, HBAD (**52**)	0.499	0.888	29.0	0.897	30.8	0.888	44.1	
			homovanillic acid, HPAAD (**79**)	0.441	0.529	19.8	0.293	25.3	0.882	36.7	
	**11**	0.513	methyl syringate, HBAD (**48**)	0.524	0.992	56.0	0.979	100.0	0.930	85.1	methyl syringate
			m-coumaric acid, HCAD (**67**)	0.493	0.914	53.8	0.933	38.5	0.979	84.3	
	**12**	0.603	naringenin, flavanone (**20**)	0.591	0.760	24.0	0.655	35.9	0.782	35.9	salicylic acid
			pinobanksin, flavanonol (**24**)	0.598	0.776	22.0	0.723	27.9	0.607	65.5	
			salicylic acid, HBAD (**57**)	0.582	0.771	15.9	0.909	13.1	0.545	75.1	

## Data Availability

No new data were created or analysed in this study. Data sharing is not applicable to this article.

## Supplementary Materials

The following are available online at https://www.mdpi.com/article/10.3390/molecules27196651/s1, **Figure S1**: Basic Flavone Structure, **Figure S2**: Basic Flavonol Structure, **Figure S3**: Basic Flavanone Structure, **Figure S4**: Basic Flavanonol Structure, **Figure S5**: Basic Flavan-3-ol Structure, **Figure S6**: Basic Isoflavonoid Structure, **Figure S7**: Chalcone, **Figure S8**: Hydroxybenzoic Acid and its Derivatives (HBADs), **Figure S9**: Ellagic Acid (42), **Figure S10**: Hydroxycinnamic Acid and its Derivatives (HCADs), **Figure S11**: Hydroxyphenylacetic Acid and Derivatives (HPAAD), **Figure S12**: Hydroxyphenyllactic Acid and Derivatives (HPLAD) and Hydroxyphenylpropanoic Acid and Derivatives (HPPAD), **Figure S13**: Other phenolic compounds, **Figure S14**: Dibenzyl oxalate, **Figure S15**: Structure of the non-phenolic compounds, **Figure S16**: (A-C) UV-Vis spectra (prior to derivatisation) overlay of unknown B vs. isorhamnetin and kaempferol (A) and UV-Vis (prior to derivatisation) spectra overlay of unknown A vs. the ±0.125 AU of isorhamnetin (B) and vs. ±0.125 AU of kaempferol (C), **Figure S17**: (A-C). UV-Vis spectra (after derivatisation with NP-PEG reagent) overlay of unknown B vs. isorhamnetin and kaempferol (A) and UV-Vis (after derivatisation with NP-PEG reagent) spectra overlay of unknown A vs. the ±0.125 AU of isorhamnetin (B) and vs. ±0.125 AU of kaempferol (C), **Figure S18**: (A-C). UV-Vis spectra (after derivatised with VSA reagent) overlay of unknown B vs. isorhamnetin and kaempferol (A) and UV-Vis (after derivatised with VSA reagent) spectra overlay of unknown A vs. the ±0.125 AU of isorhamnetin (B) and vs. ±0.125 AU of kaempferol (C), **Figure S19**: (A-G). UV-Vis spectra (prior to derivatisation) overlay of unknown C vs. the match compounds and UV-Vis (prior to derivatisation) spectra overlay of unknown A vs. the ±0.125 AU of hesperitin (B), vs. ±0.125 AU of naringenin (C), vs. ±0.125 AU of benzoic acid (D), ±0.125 AU of m-toluic acid (E), ±0.125 AU of m-coumaric acid (F), and ±0.125 AU of p-MPLA (G), **Figure S20**: (A-D). UV-Vis spectra (after derivatisation with NP-PEG reagent) overlay of unknown C vs. benzoic acid (B), m-toluic acid (C), and 4-MPLA (D) and UV-Vis (after development) spectra overlay of unknown A vs. the ±0.125 AU of benzoic acid (B), ±0.125 AU of m-toluic acid (C), and ±0.125 AU of p-MPLA (D), **Table S1**: Flavones Standards Used in the Database Development, **Table S2**: Flavonol Standards Used in the Database Development, **Table S3**: Flavanone Standards Used in the Database Development, **Table S4**: Flavanone Standards Used in the Database Development, **Table S5**: Flavan-3-ol Standards Used in the Database Development, **Table S6**: Isoflavonoid Standards used in the Database Development, **Table S7**: t-Chalcone Standard Used in the Database Development, **Table S8**: Hydroxybenzoic Acid and its Derivatives (HBADs) Standards used in the Database Development, **Table S9**: Hydroxycinnamic Acid and its Derivatives (HCADs) Standards used in the Database Development, **Table S10**: Hydroxyphenylacetic Acid and Derivatives (HPAAD) Standards used in the Database Development, **Table S11**: Hydroxyphenyllactic Acid and Derivatives (HPLAD) and Hydroxyphenylpropanoic Acid and Derivatives (HPPAD) Standards used in the Database Development, **Table S12**: Other/Miscellaneous Phenolic Standards used in the Database Development, **Table S13**: Oxalate ester Standard used in the Database Development, **Table S14**: Non-phenolic compounds used in the Database Development, **Table S15**: Summary of the data used to determine the identity of the unknown bands in Manuka honey (Database 1A), **Table S16**: Summary of the data used to determine the identity of the unknown bands in Manuka honey (Database 1B), **Table S17**: Summary of the data used to determine the identity of the unknown bands in Manuka honey (Database 2A), **Table S18**: Summary of the data used to determine the identity of the unknown bands in Manuka honey (Database 2B).

## Sample Availability

Samples of the compounds and honeys are available from the authors.

## References

[1] Badjah Hadj Ahmed A.Y., Wabaidur S.M., Siddiqui M.R., Alothman Z.A., Obeid M.S., Khan M.R., Al-Tamrah S.A. (2016). Simultaneous determination of twenty-five polyphenols in multifloral and cactus honeys using solid-phase extraction and high-performance liquid chromatography with photodiode array detection. Eur. Food Res. Technol..

[2] Lamien-Meda A., Lamien C.E., Millogo J.F., Romito M., Nacoulma O.G. (2005). Physicochemical analyses of Burkina Fasan honey. Acta Vet. Brno.

[3] Zhao L., Ren C., Xue X., Lu H., Wang K., Wu L. (2022). Safflomin A: A novel chemical marker for *Carthamus tinctorius* L. (Safflower) monofloral honey. Food Chem..

[4] Das A., Datta S., Mukherjee S., Bose S., Ghosh S., Dhar P. (2015). Evaluation of antioxidative, antibacterial and probiotic growth stimulatory activities of *Sesamum indicum* honey containing phenolic compounds and lignans. LWT—Food Sci. Technol..

[5] Guzelmeric E., Ciftci I., Yuksel P.I., Yesilada E. (2020). Importance of chromatographic and spectrophotometric methods in determining authenticity, classification and bioactivity of honey. LWT.

[6] Nascimento K.S.D., Gasparotto Sattler J.A., Lauer Macedo L.F., Serna González C.V., Pereira de Melo I.L., da Silva Araújo E., Granato D., Sattler A., de Almeida-Muradian L.B. (2018). Phenolic compounds, antioxidant capacity and physicochemical properties of Brazilian *Apis mellifera* honeys. LWT—Food Sci. Technol..

[7] Nguyen H.T.L., Panyoyai N., Kasapis S., Pang E., Mantri N. (2019). Honey and Its Role in Relieving Multiple Facets of Atherosclerosis. Nutrients.

[8] Stanek N., Teper D., Kafarski P., Jasicka-Misiak I. (2019). Authentication of phacelia honeys (*Phacelia tanacetifolia*) based on a combination of HPLC and HPTLC analyses as well as spectrophotometric measurements. LWT—Food Sci. Technol..

[9] Sant’ana L.D., Buarque Ferreira A.B., Lorenzon M.C.A., Berbara R.L.L., Castro R.N. (2014). Correlation of Total Phenolic and Flavonoid Contents of Brazilian Honeys with Colour and Antioxidant Capacity. Int. J. Food Prop..

[10] Michalkiewicz A., Biesaga M., Pyrzynska K. (2008). Solid-phase extraction procedure for determination of phenolic acids and some flavonols in honey. J. Chromatogr. A.

[11] Gheldof N., Wang X.-H., Engeseth N.J. (2002). Identification and Quantification of Antioxidant Components of Honeys from Various Floral Sources. J. Agric. Food Chem..

[12] Demir Kanbur E., Yuksek T., Atamov V., Ozcelik A.E. (2021). A comparison of the physicochemical properties of chestnut and highland honey: The case of Senoz Valley in the Rize province of Turkey. Food Chem..

[13] Martos I., Ferreres F., Tomás-Barberán F.A. (2000). Identification of flavonoid markers for the botanical origin of Eucalyptus honey. J. Agric. Food Chem..

[14] Tomás-Barberán F.A., Martos I., Ferreres F., Radovic B.S., Anklam E. (2001). HPLC flavonoid profiles as markers for the botanical origin of European unifloral honeys. J. Sci. Food Agric..

[15] Escriche I., Kadar M., Juan-Borrás M., Domenech E. (2014). Suitability of antioxidant capacity, flavonoids and phenolic acids for floral authentication of honey. Impact of industrial thermal treatment. Food Chem..

[16] Lawag I.L., Lim L.-Y., Joshi R., Hammer K.A., Locher C. (2022). A Comprehensive Survey of Phenolic Constituents Reported in Monofloral Honeys around the Globe. Foods.

[17] Trifković J., Andrić F., Ristivojević P., Guzelmeric E., Yesilada E. (2017). Analytical methods in tracing honey authenticity. J. AOAC Int..

[18] Green K.J., Lawag I.L., Locher C., Hammer K.A. (2022). Correlation of the antibacterial activity of commercial manuka and *Leptospermum* honeys from Australia and New Zealand with methylglyoxal content and other physicochemical characteristics. PLoS ONE.

[19] Rückriemen J., Henle T. (2018). Pilot study on the discrimination of commercial *Leptospermum* honeys from New Zealand and Australia by HPLC–MS/MS analysis. Eur. Food Res. Technol..

[20] Stanek N., Jasicka-Misiak I. (2018). HPTLC Phenolic Profiles as Useful Tools for the Authentication of Honey. Food Anal. Methods.

[21] Cheung Y., Meenu M., Yu X., Xu B. (2019). Phenolic acids and flavonoids profiles of commercial honey from different floral sources and geographic sources. Int. J. Food Prop..

[22] Deng J., Liu R., Lu Q., Hao P., Xu A., Zhang J., Tan J. (2018). Biochemical properties, antibacterial and cellular antioxidant activities of buckwheat honey in comparison to manuka honey. Food Chem..

[23] Habib H.M., Al Meqbali F.T., Kamal H., Souka U.D., Ibrahim W.H. (2014). Physicochemical and biochemical properties of honeys from arid regions. Food Chem..

[24] Hempattarasuwan P., Settachaimongkon S., Duangmal K. (2019). Impact of botanical source and processing conditions on physicochemical properties and antioxidant activity of honey in the northern part of Thailand. Int. J. Food Sci. Technol..

[25] Combarros-Fuertes P., Estevinho L.M., Dias L.G., Castro J.M., Tomás-Barberán F.A., Tornadijo M.E., Fresno-Baro J.M. (2019). Bioactive Components and Antioxidant and Antibacterial Activities of Different Varieties of Honey: A Screening Prior to Clinical Application. J. Agric. Food Chem..

[26] Bong J., Loomes K.M., Lin B., Stephens J.M. (2018). New approach: Chemical and fluorescence profiling of NZ honeys. Food Chem..

[27] Lin B., Daniels B.J., Middleditch M.J., Furkert D.P., Brimble M.A., Bong J., Stephens J.M., Loomes K.M. (2020). Utility of the *Leptospermum scoparium* Compound Lepteridine as a Chemical Marker for Manuka Honey Authenticity. ACS Omega.

[28] Weston R.J., Brocklebank L.K., Lu Y. (2000). Identification and quantitative levels of antibacterial components of some New Zealand honeys. Food Chem..

[29] Oelschlaegel S., Gruner M., Wang P.-N., Boettcher A., Koelling-Speer I., Speer K. (2012). Classification and characterization of manuka honeys based on phenolic compounds and methylglyoxal. J. Agric. Food Chem..

[30] Yao L., Bhandari B.R., Datta N., Singanusong R., D’Arcy B.R. (2003). Crystallisation and moisture sorption properties of selected Australian unifloral honeys. J. Sci. Food Agric..

[31] Anand S., Deighton M., Livanos G., Morrison P.D., Pang E.C.K., Mantri N. (2019). Antimicrobial Activity of Agastache Honey and Characterization of Its Bioactive Compounds in Comparison with Important Commercial Honeys. Front. Microbiol..

[32] Shen S., Wang J., Zhuo Q., Chen X., Liu T., Zhang S.-Q. (2018). Quantitative and discriminative evaluation of contents of phenolic and flavonoid and antioxidant competence for Chinese honeys from different botanical origins. Molecules.

[33] Stephens J.M., Schlothauer R.C., Morris B.D., Yang D., Fearnley L., Greenwood D.R., Loomes K.M. (2010). Phenolic compounds and methylglyoxal in some New Zealand manuka and kanuka honeys. Food Chem..

[34] Marshall S.M., Schneider K.R., Cisneros K.V., Gu L. (2014). Determination of antioxidant capacities, α-dicarbonyls, and phenolic phytochemicals in florida varietal honeys using HPLC-DAD-ESI-MSn. J. Agric. Food Chem..

[35] Stephens J.M., Loomes K.M., Braggins T.J., Bong J., Lin B., Prijic G. (2017). Fluorescence: A Novel Method for Determining Manuka Honey Floral Purity. Honey Analysis.

[36] Islam M.K., Sostaric T., Lim L.Y., Hammer K., Locher C. (2021). Development of an HPTLC-based dynamic reference standard for the analysis of complex natural products using Jarrah honey as test sample. PLoS ONE.

[37] Islam M.K., Lawag I.L., Green K.J., Sostaric T., Hammer K.A., Lim L.Y., Locher C. (2022). An investigation of the suitability of melissopalynology to authenticate Jarrah honey. Curr. Res. Food Sci..

[38] Green K.J., Islam M.K., Lawag I., Locher C., Hammer K.A. (2022). Honeys derived from plants of the coastal sandplains of Western Australia: Antibacterial and antioxidant activity, and other characteristics. J. Apic. Res..

[39] Matteini P., Agati G., Pinelli P., Goti A. (2011). Modes of complexation of rutin with the flavonoid reagent diphenylborinic acid 2-aminoethyl ester. Mon. Chem.—Chem. Mon..

[40] Bernardi T., Bortolini O., Massi A., Sacchetti G., Tacchini M., De Risi C. (2019). Exploring the Synergy Between HPTLC and HPLC-DAD for the Investigation of Wine-Making By-Products. Molecules.

[41] Eike Reich A.S. (2006). High-Performance Thin-Layer Chromatography for the Analysis of Medicinal Plants.

[42] Lin B., Loomes K.M., Prijic G., Schlothauer R., Stephens J.M. (2017). Lepteridine as a unique fluorescent marker for the authentication of manuka honey. Food Chem..

[43] Afrin S., Giampieri F., Forbes-Hernández T.Y., Gasparrini M., Amici A., Cianciosi D., Quiles J.L., Battino M. (2018). Manuka honey synergistically enhances the chemopreventive effect of 5-fluorouracil on human colon cancer cells by inducing oxidative stress and apoptosis, altering metabolic phenotypes and suppressing metastasis ability. Free Radic. Biol. Med..

[44] Beitlich N., Koelling-Speer I., Oelschlaegel S., Speer K. (2014). Differentiation of manuka honey from kanuka honey and from jelly bush honey using HS-SPME-GC/MS and UHPLC-PDA-MS/MS. J. Agric. Food Chem..

[45] Bong J., Loomes K.M., Schlothauer R.C., Stephens J.M. (2016). Fluorescence markers in some New Zealand honeys. Food Chem..

[46] Shen S., Wang J., Chen X., Liu T., Zhuo Q., Zhang S.-Q. (2019). Evaluation of cellular antioxidant components of honeys using UPLC-MS/MS and HPLC-FLD based on the quantitative composition-activity relationship. Food Chem..

[47] Afrin S., Giampieri F., Gasparrini M., Forbes-Hernández T.Y., Cianciosi D., Reboredo-Rodriguez P., Manna P.P., Zhang J., Quiles J.L., Battino M. (2018). The inhibitory effect of manuka honey on human colon cancer HCT-116 and LoVo cell growth. Part 2: Induction of oxidative stress, alteration of mitochondrial respiration and glycolysis, and suppression of metastatic ability. Food Funct..

[48] Khalil M.I., Alam N., Moniruzzaman M., Sulaiman S.A., Gan S.H. (2011). Phenolic Acid Composition and Antioxidant Properties of Malaysian Honeys. J. Food Sci..

[49] Yao L., Datta N., Tomás-Barberán F.A., Ferreres F., Martos I., Singanusong R. (2003). Flavonoids, phenolic acids and abscisic acid in Australian and New Zealand *Leptospermum* honeys. Food Chem..

[50] Stanek N., Kafarski P., Jasicka-Misiak I.J. (2019). Development of a high performance thin layer chromatography method for the rapid qualification and quantification of phenolic compounds and abscisic acid in honeys. J. Chromatogr. A.

[51] Islam M.K., Sostaric T., Lim L.Y., Hammer K., Locher C. (2021). Antioxidant HPTLC-DPPH Fingerprinting of Honeys and Tracking of Antioxidant Constituents Upon Thermal Exposure. Foods.

[52] Jasicka-Misiak I., Makowicz E., Stanek N. (2018). Chromatographic fingerprint, antioxidant activity, and colour characteristic of polish goldenrod (*Solidago virgaurea* L.) honey and flower. Eur. Food Res. Technol..

[53] Islam M.K., Sostaric T., Lim L.Y., Hammer K., Locher C. (2020). Development and validation of an HPTLC–DPPH assay and its application to the analysis of honey. JPC—J. Planar Chromatogr.—Mod. TLC.

[54] Guzelmeric E., Ristivojević P., Trifković J., Dastan T., Yilmaz O., Cengiz O., Yesilada E. (2018). Authentication of Turkish propolis through HPTLC fingerprints combined with multivariate analysis and palynological data and their comparative antioxidant activity. LWT.

[55] Guzelmeric E., Vovk I., Yesilada E. (2015). Development and validation of an HPTLC method for apigenin 7-O-glucoside in chamomile flowers and its application for fingerprint discrimination of chamomile-like materials. J. Pharm. Biomed. Anal..

[56] Rusko J., Vainovska P., Vilne B., Bartkevics V. (2021). Phenolic profiles of raw mono- and polyfloral honeys from Latvia. J. Food Compost. Anal..

[57] Wu A.H., French D. (2013). Implementation of liquid chromatography/mass spectrometry into the clinical laboratory. Clin. Chim. Acta.

[58] Pascual-Maté A., Osés S.M., Fernández-Muiño M.A., Sancho M.T. (2017). Analysis of Polyphenols in Honey: Extraction, Separation and Quantification Procedures. Sep. Purif. Rev..

[59] Sik B., Székelyhidi R., Lakatos E., Kapcsándi V., Ajtony Z. (2022). Analytical procedures for determination of phenolics active herbal ingredients in fortified functional foods: An overview. Eur. Food Res. Technol..

[60] Neveu V., Perez-Jiménez J., Vos F., Crespy V., du Chaffaut L., Mennen L., Knox C., Eisner R., Cruz J., Wishart D. (2010). Phenol-Explorer: An online comprehensive database on polyphenol contents in foods. Database.

[61] Ferreira J.F., Luthria D.L., Sasaki T., Heyerick A. (2010). Flavonoids from *Artemisia annua* L. as antioxidants and their potential synergism with artemisinin against malaria and cancer. Molecules.

[62] Jan S., Abbas N., Jan S., Abbas N. (2018). Chapter 4—Chemistry of Himalayan Phytochemicals. Himalayan Phytochemicals.

[63] Gafner S., Bergeron C., Batcha L.L., Angerhofer C.K., Sudberg S., Sudberg É.M., Guinaudeau H., Gauthier R. (2003). Analysis of *Scutellaria lateriflora* and Its Adulterants *Teucrium canadense* and *Teucrium chamaedrys* by LC–UV/MS, TLC, and Digital Photomicroscopy. J. AOAC Int..

[64] Locher C., Tang E., Neumann J., Sostaric T. (2018). High-performance thin-layer chromatography profiling of Jarrah and Manuka honeys. JPC—J. Planar Chromatogr.—Mod. TLC.

[65] Lawag I.L., Yoo O., Lim L.Y., Hammer K., Locher C. (2021). Optimisation of Bee Pollen Extraction to Maximise Extractable Antioxidant Constituents. Antioxidants.

[66] Islam M.K., Vinsen K., Sostaric T., Lim L.Y., Locher C. (2021). Detection of syrup adulterants in manuka and jarrah honey using HPTLC-multivariate data analysis. PeerJ.

[67] Jesionek W., Majer-Dziedzic B., Choma I.M. (2015). Separation, Identification, and Investigation of Antioxidant Ability of Plant Extract Components Using TLC, LC–MS, and TLC–DPPH. J. Liq. Chromatogr. Relat. Technol..

