# Supplementary material for: The Development and Application of a HPTLC-Derived Database for the Identification of Phenolics in Honey
Source: Molecules. 2022 Oct 6;27(19):6651. doi: 10.3390/molecules27196651 (PMC9572973; doi:10.3390/molecules27196651)
Supplement: Supplementary file 1 [file molecules-27-06651-s001.zip › molecules-1898606-supplementary.pdf]

Standards Used in the Database Development

In summary, 107 standards, 102 of which were phenolic in nature, were utilised in the database development (Supplementary Material, Tables 1-4). Thirty-five of the standards were flavonoids, 9 of which were from the flavone sub-class, 8 were flavonols, 6 were flavanones, 2 were flavanonols, 4 were flavan-3-ols, 5 were flavones and one was a chalcone. Forty-one standards could be classified as simple phenolic, 25 of which belonged to the hydroxybenzoic acid and derivatives (HBAD) subclass, while 16 belonged to the hydroxycinnamic acid and derivatives (HCAD) subclass. Twenty-seven standards were other/miscellaneous phenolics, 6 of which belonged to the hydroxyphenylacetic acid and derivatives (HPAAD) subclass, 3 tohydroxyphenyllactic acid and derivatives (HPLAD), 2 to hydroxyphenylpropanoic acid and derivatives (HPPAD), 2 were alkylmethoxyphenols (AMPh), 3 were alkylphenols (APh), 1 was an aminophenol, two were simple phenols, 4 were hydroxybenzaldehydes (HBzd), 2 were hydroxyacetophenones (HAPh) and 1 was an oxalate ester. There were also four non phenolic compounds that were used in the study.

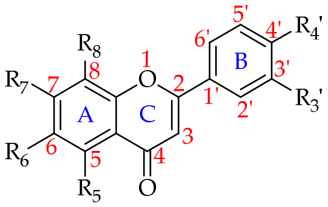

Supplementary Figure S1: Basic Flavone Structure (See **1-9** in **Supplementary Table S1**)

Supplementary Table S1: Flavones Standards Used in the Database Development (See **Supplementary Figure S1** for general structure)

R5	R6	R7	R8	R3′	R4′	Code	Name	CAS No.	Supplier	Concentration (mg/mL) & Injection Volume (μL)
-O-H	-H	-H	-H	-H	-H	**1**	5-Methoxyflavone	42079-78-7	A	0.5, 5
-H	-O-Glc	-H	-H	-H	-H	**2**	6-Hydroxy-flavone-β-D-Glc	906-33-2	A	0.5, 5
-O-H	-H	-O-Me	-H	-H	-O-Me	**3**	Acacetin	480-44-4	B	0.5, 5
-O-H	-H	-O-H	-H	-H	-O-H	**4**	Apigenin	520-36-5	C	0.5, 5
-O-H	-H	-O-Glc	-H	-H	-H	**5**	Baicalin	21967-41-9	C	1.0, 5
-O-H	-H	-O-H	-H	-H	-H	**6**	Chrysin	480-40-0	C	0.5, 5
-O-H	-H	-O-Me	-H	-H	-O-H	**7**	Genkwanin	437-64-9	B	0.5, 5
-O-H	-H	-O-H	-H	-O-H	-O-H	**8**	Luteolin	491-70-3	C	0.5, 5
-O-H	-H	-O-H	-Glc	-H	-O-H	**9**	Vitexin	3681-93-4	B	0.5, 5

Legend: -H – hydride, -O-H – hydroxide, -O-Me – Methoxide, - Glc – glucoside, Suppliers: A=Sigma Aldrich (Castle Hill, NSW, Australia), B=Angene International Ltd. (Nanjing, China), C=Combi-Blocks Inc., (San Diego,CA, USA)

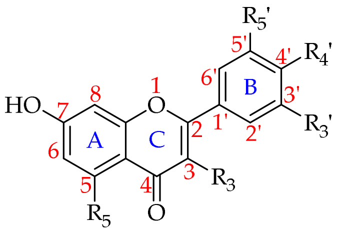

Supplementary Figure S2: Basic Flavonol Structure (see **10-17** in **Supplementary Table S2**)

Supplementary Table S2: Flavonol Standards Used in the Database Development (See **Supplementary Figure S2** for general structure)

**R3**	**R5**	**R3′**	**R4′**	**R5′**	**Code**	**Name**	**CAS No.**	**Supplier**	**Concentration (mg/mL) & Injection Volume** (μL)
-O-H	-H	-O-H	-O-H	-H	**10**	Fisetin	528-48-3	B	0.5, 5
-O-H	-O-H	-H	-H	-H	**11**	Galangin	548-83-4	D	0.5, 5
-O-H	-O-H	-O-Me	-O-H	-H	**12**	Isorhamnetin	480-19-3	D	0.5, 5
-O-H	-O-H	-H	-O-Me	-H	**13**	Kaempferide	491-54-3	B	0.5, 5
-O-H	-O-H	-H	-O-H	-H	**14**	Kaempferol	520-18-3	C	0.5, 5
-O-H	-O-H	-O-H	-O-H	-O-H	**15**	Myricetin	529-44-2	C	0.5, 5
-O-H	-O-H	-O-H	-O-H	-H	**16**	Quercetin	117-39-5	E	0.5, 5
-O-Rut	-O-H	-O-H	-O-H	-H	**17**	Rutin	153-18-4	C	0.5, 5

Legend: -H – hydride, -O-H – hydroxide, -O-Me – Methoxide, -O-Rut – rutoside, Suppliers: B=Angene International Ltd. (Nanjing, China), C=Combi-Blocks Inc., (San Diego,CA, USA), D= Wuhan ChemFaces Biochemical Co., Ltd. (Wuhan, Hubei, China), E= Alfa Aesar (Lancashire, UK)

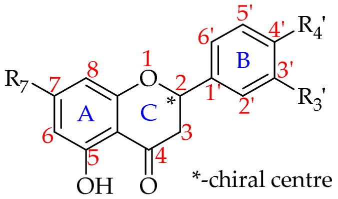

Supplementary Figure S3: Basic Flavanone Structure (see **18-23** in **Supplementary Table S3**)

Supplementary Table S3: Flavanone Standards Used in the Database Development (See **Supplementary Figure S3** for general structure)

**R7**	**R3′**	**R4′**	**Code**	**Name**	**CAS No.**	**Supplier**	**Concentration (mg/mL) & Injection Volume** (μL)
-O-H	-O-H	-O-Me	**18**	Hesperitin	520-33-2	C	0.5, 5
-O-Rut	-O-H	-O-Me	**19**	Hesperidin	520-26-3	C	0.5, 5
-O-H	-H	-O-H	**20**	Naringenin	67604-48-2	C	0.5, 5
-O-Rut	-H	-O-H	**21**	Naringin	10236-47-2	F	1.0, 5

-O-H	-H	-H	**22**	Pinocembrin	480-39-7	D	0.5, 5
-O-Me	-H	-O-H	**23**	Sakuranetin	2957-21-3	B	0.5, 5

Legend: -H – hydride, -O-H – hydroxide, -O-Me – Methoxide, -O-Rut – rutoside, Suppliers: B=Angene International Ltd. (Nanjing, China), C=Combi-Blocks Inc., (San Diego,CA, USA), D= Wuhan ChemFaces Biochemical Co., Ltd. (Wuhan, Hubei, China), F=AK Scientific, Inc. (CA, USA)

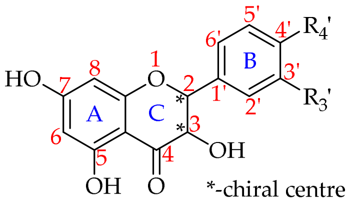

Supplementary Figure S4: Basic Flavanonol Structure (see **24-25** in **Supplementary Table S4**)

Supplementary Table S4: Flavanone Standards Used in the Database Development (See **Supplementary Figure S4** for general structure)

R3′	R4′	Code	Name	CAS No.	Supplier	Concentration (mg/mL) & Injection Volume (μL)
-H	-H	**24**	Pinobanksin	548-82-3	B	0.5, 5
-O-H	-O-H	**25**	Taxifolin	480-18-2	F	0.1, 0.5

Legend: -H – hydride, -O-H – hydroxide, Suppliers: B=Angene International Ltd. (Nanjing, China), F=AK Scientific, Inc. (CA, USA)

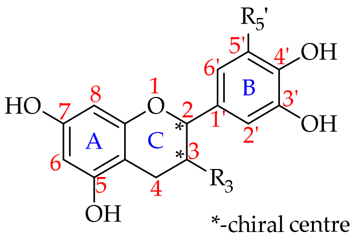

Supplementary Figure S5: Basic Flavan-3-ol Structure (see **26-29** in **Supplementary Table S5**)

Supplementary Table S5: Flavan-3-ol Standards Used in the Database Development (See **Supplementary Figure S5** for general structure)

R3	R5′	Code	CAS No.	Name	Supplier	Concentration (mg/mL) & Injection Volume (μL)
-O-H	-H	**26**	154-23-4	Catechin	B	0.5, 5
-O-H	-H	**27**	490-46-0	Epicatechin	D	0.5, 5
-O-H	-O-H	**28**	970-74-1	Epigallocatechin	C	0.5, 5
-O-Gall	-O-H	**29**	989-51-5	Epigallocatechin gallate	B	0.5, 5

Legend: -H – hydride, -O-H – hydroxide, -O-Gall-Gallate; Suppliers: B=Angene International Ltd. (Nanjing, China), C=Combi-Blocks Inc., (San Diego,CA, USA), D= Wuhan ChemFaces Biochemical Co., Ltd. (Wuhan, Hubei, China)

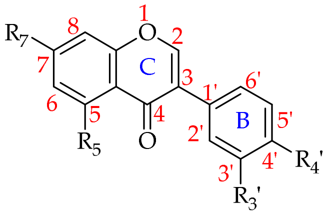

Supplementary Figure S6. Basic Isoflavonoid Structure (see **30-34** in **Supplementary Table S6**)

Supplementary Table S6: Isoflavonoid Standards used in the Database Development (See **Supplementary Figure S6** for general structure)

R5	R7	R3′	R4′	Code	Name	CAS No.	Supplier	Concentration (mg/mL) & Injection Volume (μL)
-O-H	-O-H	-H	-O-Me	**30**	Biochanin A	491-80-5	A	0.5, 5
-H	-O-H	-O-H	-H	**31**	Daidzein	486-66-8	A	0.25, 5
-H	-O-H	-H	-O-Me	**32**	Formononetin	485-72-3	B	0.5, 5
-O-H	-O-H	-H	-O-H	**33**	Genistein	446-72-0	C	0.5, 5
-O-H	-O-Glc	-H	-O-H	**34**	Genistin	529-59-9	B	0.5, 5

Legend: -H – hydride, -O-H – hydroxide, -O-Me – Methoxide, -O-Glc – glucoside, Suppliers : A= Sigma Aldrich (Castle Hill, NSW, Australia), B=Angene International Ltd. (Nanjing, China), C=Combi-Blocks Inc., (San Diego,CA, USA)

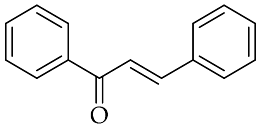

Supplementary Figure S7. Chalcone (see **35** in **Supplementary Table S7**)

Supplementary Table S7: t-Chalcone Standard Used in the Database Development (See **Supplementary Figure S7** for structure)

Code	Name	CAS No.	Supplier	Concentration (mg/mL) & Injection Volume (μL)
**35**	Chalcone	614-47-1	A	0.5, 5

Legend: Supplier: A= Sigma Aldrich (Castle Hill, NSW, Australia)

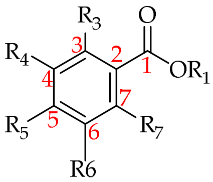

Supplementary Figure S8: Hydroxybenzoic Acid and its Derivatives (HBADs) (see **36-60 in Supplementary Table S8**)

Supplementary Table S8: Hydroxybenzoic Acid and its Derivatives (HBADs) Standards used in the Database Development (See **Supplementary Figure S8** for general structure)

OR1	R3	R4	R5	R	Code	Name	CAS No.	Supplier	Concentration (mg/mL) & Injection Volume (μL)
-H	-O-H	-O-H	-O-H	-H	**36**	2,3,4 Trihydrobenzoic acid	610-02-6	B	0.5, 5
-H	-O-Me	-O-Me	-O-Me	-H	**37**	2,3,4-TrimethoxyBenzoic acid	573-11-5	A	0.5, 5
-H	-O-Me	-H	-O-Me	-O-Me	**38**	2,4,5-TrimethoxyBenzoic Acid	490-64-2	A	0.5, 5
-H	-O-H	-H	-O-H	-H	**39**	3,5-Dihydroxybenzoic acid	99-10-5	A	0.5, 5
-H	-H	-H	-H	-H	**40**	Benzoic acid	65-85-0	G	2, 5
-H	-H	-H	-Isopropyl	-H	**41**	Cuminic acid	536-66-3	F	1, 5
See Figure 9					**42**	Ellagic acid	476-66-4	H	0.5, 20
-H	-H	-O-Me	-O-Me	-O-Me	**43**	Eudesmic acid	118-41-2	C	1, 5
-H	-H	-O-H	-O-H	-O-H	**44**	Gallic Acid	149-91-7	I	0.5, 5
-H	-O-H	-H	-H	-O-H	**45**	Gentisic acid	490-79-9	C	0.5, 10
-Me	-H	-O-Me	-O-gent	-O-Me	**46**	Leptosperin	N.I	J	0.5, 5
-Me	-H	-H	-O-H	-H	**47**	Methyl paraben	99-76-3	C	1, 5
-Me	-H	-O-Me	-O-H	-O-Me	**48**	Methyl syringate	884-35-5	C	0.5, 5
-Me	-H	-O-Me	-O-Me	-O-Me	**49**	Methyl-3,4,5-TMBe	1916-07-0	H	1, 5
-H	-H	-O-H	-H	-H	**50**	m-hydroxybenzoic acid	99-06-9	C	1, 5
-H	-H	-Me	-H	-H	**51**	m-Toluic Acid	99-04-7	A	0.5, 5
-H	-O-Me	-H	-H	-H	**52**	o-Anisic acid	579-75-9	A	0.5, 5
-H	-Me	-H	-H	-H	**53**	o-Toluic Acid	118-90-1	A	0.5, 3
-H	-H	-O-H	-O-H	-H	**54**	Protocatechuic acid	99-50-3	C	0.5, 5
-H	-H	-H	-O-H	-H	**55**	p-Hydroxybenzoic acid	99-96-7	C	0.5, 5
-H	-O-H	-O-H	-H	-H	**56**	Resorcylic acid	303-38-8	C	1, 5
-H	-O-H	-H	-H	-H	**57**	Salicylic acid	69-72-7	G	2, 5
-H	-H	-O-Me	-O-H	-O-Me	**58**	Syringic acid	530-57-4	C	0.5, 5
-H	-H	-O-Me	-O-H	-H	**59**	Vanillic acid	121-34-6	C	0.5, 5
-Me	-H	-O-Me	-O-H	-H	**60**	Vanillic acid methyl ester	3943-74-6	C	0.5, 5

Legend: -H – hydride, -O-H – hydroxide, -O-Me – Methoxide, -Me – methyl, -O-Gent – gentibioside, TMBe – trimethoxybenzoate, Suppliers : A= Sigma Aldrich (Castle Hill, NSW, Australia), B=Angene International Ltd. (Nanjing, China), C=Combi-Blocks Inc., (San Diego,CA, USA), F= AK Scientific, Inc. (CA, USA), G= Chem Supply Australia Pty Ltd (Port Adelaide, SA, Australia), H=Sigma-Aldrich (St. Louis, MO, USA), I= Ajax Finechem Pvt. Ltd., (Sydney, Australia), J=Kindly donated by Dr Jonathan Stephens (Auckland University, New Zealand)

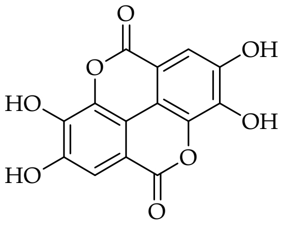

Supplementary Figure S9: Ellagic Acid (42)

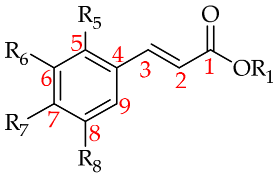

Supplementary Figure S10: Hydroxycinnamic Acid and its Derivatives (HCADs) (see **61-76** in **Supplementary Table S9**)

Supplementary Table S9: Hydroxycinnamic Acid and its Derivatives (HCADs) Standards used in the Database Development (See **Supplementary Figure S10** for general structure)

**OR1**	**R5**	**R6**	**R7**	**R8**	**Code**	**Name**	**CAS No.**	**Supplier**	**Concentration (mg/mL) & Injection Volume (μL)**
-H	-H	-O-Me	-O-Me	-H	**61**	3,4-DMCA	2316-26-9	C	1, 5
-H	-H	-O-H	-O-H	-H	**62**	Caffeic acid	331-39-5	C	0.5, 5
-phenyl	-H	-O-H	-O-H	-H	**63**	CAPE	104594-70-9	A	0.5, 5
-QA (3)	-H	-O-H	-O-H	-H	**64**	Chlorogenic acid	327-97-9	C	0.5, 5
-H	-H	-O-Me	-O-H	-H	**65**	Ferulic acid	537-98-4	C	0.5, 5
-H	-H	-O-H	-O-Me	-H	**66**	Isoferulic acid	537-73-5	F	1, 5
-H	-H	-O-H	-H	-H	**67**	m-Coumaric acid	14755-02-3	C	1.7, 5
-Me	-H	-O-Me	-O-H	-H	**68**	Methyl ferulate	2309-07-1	C	1, 5
-QA (3)	-H	-O-H	-O-H	-H	**69**	Neochlorogenic Acid	906-33-2	A	0.5, 5
-H	-O-H	-H	-H	-H	**70**	o-Coumaric acid	614-60-8	C	0.5, 5
-H	-H	-H	-O-H	-H	**71**	p-Coumaric acid	501-98-4	C	0.5, 5
-H	-H	-H	-O-Me	-H	**72**	p-Methoxycinnamic acid	830-09-1	A	0.5, 5
-3,4-DHPLA	-H	-O-H	-O-H	-H	**73**	Rosmarinic acid	20283-92-5	C	0.5, 5
-H	-H	-O-Me	-O-H	-Me	**74**	Sinapic acid	530-59-6	C	0.5, 5
-H	-H	-H	-H	-H	**75**	t-Cinnamic acid	140-10-3	C	0.5, 5
-Me	-H	-H	-O-H	-H	**76**	t-p-CAME	19367-38-5	B	0.5, 5

Legend: -H – hydride, -O-H – hydroxide, -O-Me – Methoxide, -Me – methyl, QA=quinic acid, DMCA – Dimethoxycinnamic acid, CAPE – Caffeic acid phenethyl ester, t-p-CAME – Coumaric acid methyl ester, Suppliers: A= Sigma Aldrich (Castle Hill, NSW, Australia), B=Angene International Ltd. (Nanjing, China), C=Combi-Blocks Inc., (San Diego,CA, USA), F= AK Scientific, Inc. (CA, USA)

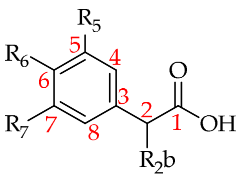

Supplementary Figure S11: Hydroxyphenylacetic Acid and Derivatives (HPAAD) (see **77-82** in **Supplementary Table S10**)

Supplementary Table S10: Hydroxyphenylacetic Acid and Derivatives (HPAAD) Standards used in the Database Development (See **Figure S11** for general structure)

R2b	R5	R6	R7	Code	Name	CAS No.	Supplier	Concentration (mg/mL) & Injection Volume (μL)
-H	-H	-O-H	-O-H	**77**	3,4-Dihydroxyphenylacetic acid	102-32-9	A	0.5, 5
-H	-O-H	-H	-O-H	**78**	Homogentisic acid	451-13-8	D	0.5, 5
-H	-O-Me	-O-H	-H	**79**	Homovanillic acid	306-08-1	C	1, 5
-O-H	-H	-H	-H	**80**	Mandelic acid	90-64-2	A	0.5, 10
-H	-H	-H	-H	**81**	Phenylacetic acid	103-82-2	K	2, 5
-H	-H	-O-H	-H	**82**	p-Hydroxyphenylacetic acid	156-38-7	A	0.5, 20

Legend: -H – hydride, -O-H – hydroxide, -O-Me – Methoxide, -Me – methyl, Suppliers: A= Sigma Aldrich (Castle Hill, NSW, Australia), C= Combi-Blocks Inc., (San Diego,CA, USA), D= Wuhan ChemFaces Biochemical Co., Ltd. (Wuhan, Hubei, China), K= Fluka AG (Buchs, Sankt Gallen, Switzerland)

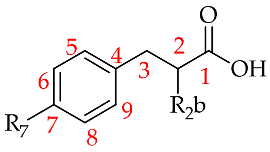

Supplementary Figure S12: Hydroxyphenyllactic Acid and Derivatives (HPLAD) (see **83-85** in **Supplementary Table S11**) and Hydroxyphenylpropanoic Acid and Derivatives (HPPAD) (see **86-87** in **Supplementary Table S11**)

Supplementary Table S11: Hydroxyphenyllactic Acid and Derivatives (HPLAD) and Hydroxyphenylpropanoic Acid and Derivatives (HPPAD) Standards used in the Database Development (See **Supplementary Figure S12** for general structure)

R2b	R7	Subclass	Code	Name	CAS No.	Supplier	Concentration (mg/mL) & Injection Volume (μL)
-O-H	-H	HPLAD	**83**	DL-β-Phenyllactic acid	828-01-3	C	0.5, 5
-O-H	-O-H	HPLAD	**84**	DL-p hydroxyphenyllactic acid	306-23-0	A	2, 5
-O-H	-O-Me	HPLAD	**85**	p-Methoxyphenyllactic acid	N.I.	C	0.5, 20
-H	-H	HPPAD	**86**	3-Phenyl propionic acid	501-52-0	C	1, 5
-H	-O-H	HPPAD	**87**	Phloretic acid	501-97-3	C	1, 5

Legend: -H – hydride, -O-H – hydroxide, -O-Me – Methoxide, N.I. – No information, Suppliers: A= Sigma Aldrich (Castle Hill, NSW, Australia), C= Combi-Blocks Inc., (San Diego,CA, USA)

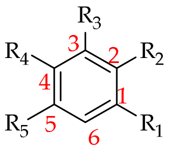

Supplementary Figure S13: Other phenolic compounds (see **88-101** in **Supplementary Table S12**)

Supplementary Table S12: Other/Miscellaneous Phenolic Standards used in the Database Development (See **Supplementary Figure S12** for general structure)

R1	R2	R3	R4	R5	Subclass	Code	Name	CAS No.	Supplier	Concentration & Injection Volume (μL)
-O-H	-O-Me	-H	-vinyl	-H	AMPh	**88**	2-Methoxy-4-vinylphenol	7786-61-0	C	2 mg/mL, 3
-O-H	-H	-H	-O-Me	-H	AMPh	**89**	p-Methoxyphenol	150-76-5	K	1.0 mg/mL, 10
-O-H	-O-H	-H	-H	-Me	APh	**90**	4-Methylpyrocatechol	452-86-8	C	1.0 mg/mL, 5
-O-H	-H	-Me	-Me	-Me	APh	**91**	Isopseudocumenol	527-54-8	C	0.5 mg/mL, 5
-O-H	-Isopr	-H	-H	-Me	APh	**92**	Thymol	89-83-8	A	0.5 mg/mL, 5
-O-H	-H	-H	-Amide	-H	p-AmPh	**93**	Acetaminophen	103-90-2	H	1.0 mg/mL, 5
-O-H	-O-H	-H	-H	-H	Phenol	**94**	Pyrocatechol	120-80-9	G	1.0 mg/mL, 5
-O-H	-O-H	-O-H	-H	-H	Phenol	**95**	Pyrogallol	87-66-1	K	1.0 % v/v, 5
-C(=O)H	-Me	-H	-H	-H	HBzd	**96**	2-Methylbenzaldehyde	529-20-4	C	1.0 % v/v, 5
-C(=O)H	-H	-H	-O-Me	-H	HBzd	**97**	p-Anisaldehyde	123-11-5	L	0.5 mg/mL, 5
-C(=O)H	-H	-O-H	-O-H	-H	HBzd	**98**	Protocatechualdehyde	139-85-5	C	0.5 mg/mL, 5
-C(=O)H	-H	-O-Me	-O-H	-H	HBzd	**99**	Vanillin	121-33-5	H	0.5mg/mL, 3
-ethanone	-O-H	-H	-H	-H	HAPhn	**100**	2'-Hydroxyacetophenone	118-93-4	A	0.5 mg/mL, 5
-ethanone	-O-Me	-H	-H	-H	HAPhn	**101**	2'-Methoxyacetophenone	579-74-8	A	2.0 mg/mL, 3

Legend: -H – hydride, -O-H – hydroxide, -O-Me – Methoxide, -Isopr – isopropyl, -C(=O) H – aldehyde group, Subclass: AMPh – alkylmethoxyphenol, Aph – alkylphenol, HBzd – hydroxybenzaldehyde, HAPhn – hydroxyacetophenone, Suppliers: A= Sigma Aldrich (Castle Hill, NSW, Australia), C= Combi-Blocks Inc., (San Diego,CA, USA), G= Chem Supply Australia Pty Ltd (Port Adelaide, SA, Australia), H= Sigma-Aldrich (St. Louis, MO, USA), K=Fluka AG (Buchs, Sankt Gallen, Switzerland), L= Acros Organics (Carlsbad, CA, USA)

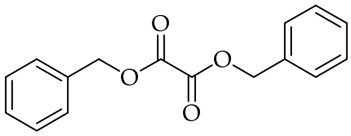

Supplementary Figure S14: Dibenzyl oxalate (see **102** in **Supplementary Table S13**)

Supplementary Table S13: Oxalate ester Standard used in the Database Development (See **Supplementary Figure S14** for structure)

Code	Name	CAS No.	Supplier	Concentration (mg/mL) and Injection Volume (μL)
**102**	Dibenzyl oxalate	7579-36-4	C	1.0, 10

Supplier: C= Combi-Blocks Inc., (San Diego,CA, USA)

Supplementary Figure S15: Structure of the non-phenolic compounds (see **103-107** in **Supplementary Table S14**)

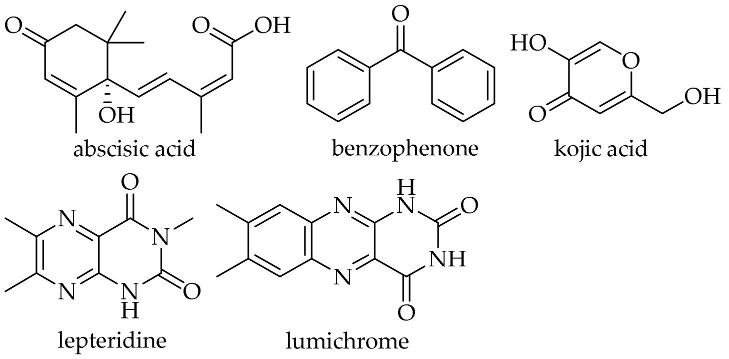

Supplementary Table S14: Non-phenolic compounds used in the Database Development (See **Supplementary Figure S15** for structures)

**Subclass**	**Name**	**Code**	**CAS No.**	**Supplier**	**Concentration (mg/mL) and Injection Volume (μL)**
Non Phenolic	Absiscic acid	**103**	21293-29-8	A	0.5, 5
Non Phenolic	Benzophenone	**104**	119-61-9	G	0.5, 20
Non Phenolic	Kojic acid	**105**	501-30-4	A	0.5, 5
Non Phenolic	Lepteridine	**106**	N.I.	J	0.5, 5
Non Phenolic	Lumichrome	**107**	1086-80-2	A	0.5, 5

Legend: N.I. – no information, Suppliers: A=Sigma Aldrich (Castle Hill, NSW, Australia), G=Chem Supply Australia Pty Ltd (Port Adelaide, SA, Australia), J=Kindly donated by Dr Jonathan Stephens (Auckland University, New Zealand)

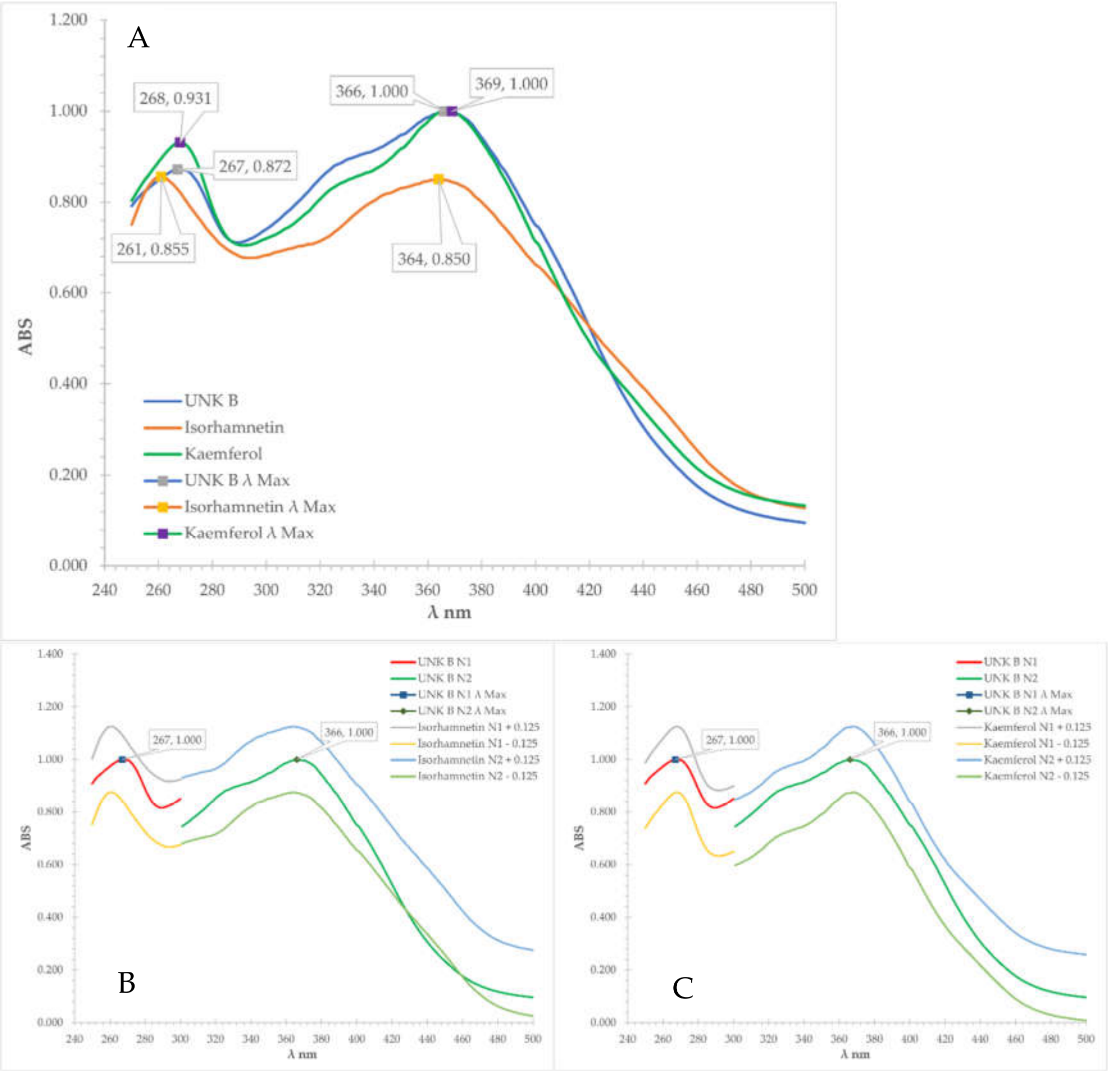

Supplementary Figure S16 A-C. UV-Vis spectra (prior to derivatisation) overlay of unknown B vs isorhamnetin and kaempferol (A) and UV-Vis (prior to derivatisation) spectra overlay of unknown A vs the ±0.125 AU of isorhamnetin (B) and vs ±0.125 AU of kaempferol (C)

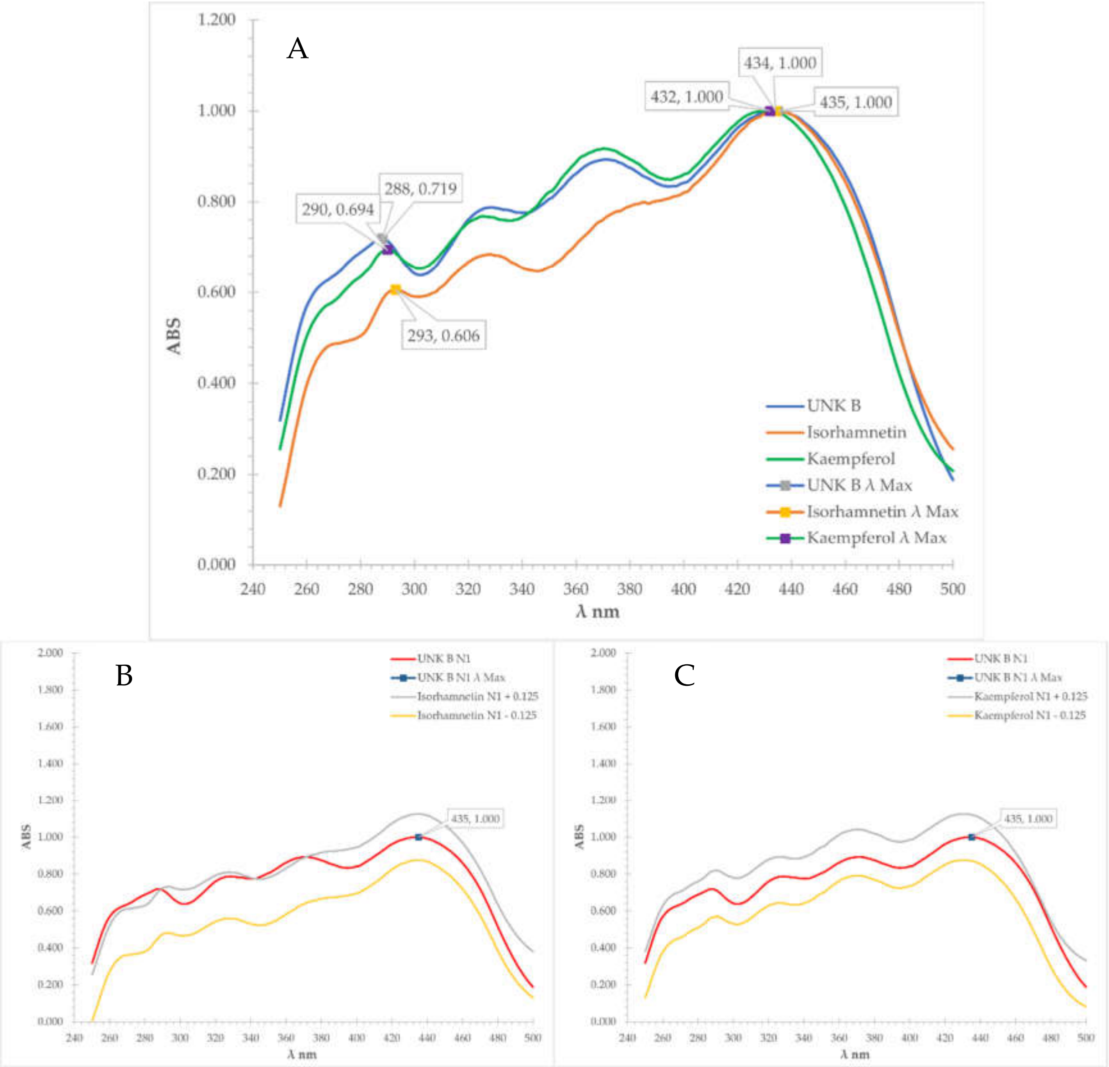

Supplementary Figure S17 A-C. UV-Vis spectra (after derivatisation with NP-PEG reagent) overlay of unknown B vs isorhamnetin and kaempferol (A) and UV-Vis (after derivatisation with NP-PEG reagent) spectra overlay of unknown A vs the ±0.125 AU of isorhamnetin (B) and vs ±0.125 AU of kaempferol (C)

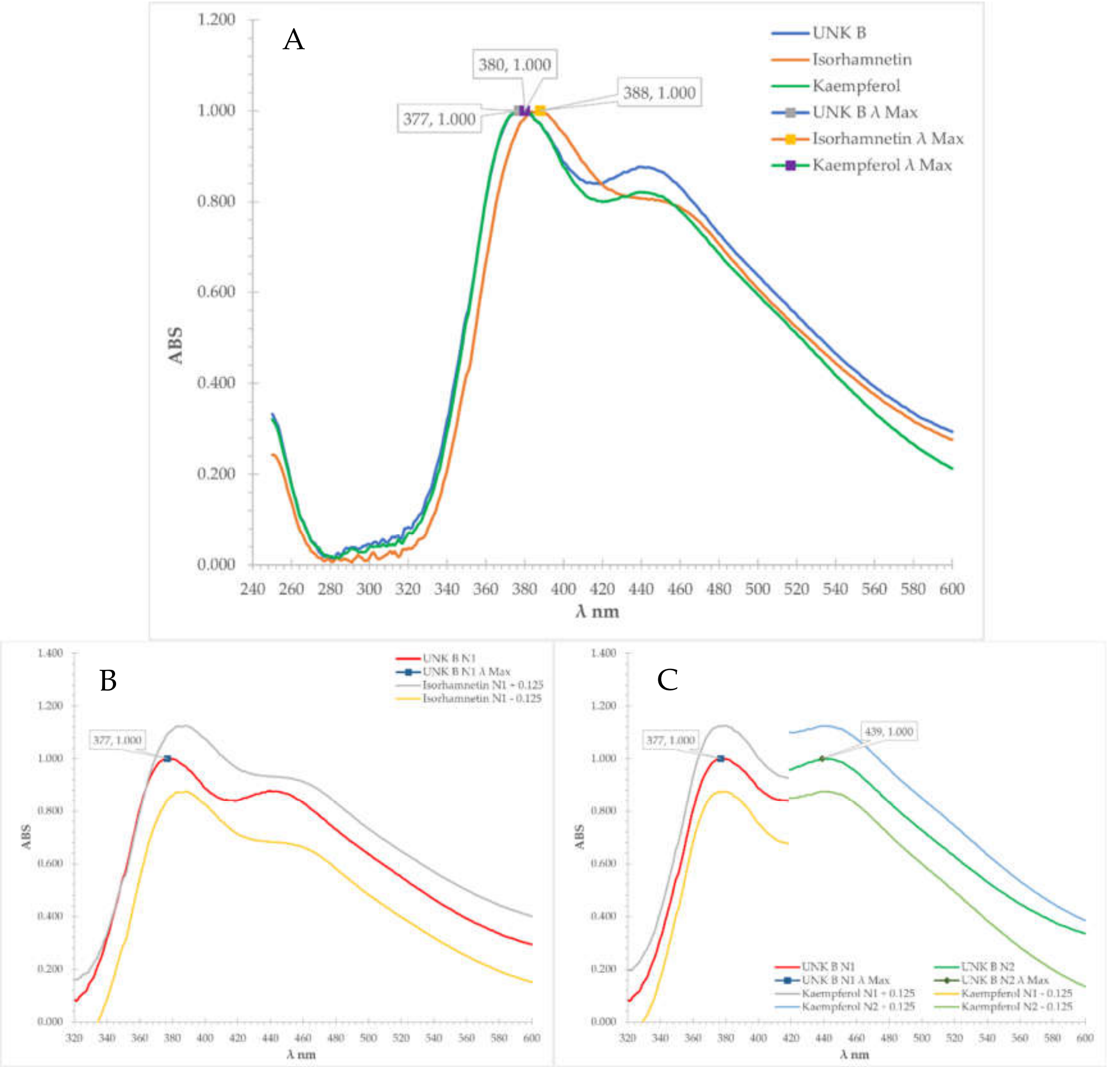

Supplementary Figure S18 A-C. UV-Vis spectra (after derivatised with VSA reagent) overlay of unknown B vs isorhamnetin and kaempferol (A) and UV-Vis (after derivatised with VSA reagent) spectra overlay of unknown A vs the ±0.125 AU of isorhamnetin (B) and vs ±0.125 AU of kaempferol (C)

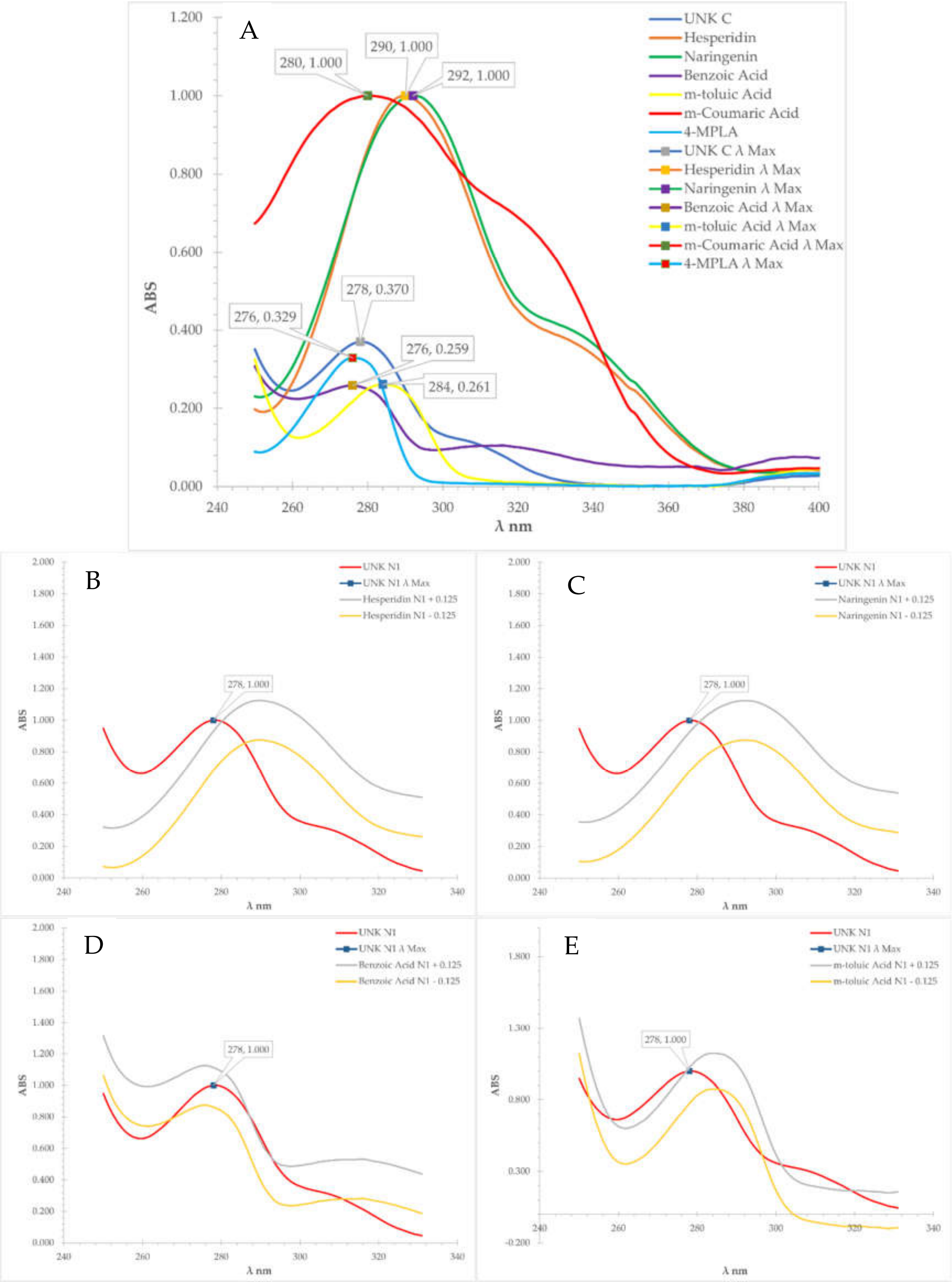
A
UNK C
Hesperidin
Naringenin
Benzoic Acid
m-toluic Acid
m-Coumaric Acid
4-MPLA
UNK C λ Max
Hesperidin λ Max
Naringenin λ Max
Benzoic Acid λ Max
m-toluic Acid λ Max
m-Coumaric Acid λ Max
4-MPLA λ Max
280, 1.000
290, 1.000
292, 1.000
278, 0.370
276, 0.329
276, 0.259
284, 0.261
ABS
λ nm
B
UNK N1
UNK N1 λ Max
Hesperidin N1 + 0.125
Hesperidin N1 - 0.125
278, 1.000
C
UNK N1
UNK N1 λ Max
Naringenin N1 + 0.125
Naringenin N1 - 0.125
278, 1.000
D
UNK N1
UNK N1 λ Max
Benzoic Acid N1 + 0.125
Benzoic Acid N1 - 0.125
278, 1.000
E
UNK N1
UNK N1 λ Max
m-toluic Acid N1 + 0.125
m-toluic Acid N1 - 0.125
278, 1.000

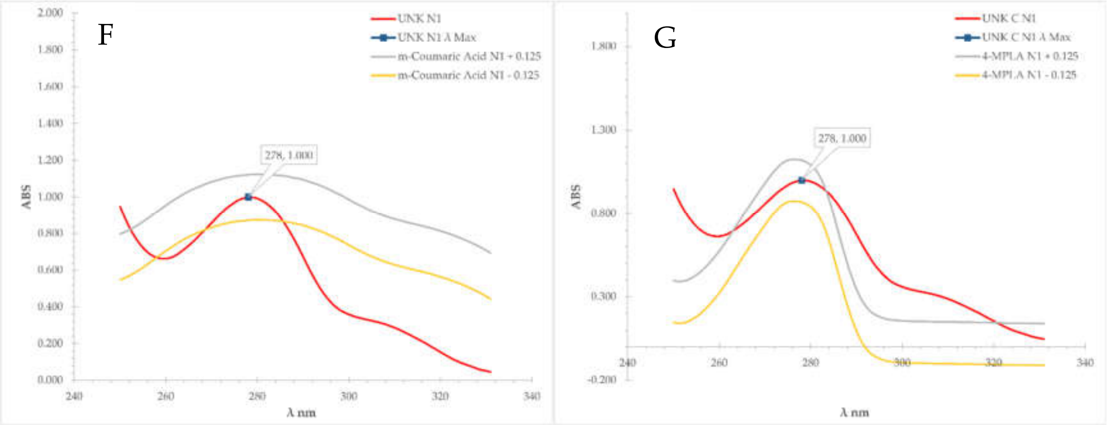

Supplementary Figure S19 A-G. UV-Vis spectra (prior to derivatisation) overlay of unknown C vs the match compounds and UV-Vis (prior to derivatisation) spectra overlay of unknown A vs the ±0.125 AU of hesperitin (B), vs ±0.125 AU of naringenin (C), vs ±0.125 AU of benzoic acid (D), ±0.125 AU of m-toluic acid (E), ±0.125 AU of m-coumaric acid (F), and ±0.125 AU of p-MPLA (G)

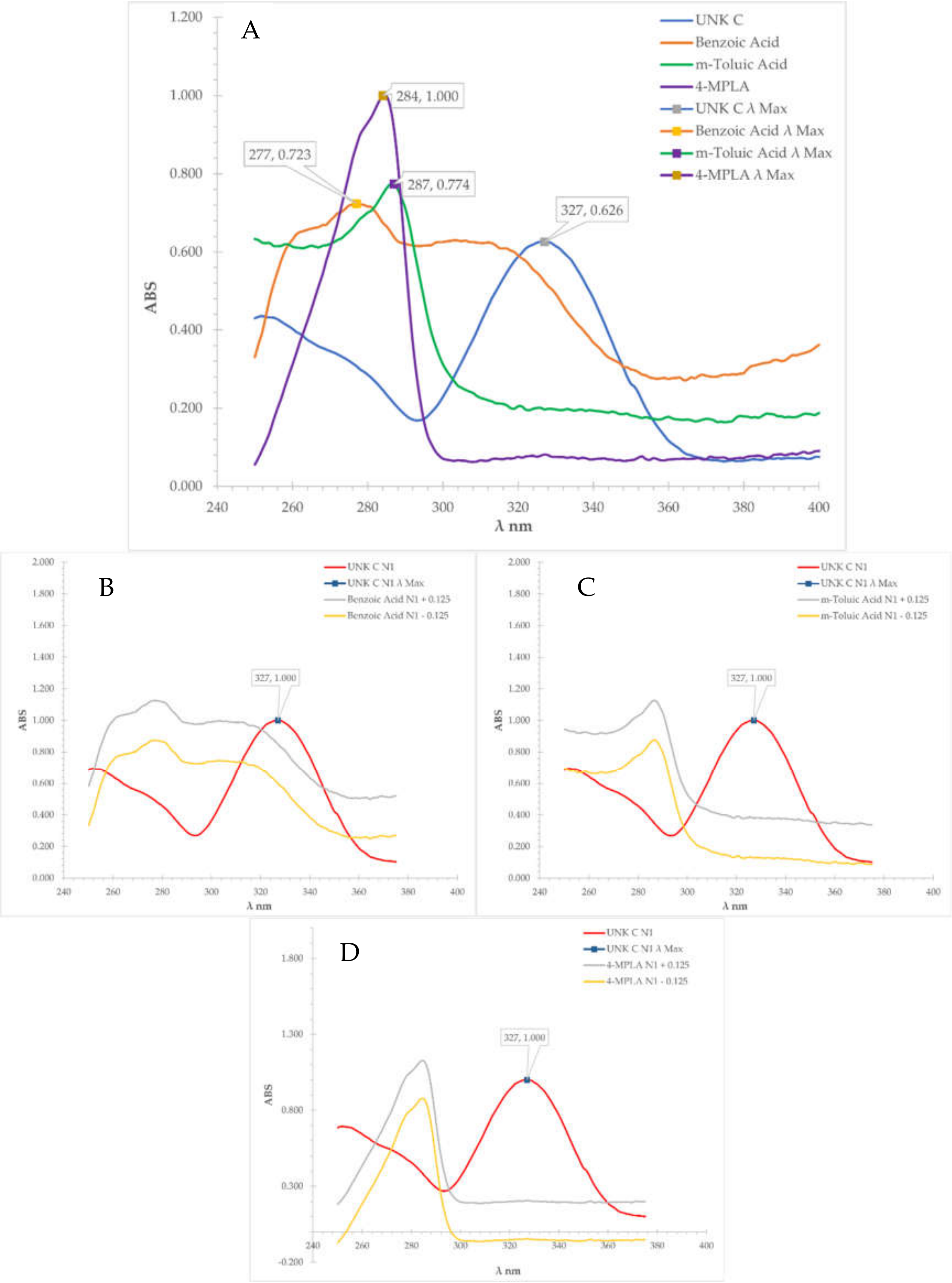

Supplementary Figure S20 A-D. UV-Vis spectra (after derivatisation with NP-PEG reagent) overlay of unknown C vs benzoic acid (B), m-toluic acid (C), and 4-MPLA (D) and UV-Vis (after development) spectra overlay of unknown A vs the ±0.125 AU of benzoic acid (B), ±0.125 AU of m-toluic acid (C), and ±0.125 AU of p-MPLA (D)

Supplementary Table S15. Summary of the data used to determine the identity of the unknown bands in Manuka honey (Database 1A)

Name and Code	Rf 1	H° DEV 254 nm	H° DEV 366 nm	H° NP 366 nm	Fl DEV λ	Fl DEV λ m	UV DEV λ_1	UV DEV λ_2	UV DEV λ_3	Fl NP λ	UV NP λ1
LS	0.02	140	196	195	223	257	267	0	0	238	319
LS	0.077	141	200	195	222	259	283	0	0	252	327
LS	0.134	141	181	201	225	262	260	0	0	250	327
LS	0.199	140	194	191	223	240	282	0	0	250	324
LS	0.244	140	192	195	224	259	288	0	0	251	327
LS	0.319	143	187	197	224	255	328	0	0	251	331
LS	0.392	139	196	195	222	253	282	0	0	246	328
LS	0.467	142	192	196	219	261	264	358	0	250	318
LS	0.513	140	197	199	222	233	276	0	0	248	316
LS	0.543	141	195	196	226	233	277	0	0	248	319
LS	0.635	140	206	200	229	260	275	0	0	246	292
LS	0.685	141	204	198	225	256	261	0	0	249	324

Legend: **Rf1** – retention factor in MPA, **H° DEV 254 nm** – hue equivalent at 254 nm prior to derivatisation, **H° DEV 366 nm** – hue equivalent at 366 nm prior to derivatisation, **H° NP 366 nm** – hue equivalent at 366 nm after derivatisation w/ NP-PEG derivatisation reagent, **Fl DEV λ**– fluorescence λ max prior to derivatisation, **Fl DEV λ m** – fluorescence λ min prior to derivatisation, **UV DEV $\lambda_{1\text{-}3}$** – UV-Vis λ max prior to derivatisation, **Fl NP λ**– fluorescence λ max after derivatisation with NP-PEG reagent, **UV NP $\lambda_{1\text{-}3}$**– UV-Vis λ max after derivatisation with NP-PEG reagent

Supplementary Table S16. Summary of the data used to determine the identity of the unknown bands in Manuka honey (Database 1B)

Name and Code	Rf 1	H° DEV 254 nm	H° DEV 366 nm	H° VSA 366 nm	H° T VSA	Fl DEV λ	Fl DEV λ m	UV DEV λ1	UV DEV λ2	UV DEV λ3	Fl VS λ	UV VS λ
LS	0.02	140	196	203	23	223	257	267	0	0	249	447
LS	0.077	141	200	200	23	222	259	283	0	0	249	372
LS	0.134	141	181	199	23	225	262	272	0	0	249	378
LS	0.199	140	194	201	26	223	240	282	0	0	249	439
LS	0.244	140	192	199	19	224	259	288	0	0	248	367
LS	0.319	143	187	201	17	224	255	328	0	0	248	365
LS	0.392	139	196	203	10	222	253	282	0	0	248	369
LS	0.467	142	192	202	29	219	261	264	358	0	249	368
LS	0.513	140	197	205	21	222	233	281	0	0	248	368
LS	0.543	141	195	206	9	226	233	277	0	0	249	367
LS	0.635	140	201	203	14	229	260	275	0	0	248	368
LS	0.685	141	205	202	21	225	256	261	0	0	249	368

Legend: **Rf1** – retention factor in **MPA**, **H° DEV 254 nm** – hue and colour equivalent at 254 nm prior to derivatisation, **H° DEV 366 nm** – hue and colour equivalent at 366 nm prior to derivatisation, **H° VS 366 nm** – hue and colour equivalent at 366 nm after derivatisation w/ VSA derivatisation reagent, **H° T VS** – hue and colour equivalent at transmittance in white light after derivatisation w/ VSA derivatisation reagent; **Fl DEV λ max**– fluorescence λ max prior to derivatisation, **Fl DEV λ m**– fluorescence λ min prior to derivatisation, **UV DEV λ_{1-3}** – UV-Vis λ max prior to derivatisation, **Fl VS λ**– fluorescence λ max after derivatisation with VSA reagent, **UV VS λ**— UV-Vis λ max after derivatisation with VSA reagent

Supplementary Table S17. Summary of the data used to determine the identity of the unknown bands in Manuka honey (Database 2A)

Name and Code	Rf 2	H° DEV 254 nm	H° DEV 366 nm	H° NP 366 nm	Fl DEV λ_1	Fl DEV m_1	UV DEV λ_1	UV DEV λ_2	UV DEV λ_3	Fl NP λ1	UV NP λ1
LS	0.021	144	200	196	224	258	285	0	0	246	309
LS	0.081	140	170	188	221	261	276	0	0	250	322
LS	0.100	140	194	198	211	259	281	0	0	246	315
LS	0.121	140	193	200	211	259	295	0	0	245	315
LS	0.150	140	196	199	223	261	281	0	0	248	314
LS	0.220	143	187	194	226	254	326	0	0	243	322
LS	0.247	140	194	199	221	254	250	334	0	246	325
LS	0.310	142	190	200	216	258	284	0	0	246	313
LS	0.349	139	197	197	223	233	277	0	0	247	315
LS	0.425	142	193	171	224	257	261	0	0	246	315
LS	0.470	141	203	201	224	244	296	0	0	246	294
LS	0.510	140	205	201	236	260	276	0	0	243	291
LS	0.550	141	192	200	224	259	276	0	0	246	291
LS	0.603	140	205	206	227	253	303	0	0	246	322

Legend: **Rf2** – retention factor in MPB, **H° DEV 254 nm** – hue equivalent at 254 nm prior to derivatisation, **H° DEV 366 nm** – hue equivalent at 366 nm prior to derivatisation, **H° NP 366 nm** – hue equivalent at 366 nm after derivatisation w/ NP-PEG derivatisation reagent, **Fl DEV λ**– fluorescence λ max prior to derivatisation, **Fl DEV λ m** – fluorescence λ min prior to derivatisation, **UV DEV λ_{1-3}** – UV-Vis λ max prior to derivatisation, **Fl NP λ**– fluorescence λ max after derivatisation with NP-PEG reagent, **UV NP λ_{1-3}**– UV-Vis λ max after derivatisation with NP-PEG reagent

Supplementary Table S18. Summary of the data used to determine the identity of the unknown bands in Manuka honey (Database 2B)

Name and Code	Rf 2	H° DEV 254 nm	H° DEV 366 nm	H° VSA 366 nm	H° T VSA	Fl DEV λ1	Fl DEV m1	UV DEV λ1	UV DEV λ2	UV DEV λ3	Fl VS λ2	UV VS λ1
LS	0.021	144	200	201	21	224	260	285	0	0	251	439
LS	0.081	140	170	205	24	221	261	276	0	0	252	435
LS	0.1	140	194	204	18	211	259	281	0	0	252	372
LS	0.121	140	193	200	17	211	259	266	0	0	247	451
LS	0.15	140	196	204	16	225	257	281	0	0	250	473
LS	0.22	143	187	205	4	226	254	326	0	0	248	368
LS	0.247	140	194	205	7	218	252	250	334	0	248	477
LS	0.31	142	190	200	28	215	261	284	0	0	247	445
LS	0.349	139	197	207	7	223	233	277	0	0	251	451
LS	0.425	142	193	206	60	224	256	261	0	0	250	451
LS	0.47	141	203	202	12	224	243	296	0	0	248	481
LS	0.51	140	205	204	6	236	260	276	0	0	248	367
LS	0.55	141	192	202	13	224	259	276	0	0	248	361
LS	0.603	140	205	206	23	227	253	303	0	0	248	370

Legend: **Rf2** – retention factor in **MPB**, **H° DEV 254 nm** – hue and colour equivalent at 254 nm prior to derivatisation, **H° DEV 366 nm** – hue and colour equivalent at 366 nm prior to derivatisation, **H° VSA 366 nm** – hue and colour equivalent at 366 nm after derivatisation w/ VSA derivatisation reagent, **H° T VSA** – hue and colour equivalent at transmittance in white light after derivatisation w/ VSA derivatisation reagent; **Fl DEV λ max**– fluorescence λ max prior to derivatisation, **Fl DEV λ m**– fluorescence λ min prior to derivatisation, **UV DEV λ1-3** – UV-Vis λ max prior to derivatisation, **Fl VS λ**– fluorescence λ max after derivatisation with VSA reagent, **UV VS λ**— UV-Vis λ max after derivatisation with VSA reagent
